# Eco-friendly and efficient Friedel–Crafts acylation of activated arenes catalyzed with low-loaded ferric chloride in propylene carbonate as the solvent: scope and mechanistic insights

**DOI:** 10.1039/d5ra03638k

**Published:** 2025-09-01

**Authors:** Saif Eddine Cherif, Mayssa Chehaibi, Rafif Raddadi, Marie Fustier-Boutignon, Sami Lakhdar, Jamil Kraïem

**Affiliations:** a Laboratoire de Développement Chimique, Galénique et Pharmacologique des Médicaments, Faculté de Pharmacie de Monastir, Université de Monastir Rue Avicenne 5000 Monastir Tunisia jamil.kraiem@fphm.u-monastir.tn; b CNRS, Université Paul Sabatier, Laboratoire Hétérochimie Fondamentale et Appliquée (LHFA, UMR5069) 118 Route de Narbonne, 31062 Cedex 09 Toulouse France sami.lakhdar@univ-tlse3.fr

## Abstract

The Friedel–Crafts acylation of arenes is a fundamental reaction extensively employed in both academic research and industrial applications. A significant limitation of this reaction is the requirement for stoichiometric amounts of Lewis acid catalysts, which are typically sensitive and generate considerable waste. In this study, we present an improved catalytic approach for the Friedel–Crafts acylation of activated arenes. Using acyl chlorides and acid anhydrides as acylating agents, the reaction is efficiently catalyzed by 5 mol% iron(iii) chloride in propylene carbonate, an environmentally friendly solvent that outperforms traditional solvents in maintaining high reaction efficiency under catalytic conditions. This green methodology demonstrates high effectiveness and broad applicability, yielding aromatic ketones in good to excellent yields. Preliminary DFT calculations were carried out to rationalize the mechanism of the reaction.

## Abstract 2

The Friedel–Crafts acylation of arenes catalyzed with low-loaded ferric chloride under green conditions.

## Graphical abstract

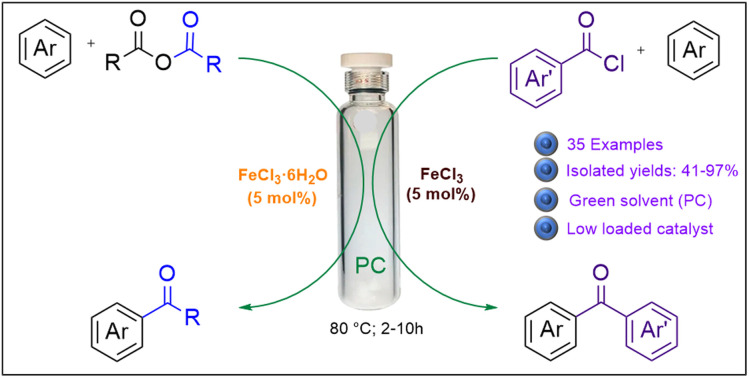

## Introduction

Aromatic ketones play a crucial role as essential intermediates and precursors in the production of pharmaceuticals, insecticides, cosmetics, and various other fine chemicals.^1^ Traditionally, these compounds are synthesized *via* Friedel–Crafts acylation of arenes, employing acyl chlorides and acid anhydrides as common acylating agents. This essential synthetic route involves the use of robust Lewis acid catalysts such as AlCl_3_,^2^ FeCl_3_,^3^ ZnCl_2_,^4^ and TiCl_4_,^5^ or strong Brønsted acids, such as H_2_SO_4_,^6^ HF^7^ and TfOH (Trifluoromethane sulfonic acid).^8^

The primary drawback associated with the classical Lewis acid (AlCl_3_, FeCl_3_, ZnCl_2_, or TiCl_4_) catalyzed process is the requirement for a stoichiometric or excess amount of the Lewis acid catalyst. This necessity affects both the economic feasibility and efficiency of the process. This is attributed to the Lewis basic nature of the resulting ketones, forming strong complexes through interaction between the ketone's oxygen atom and the metal halide. As a result, catalyst entrapment and deactivation occur, leading to the generation of significant amounts of toxic and polluting waste during the workup process.^9–11^ In fact, this major drawback concerns the FC acylation rather than the alkylation or the benzylation of arenes. As expected, there is no possible interaction between the Lewis acid and the products generated from the alkylation or the benzylation processes (hydrocarbons).^12,13^ As examples, we cite the work of Olah *et al.*^12^ which described the alkylation of anisole using 20 mol% of AlCl_3_ or 20 mol% of BF_3_·OEt_2_, and the benzylation of anisole and toluene in the presence of 20 mol% of TiCl_4_. In the same way, Beller *et al.*^13^ described the benzylation of arenes in the presence of 10 mol% of ferric chloride as the catalyst. These authors did not study the FC acylation reaction under catalytic conditions. Note that several examples of FC acylation using 1 or 2 equiv. of Lewis acid catalysts, such as AlCl_3_,^2,14,15^ BF_3_·OEt_2_ (ref. 16) and FeCl_3_,^3,17^ are described in the literature.

Significant advances in Friedel–Crafts acylation have led to catalytic methods employing both homogeneous and heterogeneous catalysts. Prominent among these are metal triflates, including, Ga(OTf)_3_,^18^ Bi(OTf)_3_,^19^ and Sc(OTf)_3_.^20^ In the same way, S. Lerch *et al.* described the acylation of activated arenes in ionic liquid containing 1 equiv. of trifluoromethanesulfonic amide anion ((Tf)_2_N^−^), in the presence of iron(iii) chloride. These authors have noted that when the triflic amide anion was replaced by the chloride anion in the ionic liquid, no reaction occurred.^21^ More insights into the catalytic behaviour of metal triflates in the FC acylation has recently been reported.^22^ This study assumed the formation of an acrylic triflic anhydride intermediate (R–C(O)–O–S(O)_2_-CF_3_) as the electrophilic species that react with the aromatic ring. Accordingly, this study illustrated that the process involves an activation with the triflate moiety rather than a Lewis acid catalysis. Thus, we believe that the triflic amide may act a similar manner to catalyze the FC acylation described by Larch *et* al.^21^ Notably, triflic acid is already used as a catalyst for FC acylation.^8^

While these catalysts have demonstrated satisfactory performance in Friedel–Crafts acylation, they are nonetheless costly and necessitate the use of toxic solvents such as nitromethane, chlorobenzene, and acetonitrile. Moreover, metal triflates are prepared from toxic and costly triflic acid (TfOH) and transition metals. The high cost of metal triflates and their susceptibility to degradation in the presence of air and moisture could hinder their practical application on an industrial scale.

In recent years, zeolites, clays, hetero polyacids, and Nafion have also been widely studied as heterogeneous catalysts in the FC acylation of arenes.^11^ Although these catalysts are ecologically advantageous, their use in industry still encounters some problems, due essentially to the adsorption of the ketone produced by the reaction on the active sites of the catalyst, to the formation of byproducts, and to the expensive recovering and reactivating of the catalyst.^9^

Based on our literature review of FC acylation, we can make the following observations: (i) the benzoylation of aromatics with benzoyl chloride, in the presence of graphite A (adsorbent), under high-energy conditions (MW: 150 w, 330 °C), led to a total conversion of the reactants into the corresponding ketone.^23^ However, it turned out that graphite A, whose purity is 99.1%, contains 0.41% Fe and 0.02% Al.^23^ Consequently, when the reaction was redone with graphite B (Purity: 99.9%; 0.007% Fe and 0.002 Al) the results were very different and no acylation of aromatics has taken place. Hence, the catalytic effect of the graphite A is due to the presence of iron-based impurities. These findings illustrate that the FC reaction requires the use of a catalyst such as a Lewis acid. (ii) Classical Lewis acids have shown good efficiency in the benzoylation of anisole with benzoyl chloride under MW irradiation (300 w, 150–170 °C).^23,24^ The increase in the catalytic activity of these Lewis acids can be explained by the effect of the high energetic conditions, which frees the catalyst from the ketone-metal complex. In return, excessive heating may lead to decompositions, degradations and formation of by-products. Consequently, this may not be suitable for industrial scale preparations. (iii) Recent efforts to enhance this process have primarily focused on ecological considerations, including the development and adoption of sustainable bio-based solvents, rather than prioritizing improvements in catalytic efficiency.^3,17^

In recent years, increasing awareness of the impact of industrial activities on the environment has led chemists to develop new chemical methods that are safer, more cost-effective and more respectful of the environment. Given the importance of Friedel–Crafts (FC) acylation as a simple, effective, and low-cost process for producing aromatic ketones, its negative environmental impact—mainly due to the use of stoichiometric or excess amounts of Lewis acids—may limit its application, especially on an industrial scale.

For example, the production of 1000 kg of 4-methoxyacetophenone requires 800 to 1600 kg of AlCl_3_. This encouraged us to revisit Friedel–Crafts acylation with the aim of developing new, efficient, and eco-friendly methods and this: (i) by using available, less-toxic and non-expensive Fe^III^ catalysts; (ii) by improving its catalytic activity in order to avoid the formation of a large amount of toxic waste from metal salts used in excessive quantities; (iii) by handling under green conditions. Note that ferric salts have shown effectiveness in several reactions,^25^ including the rearrangement of oxaziridines,^26^ acylation of weak nucleophiles,^13,27^ and etherification of alcohols.^28^

Herein, we present an efficient and environmentally friendly method for the synthesis of aromatic ketones from activated arenes.

This approach utilizes acyl chlorides and acid anhydrides as acylating agents in the presence of low-loaded iron(iii) chloride catalyst (5 mol%). Additionally, we have shown that propylene carbonate (PC) outperforms traditional solvents in maintaining high reaction efficiency under catalytic conditions. Based on preliminary DFT calculations, a plausible mechanism was discussed.

## Results and discussion

We began by conducting a systematic study aimed at identifying the optimal conditions for the acylation of arenes. Several reaction parameters were investigated, including the choice of solvent, temperature, catalyst type, and the catalyst-to-substrate ratio. Mesitylene (1,3,5-trimethylbenzene, 1a) and acetic anhydride (2a) were selected as the model substrates. The results of this investigation are summarized in Table 1.

**Table 1:** Optimization of the reaction conditions^a^

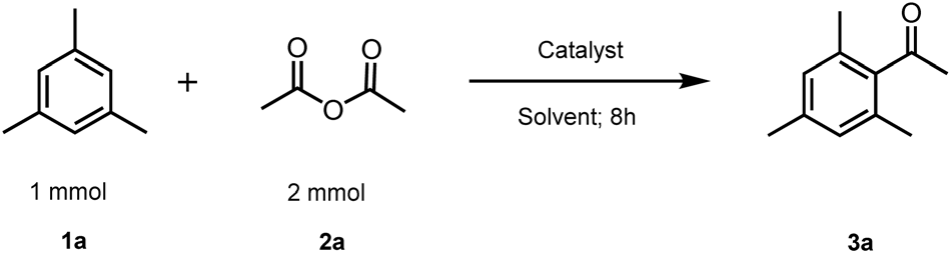				
Entry	Cat^b^ (mol%)	Solvent	*T* (°C)	Conv^c^ (%)
1	—	PC^d^	80	NR
2	AlCl_3_ (5)	—	80	11
3	AlCl_3_ (5)	PC	80	10
4	ZnCl_2_ (5)	PC	80	22
5	CuCl_2_ (5)	PC	80	31
6	Fe(TFA)_3_ (5)	PC	80	Trace
7	Fe(acac)_3_ (5)	PC	80	Trace
8	Fe_2_(SO_4_)_3_·5H_2_O (5)	PC	80	Trace
9	FeCl_3_ (5)	PC	80	88
**10**	**FeCl**_**3**_ **·6H**_**2**_ **O (5)**	**PC**	**80**	**95**
11	FeCl_3_·6H_2_O (2)	PC	80	82
12	FeCl_3_·6H_2_O (4)	PC	80	94
13	FeCl_3_·6H_2_O (10)	PC	80	95
14	FeCl_3_·6H_2_O (5)	PC	22	24
15	FeCl_3_·6H_2_O (5)	—	80	87
16	FeCl_3_·6H_2_O (5)	DEC^e^	80	88
17	FeCl_3_·6H_2_O (5)	DMC^f^	80	74
18	FeCl_3_·6H_2_O (5)	EtOAc	80	47
19	FeCl_3_·6H_2_O (5)	CH_2_Cl_2_	80	38
20	FeCl_3_·6H_2_O (5)	CH_3_CN	80	33

a Reaction conditions: 1a (1 mmol), 2a (2 mmol), catalyst (x mol%), solvent (1 mL). The mixture was stirred in a 8 mL pressure tube for 8 h at 80 °C.

b Ratio of the catalyst with respect to mesitylene.

c Determined by the ^1^H NMR analysis of the crude product.

d Propylene carbonate.

e Diethyl carbonate.

f Dimethyl carbonate.

Initially, we studied the acetylation of mesitylene (1a) with acetic anhydride (2a) in the absence of a catalyst to confirm that the reaction predominantly requires catalysis (Table 1, Entry 1).

**Scheme 1:**
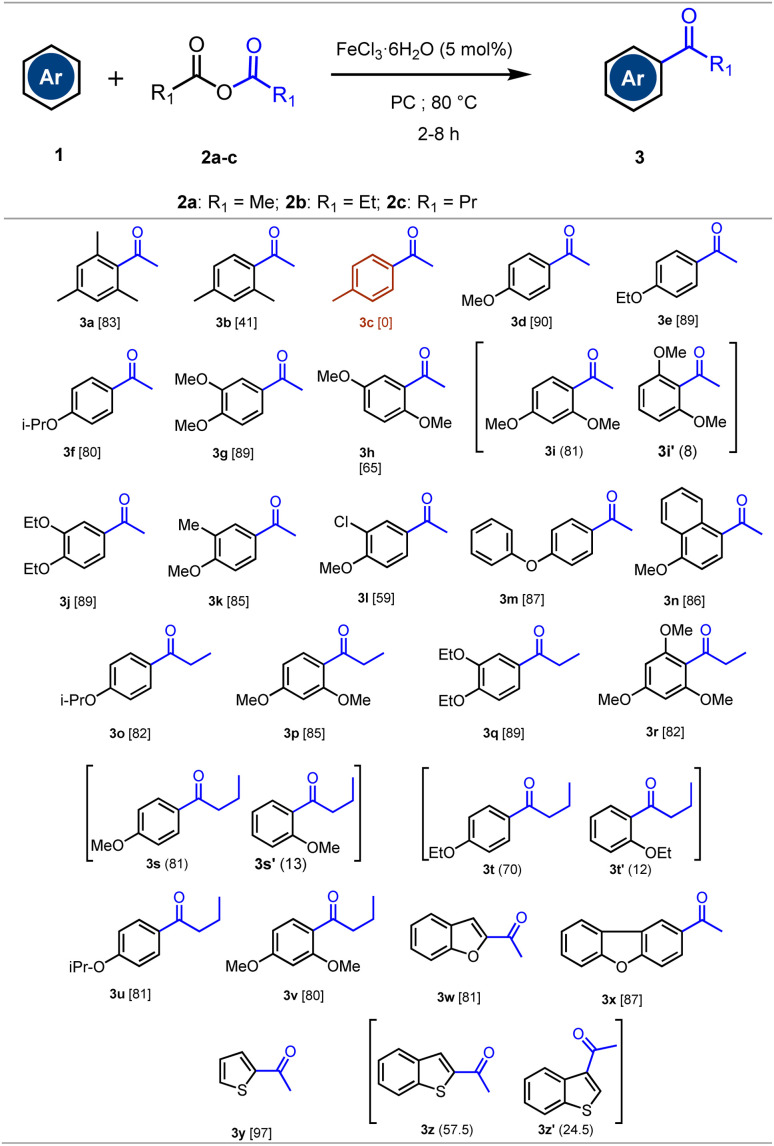
Synthesis of aromatic ketones by acylation of arenes with acid anhydrides under our optimized conditions.^*a*^ (a) Reaction conditions: 1 (1 mmol), 2 (2 mmol), FeCl_3_·6H_2_O (5 mol%), PC (1 mL). The mixture was stirred in a 8 mL pressure tube for 2–8 h at 80 °C.

Then, we tested the efficiency of AlCl_3_, ZnCl_2_ and CuCl_2_ at 5 mol% ratio (Table 1, Entries 2–5). These experiments revealed that, under the reaction conditions (solvent: PC; *T* = 80 °C) the conversion of the substrates into the corresponding ketone did not exceed 31%. The utilization of certain iron(iii) salts, such as iron trifluoroacetate, iron acetoacetate, and iron sulfate, proved unsatisfactory, as they did not significantly promote product formation (Table 1, Entries 6–8). In contrast, promising results were achieved using 5 mol% of iron(iii) chloride as a catalyst at 80 °C in propylene carbonate (PC) as the solvent (Table 1, Entries 9 and 10). Subsequently, we further explored these promising results by evaluating the catalytic activity of iron(iii) chloride at different concentrations and under diverse conditions. Indeed, it was found that: (i) at 5 mol% *ratio*, the catalytic efficiency of FeCl_3_·6H_2_O was slightly better than that of anhydrous FeCl_3_ as the conversions to the ketone were 95% and 88%, respectively, under the same reaction conditions (Table 1, Entries 9 and 10), This improvement can be attributed to the hydrated form FeCl_3_·6H_2_O, which often shows better catalytic activity than anhydrous FeCl_3_. Its improved solubility and modulation of Lewis acidity stabilize intermediates and create a more conducive environment for substrate activation, increasing conversion efficiency under the same conditions. (ii) The catalytic efficiency of FeCl_3_·6H_2_O at different ratios resulted in good to high conversions (82–95%). Surprisingly, 2 mol% of FeCl_3_·6H_2_O achieved 82% of conversion into the corresponding ketone (Table 1, Entry 11), and the highest catalytic activity was observed with 5 mol% of FeCl_3_·6H_2_O, which was the same as 10 mol% (Table 1, Entries 10 and 13). (iii) We have found that heating up to 80 °C is necessary to ensure the efficiency of the catalytic activity of the catalyst and to convert quantitatively the reactants into the corresponding ketone, since at room temperature; the conversion was 22% (Table 1, Entry 14). (iv) We also examined the influence of the solvent, revealing that it is more favourable under solvent-free conditions or in the presence of organic carbonates as solvents: PC (95%) > DEC (88%) > solvent-free (87%) > DMC (74%) (Table 1, Entries 10, 15–17), In contrast, using ethyl acetate, dichloromethane, or acetonitrile as solvents resulted in conversions of 47%, 38%, and 33%, respectively (Table 1, Entries 18–12). Therefore, the optimum conditions for the acetylation reaction of mesitylene with acetic anhydride are as follows: heating a mixture of mesitylene (1 mmol), acetic anhydride (2 mmol), and FeCl_3_·6H_2_O (5 mol%) at 80 °C for 8 h in PC (1 mL) as the solvent. These conditions lead to the formation of the corresponding ketone with the highest conversion (95%). Under the optimal conditions identified, we explored the scope and constraints of the reaction by subjecting various arenes to the acylation process as outlined in Scheme 1. This approach enabled the synthesis of a diverse range of aromatic ketones. The outcomes of these experiments are detailed in Scheme 1.

As indicated in Scheme 1, the reactivity of arenes with acid anhydrides depends on the activating groups attached to the aromatic ring. For instance, toluene did not undergo reaction with acetic anhydride under the given reaction conditions (3c: 0%). On the other hand, moderate reactivity was observed with *m*-xylene, yielding 41% of the corresponding ketone 3b. While, good to high reactivity was observed with more activated arenes such as trimethylbenzene, aromatic ethers and some aromatic heterocycles. In these cases, aromatic ketones were isolated with satisfactory yields (59–97%).

**Scheme 2:**
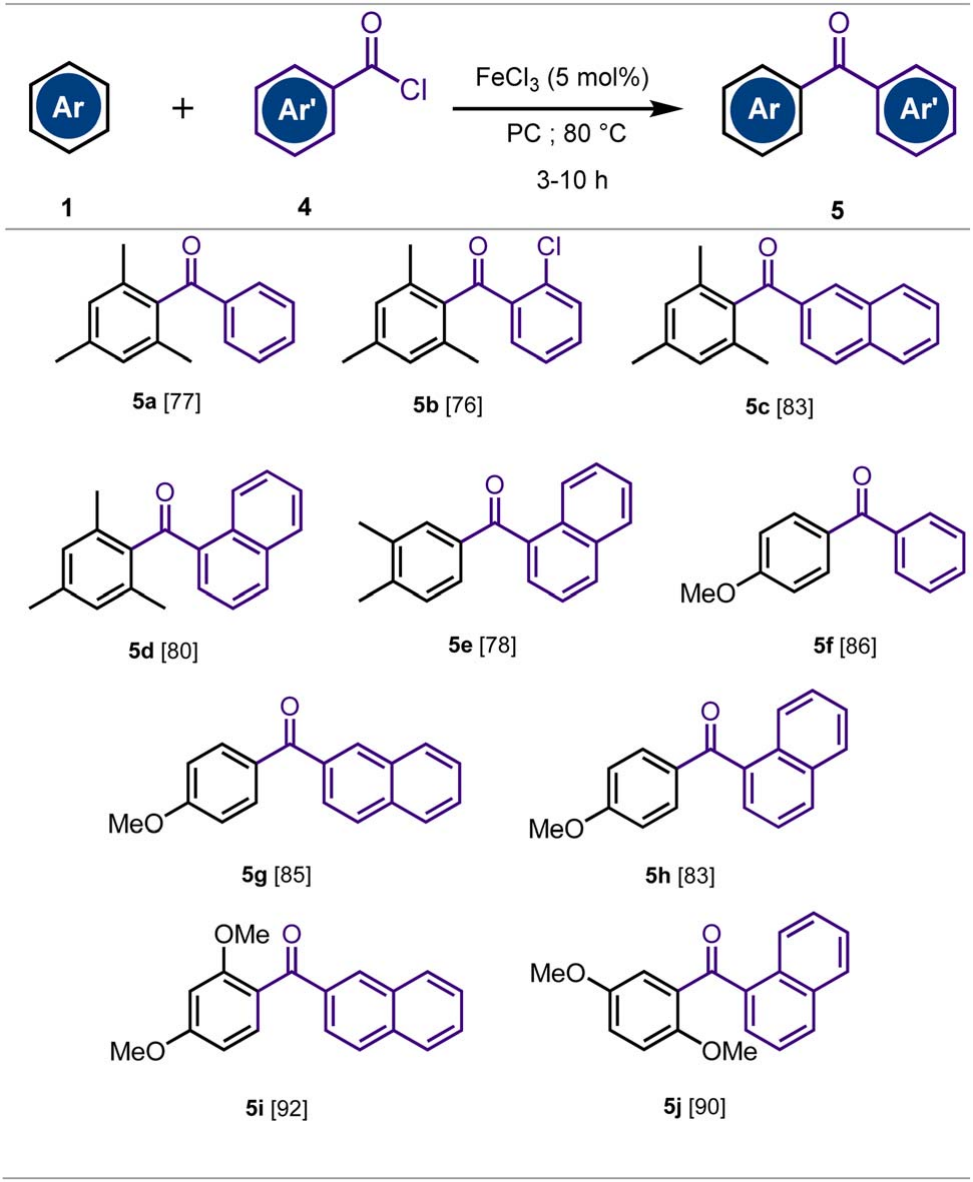
Acylation of activated arenes with aromatic acyl chlorides.^*a*^ (a) Reaction conditions: 1 (1.2 mmol), 4 (1 mmol), FeCl_3_ (5 mol%), PC (1 mL). The mixture was stirred in a 8 mL pressure tube for 3–10 h at 80 °C.

Encouraged by the interesting results obtained using the catalytic FeCl_3_·6H_2_O/PC system for the synthesis of aromatic ketones, and in order to broaden the scope of our method, we investigated the preparation of the synthetically useful diarylketones (Ar–C(O)–Ar′). We selected aromatic acyl chlorides as acylation agents instead of aryl acid anhydrides, as the latter produce aromatic carboxylic acids as waste, significantly reducing atom economy. On the other hand, due to the sensitivity of acyl chlorides to moisture, anhydrous FeCl_3_ was used as the catalyst and anhydrous propylene carbonate (PC) as the solvent. A variety of activated arenes was then reacted with aromatic acyl chlorides and the results are summarized in Scheme 2.

As illustrated in Scheme 2, the acylation of arenes with aromatic acid chlorides demonstrated the effectiveness of the method using 5 mol% of FeCl_3_ as the catalyst, and PC as the solvent at 80 °C. The reaction provided the aromatic ketones with good to high chemical yields (76–92%). Furthermore, under the reaction conditions, the acylation of anisole and 1,3-dimethoxybenzene with aromatic acyl chlorides afforded exclusively the *para*-regioisomers 5f–j. This is certainly due to the steric hindrance that disadvantages the substitution in the *ortho*-position under the reaction conditions.

**Scheme 3:**
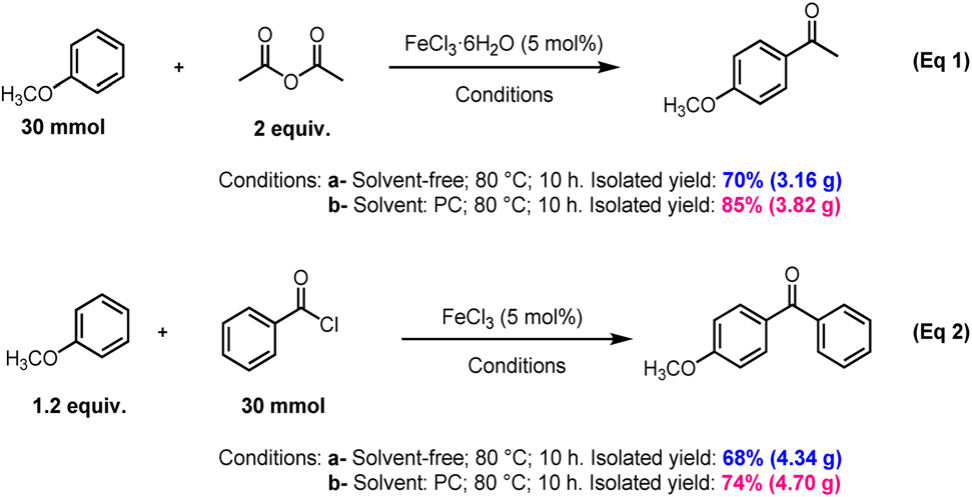
Acylation of anisole at 30 mmol scale.

Our new approach for acylation of arenes with acid anhydrides and acyl chlorides, in the presence of 5 mol% of iron(iii) chloride as catalyst and PC as solvent under relatively mild conditions, has shown good efficiency and broad scope, thus allowing the preparation of a wide range of aromatic ketones. We believe that the method described in this work represents significant advances in terms of economy, efficiency and environmental friendliness, and appears suitable for large-scale and industrial applications for the following raisons: (i) Iron(iii) chloride is an available, less-toxic and inexpensive catalyst that showed high catalytic activity at 5 mol% *ratio*. Consequently, this allows the avoidance of significant amounts of metal-based waste, which would otherwise be generated when the Lewis acid catalyst is used in stoichiometric or excess quantities. (ii) We emphasize that PC was the solvent that provided the best results in the acylation of arenes (Table 1). Recent studies from our laboratory, both published^28,30^ and unpublished, have demonstrated the beneficial role of organic carbonate solvents, particularly propylene carbonate (PC), in enhancing reaction efficiency. PC has gained considerable attention as a green solvent due to its low toxicity, high biodegradability, low volatility, excellent stability under reaction conditions, and production from renewable sources such as CO_2_. These properties make PC a sustainable and environmentally friendly solvent choice that aligns well with the principles of green chemistry, justifying its use in the present study.^29–33^

(iii) As illustrated in Scheme 3, we have found that increasing the scale of the reaction from 1 mmol to 30 mmol gave satisfactory results. Indeed, when 30 mmol of anisole are acetylated with acetic anhydride, the reaction yielded 70% and 85% of the corresponding ketone under solvent-free and in the presence of PC respectively (Scheme 3, eqn (1)).

Similarly, benzoylation of anisole at 30 mmol scale with benzoyl chloride provided 68% and 74% of the corresponding ketone under solvent-free and in the presence of PC respectively (Scheme 3, eqn (2)).

After studying the scope of our iron-catalyzed Friedel–Crafts acylation of arenes, our attention turned to investigating why the reaction requires only 5 mol% of iron, whereas previous methods utilized stoichiometric amounts of metals or more. To address this, we explored the interaction of FeCl_3_ with benzoyl chloride and the acylation Friedel–Crafts product, benzophenone, in benzene under reaction conditions (80 °C). Specifically, we examined whether the exchange between benzophenone and benzoyl chloride is both thermodynamically and kinetically feasible.

Particularly, concerns arose regarding potential catalyst poisoning by benzophenone ligands, due to their coordinating ability and steric bulk. This hypothesis is supported by crystal structures reported by Devery *et al.*^34^ Under conditions of excess benzophenone, a cationic pentacoordinate complex featuring two chloride ligands and three ketone ligands arranged in a trigonal bipyramidal geometry is expected to form.

Our computational approach followed the methodology proposed by Zimmerman and Devery,^35^ which is well-suited for aromatic systems and considering π-stacking interactions.

Upon conversion of benzoyl chloride into benzophenone, the catalyst is known to be potentially poisoned by benzophenone ligands. Consequently, the coordination sphere of the catalyst can be considered as primarily composed of chloride and benzophenone ligands. Actually, coordination of three benzophenone molecules to iron results in the displacement of three EC molecules and a gain of −23 kcal mol^−1^ in the gas phase. Accordingly, after saturation of the iron coordination sphere by benzophenone, the question is whether the exchange of benzophenone for benzoyl chloride is thermodynamically and kinetically feasible under the reaction conditions (benzene as solvent, 80 °C).

**Fig. 1:**
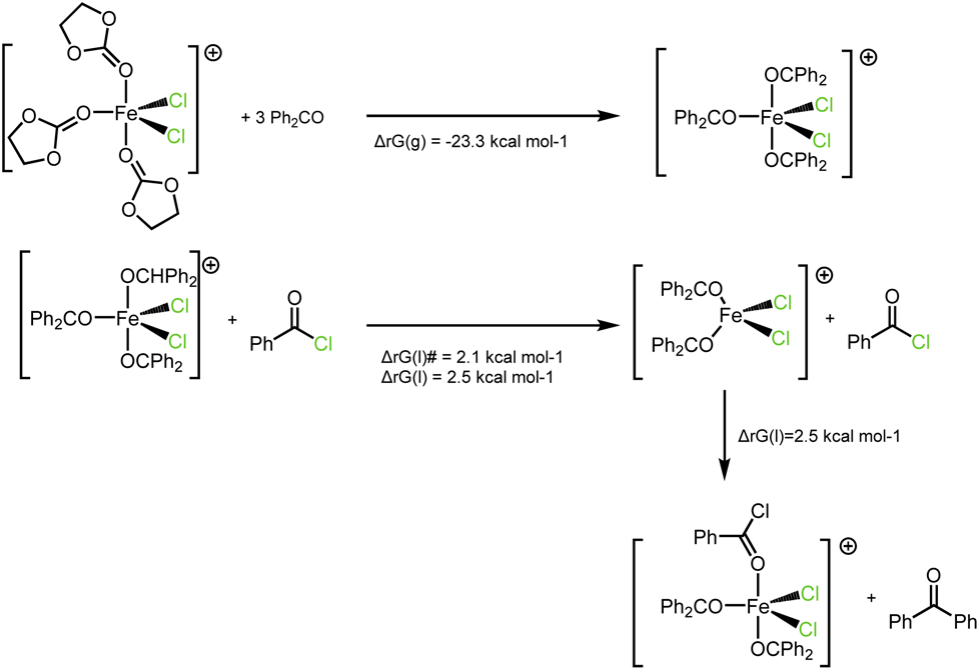
Thermodynamic values of possible benzophenone to benzoyl chloride exchanges, showing a rather flat potential surface.

According to our calculations, exchanging one benzophenone ligand on pentacoordinated, [(Ph_2_CO)_3_FeCl_2_]^+^ by a benzoyl chloride ligand is slightly endergonic (2.5 kcal mol^−1^ in liquid phase, within the accuracy limit of our method). Benzoyl chloride is found to coordinate to iron through the oxygen atom and in axial position. In fact, all other coordination modes of benzoyl chloride (equatorial position and/or chloride coordination) lead to spontaneous dissociation of the benzoyl chloride. All the resulting tetracoordinated geometries are however close in energy compared to the pentacoordinated, axial, κ1O benzoyl chloride complex (within 5.5 kcal mol^−1^ higher or lower free energy in liquid phase). From a kinetic point of view, dissociation of one benzophenone from the FeCl_2_ plane is almost costless (2.1 kcal mol^−1^). No transition state could be found for dissociation of an axial benzophenone. The barrier can be estimated by a scan of energy on the Fe–O bond distance, which reach a plateau of 9.8 kcal mol^−1^ at 4.66 Å in the gas phase. As a conclusion, the energy surface corresponding to a dissociation of benzophenone ligand and coordination of the benzoyl chloride substrate is relatively flat, and this transformation is thermodynamically feasible in the reaction condition. Altogether, according to our model, the catalyst remains accessible to the substrate at high conversion rates, with very low values of kinetic barriers and at low thermodynamic expenses (Fig. 1).

In benzene at 80 °C, it seems feasible kinetically for benzophenone to replace benzoyl chloride as a ligand. Although the thermodynamic preferences slightly favor benzophenone coordination over benzoyl chloride, the substantial energy gain from the subsequent Friedel–Crafts reaction can significantly bias this equilibrium.^36^

## Conclusion

In summary, we have developed an environmentally friendly method for the acylation of activated arenes using both acid anhydrides and aromatic acyl chlorides, enabling the synthesis of a diverse range of aromatic ketones with consistently good to high yields. Our method employs iron(iii) chloride as a readily available, low-toxicity, and cost-effective Lewis acid catalyst, with propylene carbonate serving as a green solvent. Operating under relatively mild conditions, the iron(iii) chloride catalyst remains effective at a low loading of 5 mol% without compromising catalytic activity. This approach addresses a major limitation of traditional Friedel–Crafts acylation and represents a significant advancement in green chemistry. We believe this green and economical method is well-suited for industrial scale preparations.

Preliminary density functional theory (DFT) calculations were conducted to elucidate the catalytic role of iron in the reaction mechanism. This approach complements the recent photocatalyzed reaction reported by Studer *et al.*,^15^ where a distinct acylation regioisomer was achieved through the integration of NHC catalysis with photoredox catalysis.

## Author contributions

S. E. C., M. C. and R. R. performed the synthetic experiments; M. F. B. performed the DFT calculation; S. L. and J. K designed and supervised the project. The manuscript was written through contributions of all authors. All authors have given approval to the final version of the manuscript.

## Conflicts of interest

The authors declare no competing financial interest.

## Supplementary Material

RA-015-D5RA03638K-s001

## Data Availability

The data supporting this article have been included as part of the SI, including complete experimental details and characterization data for the prepared compounds (General information, General procedure for FC acylation, NMR spectroscopic data, NMR spectra, DFT calculation). Supplementary information available: Experimental details, spectral data, copies of ^1^H and ^13^C NMR spectra and DFT calculation. See DOI: https://doi.org/10.1039/d5ra03638k.
