# Supplementary material for: Eco-friendly and efficient Friedel–Crafts acylation of activated arenes catalyzed with low-loaded ferric chloride in propylene carbonate as the solvent: scope and mechanistic insights
Source: RSC Adv. 2025 Sep 1;15(38):31088–94. doi: 10.1039/d5ra03638k (PMC12400303; doi:10.1039/d5ra03638k)

Supplementary Information (SI) for RSC Advances.
This journal is © The Royal Society of Chemistry 2025

Supporting Information

Eco-friendly and Efficient Friedel–Crafts Acylation of Activated arenes Catalyzed with Low Loaded Ferric Chloride in propylene carbonate as the solvent: scope and mechanistic insights

Saif Eddine Cherif,[a,b] *Mayssa Chehaibi,*[a] *Rafif Raddadi,*[a] *Marie Fustier-Boutignon,*[b] *Sami Lakhdar,*[b]* *and Jamil Kraïem*[a]*

a-Laboratoire de Développement Chimique, Galénique et Pharmacologique des Médicaments, Faculté de Pharmacie de Monastir, Université de Monastir, 5000Monastir, Tunisia.

b- CNRS, Université Paul Sabatier, Laboratoire Hétérochimie Fondamentale et Appliquée (LHFA, UMR5069), 118 Route de Narbonne, 31062 Cedex 09 Toulouse, France.

sami.lakhdar@univ-tlse3.fr

jamil.kraiem@fphm.u-monastir.tn

Table of contents

Content **Pages**

1-General Information……………………………………………………………….……2

2-General procedure for synthesis………………….…………………………….….…2

General procedure for Friedel-Crafts reaction (using alkanoic anhydrides)………..…2

General procedure for Friedel-Crafts reaction (using acid chlorides)…………….......12

4-Compuational details…………………………………………………………………..16

5-References…………………………………………………………………..……..21

6-Copies of NMR spectra……………………………………………………….……..23

General Information:

All chemicals were purchased from Sigma-Aldrich and used without further purifications for synthesis. ^{1}H NMR (300MHz); and ^{13}C proton decoupling (75 MHz)· spectra were recorded on an Agilent Direct Drive spectrometer (Agilent Technologies, Santa Clara) in $CDCl_3$. The chemical shifts are given in ppm and refer to $CDCl_3$ (H = 7.26, C = 77.16) as internal standard. Coupling constants *J* are measured in Hz. Splitting patterns are designed as follows: s = singlet; d = doublet; t = triplet; q = quartet, dd = doublet of doublet; m = multiplet. Thin-layer chromatography (TLC) was performed on Merck 60F-254 precoated silica gel sheets (0.25 mm). Propylene carbonate (Reagent plus, 99%), Iron chloride and Iron chloride hexahydrate were purchased from Sigma-Aldrich. All chemicals obtained from commercial suppliers were used without further purification. Anhydrous solvents were prepared according to the standard methods. The calculations were performed using HPC resources from CALMIP supercomputer center (Grant 2023-p16018).

General procedure for synthesis

General procedure for Friedel-Crafts reaction (using acetic anhydride).

A 8 mL pressure tube was charged with aromatic compound **(1 eq, 1 mmol),** propylene carbonate (PC) **(1 mL),** Iron(III) Chloride Hexahydrate **(5 mol %)** and the alkanoic anhydride **(2 eq, 2 mmol)**. The reaction mixture was stirred at 80℃ for specified time (2-8h). After completion of the reaction (monitored by TLC), the mixture was washed with saturated solution of Na_2CO_3 (5 mL). The organic layer was separated from the mixture by centrifugation, washed twice with 2 mL of water (to remove traces of PC and the catalyst), and purified by flash chromatography on silica gel (cyclohexane/Ethyl acetate 95/5) to provide the pure product. All aromatic ketones prepared in this work are described in the literature.

Compound (3a)[1]:

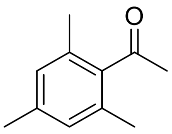

1,3,5-trimethylbenzene (140 µL, 1.0 mmol); acetic anhydride (190 µL, 2 mmol), Iron(III) Chloride Hexahydrate (13.5 mg, 0.05 mmol, 5 mol %);

Purification (colorless oil, 134 mg, 0.83 mmol, 83 %). ^{1}H NMR (300 MHz, $CDCl_3$) (ppm) 6.84 (s, 2H), 2.45 (s, 3H), 2.28 (s, 3H) 2.22 (s, 6H).^{13}C NMR (75 MHz, $CDCl_3$) (ppm) 208.4, 140.1, 138.4, 132.4, 128.6, 32.3, 21.1, 19.2.

Compound (3b)[2]:

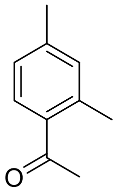

Metaxylene (123 µL, 1.0 mmol); acetic anhydride (190 µL, 2 mmol), Iron(III) Chloride Hexahydrate (13.5 mg, 0.05 mmol, 5 mol %);

Purification (yellow oil, 61 mg, 0.41 mmol, 41%). ^{1}H NMR (300 MHz, $CDCl_3$) δ (ppm) 7.64 (d, J = 8.5 Hz, 1H), 7.09 – 7.05 (m, 2H), 2.56 (s, 3H), 2.52 (s, 3H), 2.35 (s, 3H). ^{13}C NMR (75 MHz, $CDCl_3$) δ (ppm) 200.9, 142.1, 138.9, 134.6, 132.9, 130.0, 126.3, 29.3, 21.8, 21.3.

Compound (3d)[1]:

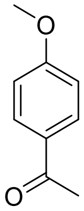

anisole (110 µL, 1.0 mmol); acetic anhydride (190 µL, 2 mmol), Iron(III) Chloride Hexahydrate (13.5 mg, 0.05 mmol, 5 mol %);

Purification (yellow oil, 135 mg, 0.9 mmol, 90 %). ^{1}H NMR (300 MHz, $CDCl_3$) □ (ppm) 7.91 (d, J = 8.9 Hz, 2H), 6.91 (d, J = 8.9 Hz, 3H), 3.84 (s, 3H) 2.53 (s, 3H).^{13}C NMR (75 MHz, $CDCl_3$) δ (ppm) 196.8, 161.6, 130.7, 130.4, 113.8, 55.5, 26.4.

Compound (3e)[1]:

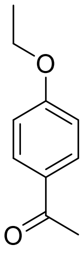 Ethoxybenzene (127 µL, 1.0 mmol); acetic anhydride (190 µL, 2 mmol), Iron(III) Chloride Hexahydrate (13.5 mg, 0.05 mmol, 5 mol %);

Purification (Yellow oil, 146 mg, 0.89 mmol, 89 %). ^{1}H NMR (300 MHz, $CDCl_3$) δ (ppm) 7.90 (d, *J* = 8.9 Hz, 2H), 6.89 (d, *J* = 8,9 Hz, 1H), 4.08 (q, *J* = 7.0 Hz, 2H), 2.52 (s, 3H), 1.41 (t, *J* = 7.0 Hz, 3H). ^{13}C NMR (75 MHz, $CDCl_3$) δ (ppm) 196.7, 163.0, 130.6, 130.4, 114.3, 63.8, 26.3, 14.7.

Compound (3f)[3]:

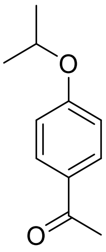 Isopropoxybenzene (151 µL, 1.0 mmol); acetic anhydride (190 µL, 2 mmol), Iron(III) Chloride Hexahydrate (13.5 mg, 0.05 mmol, 5 mol %);

Purification (Yellow solid, 143 mg, 0.80 mmol, 80 %). Mp: 38-39 °C. ^{1}H NMR (300 MHz, $CDCl_3$) δ (ppm) 7.90 (d, *J* = 8.9 Hz, 2H), 6.89 (d, *J* = 8,9 Hz, 1H), 4.63 (hept, *J* = 6.1 Hz, 1H), 2.53 (s, 3H), 1.36 (d, *J* = 6.1 Hz, 6H). ^{13}C NMR (75 MHz, $CDCl_3$) δ (ppm) 196.7, 162.2, 130.7, 130.2, 115.3, 70.3, 26.3, 22.0.

Compound (3g)[1]:

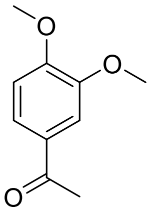 1,2-dimethoxybenzene (128 µL, 1.0 mmol); acetic anhydride (190 µL, 2 mmol), Iron(III) Chloride Hexahydrate (13.5 mg, 0.05 mmol, 5 mol %);

Purification (White solid, 160 mg, 0.89 mmol, 89 %). Mp: 50-52 °C. ^{1}H NMR (300 MHz, $CDCl_3$) δ (ppm) 7.55 – 7.47 (m, 2H), 6.84 (d, *J* = 8.6 Hz, 1H), 3.90 (s, 3H), 3.89 (s, 3H) 2.52 (s, 3H).^{13}C NMR (75 MHz, $CDCl_3$) δ (ppm) 196.9, 153.2, 148.9, 130.4, 123.3, 109.9, 109.9, 56.1, 56.0, 26.2.

Compound (3h)[4]:

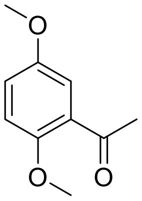

1,4-dimethoxybenzene (158 µL, 1.0 mmol); acetic anhydride (190 µL, 2 mmol), Iron(III) Chloride Hexahydrate (13.5 mg, 0.05 mmol, 5 mol %);

Purification (Yellow oil, 118 mg, 0.65 mmol, 65 %). ^{1}H NMR (300 MHz, $CDCl_3$) δ (ppm) 7.17 (d, *J* = 3.2 Hz, 1H), 6.91 (dd, *J* = 8,8, 3.2 Hz, 1H), 6.79 (d, *J* = 9.1 Hz, 1H), 3.75 (s, 3H), 3.67 (s, 3H), 2.49 (s, 3H).^{13}C NMR (75 MHz, $CDCl_3$) δ (ppm) 199.3, 153.6, 128.7, 120.3, 114.1, 113.4, 56.1, 55.9, 31.8.

Compound (3i)[2]:

1,3-dimethoxybenzene (131 µL, 1.0 mmol); acetic anhydride (190 µL, 2 mmol), Iron(III) Chloride Hexahydrate (13.5 mg, 0.05 mmol, 5 mol %);

Purification (White solid, 162 mg, 0.90 mmol, 89 %).

- **Product 3i : (81%)**

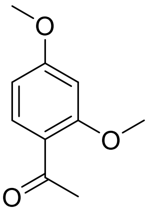

^{1}H NMR (300 MHz, $CDCl_3$) δ (ppm) 7.79 (d, *J* = 8.8 Hz, 1H), 6.52 – 6.39 (m, 2H), 3.86 (s, 3H), 3.82 (s, 3H), 2.54 (s, 3H). ^{13}C NMR (75 MHz, $CDCl_3$) δ (ppm) 197.7, 164.6, 161.2, 132.7, 121.4, 105.3, 98.5, 55.5, 55.5, 31.8.

- **Product 3i' : (8%)**

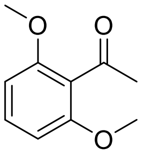

^{1}H NMR (300 MHz, $CDCl_3$) δ (ppm) 7.26 – 7.19 (m, 1H), 6.53 (d, J = 8.8 Hz, 2H), 3.77 (s, 6H), 2.44 (s, 3H). ^{13}C NMR (75 MHz, $CDCl_3$) δ (ppm) 201.8, 156.9, 130.7, 121.4, 104.2, 55.9, 32.3.

Compound (3j)[2]:

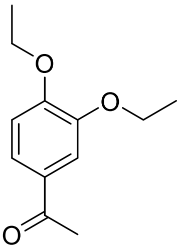

1,2-diethoxybenzene (166 mg, 1.0 mmol); acetic anhydride (190 µL, 2 mmol), Iron(III) Chloride Hexahydrate (13.5 mg, 0.05 mmol, 5 mol %);

Purification (white solid, 185 mg, 0.89 mmol, 89%). Mp: 52-54 °C. ^{1}H NMR (300 MHz, $CDCl_3$) δ (ppm) 7.56 – 7.52 (m, 2H), 6.87 (d, *J* = 8.3 Hz, 1H), 4.16 (q, J = 7.0 Hz, 2H), 4.15 (q, *J* = 7.0 Hz, 2H), 2.55 (s, 3H), 1.49 (t, *J* = 7.0 Hz, 3H), 1.47 (t, *J* = 7.0 Hz, 3H) . ^{13}C NMR (75 MHz, $CDCl_3$) δ (ppm) 197.0, 153.2, 148.6, 130.4, 123.3, 112.1, 111.4, 64.7, 64.6, 26.4, 14.8, 14.8.

Compound (3k)[5]:

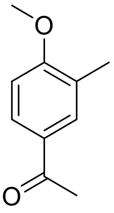

2-methylanisole (124 µL, 1.0 mmol); acetic anhydride (190 µL, 2 mmol), Iron(III) Chloride Hexahydrate (13.5 mg, 0.05 mmol, 5 mol %);

Purification (Yellow oil, 140 mg, 0.85 mmol, 85 %). ^{1}H NMR (300 MHz, $CDCl_3$) □ (ppm) 7.83 – 7.76 (m, 2H), 6.84 (d, *J* = 8.5 Hz, 1H), 3.89 (s, 3H) 2.54 (s, 3H), 2.25 (s, 3H).^{13}C NMR (75 MHz, $CDCl_3$) □ (ppm) 197.1, 162.0, 131.1, 130.2, 128.6, 127.6, 109.4, 56.7, 26.4, 16.3.

Compound (3l)[6]:

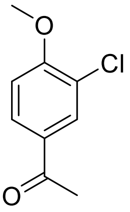

2-chloroanisole (127 µL, 1.0 mmol); acetic anhydride (190 µL, 2 mmol), Iron(III) Chloride Hexahydrate (13.5 mg, 0.05 mmol, 5 mol %);

Purification (Yellow solid, 109 mg, 0.59 mmol, 59 %). Mp: 70-71 °C. ^{1}H NMR (300 MHz, $CDCl_3$) δ (ppm) 7.97 (s, 1H), 7.86 (d, *J* = 6.5 Hz, 1H), 6.96 (d, *J* = 8.7 Hz, 1H), 3.96 (s, 3H), 2.54 (s, 3H).^{13}C NMR (75 MHz, $CDCl_3$) δ (ppm) 195.8, 158.9, 130.9, 130.8, 128.9, 122.9, 111.4, 56.5, 26.4.

Compound (3m)[7]:

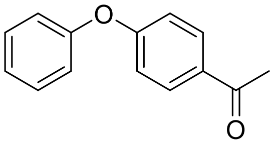

Diphenyl ether (158 µL, 1.0 mmol); acetic anhydride (190 µL, 2 mmol), Iron(III) Chloride Hexahydrate (13.5 mg, 0.05 mmol, 5 mol %);

Purification (Yellow solid, 185 mg, 0.87 mmol, 87 %). Mp/ 48-50 °C. ^{1}H NMR (300 MHz, $CDCl_3$) δ (ppm) 7.93 (d, *J* = 8.8 Hz, 2H), 7.39 (t, *J* = 7.8 Hz, 2H), 7.20 (t, *J* = 7.4 Hz, 1H), 7.06 (d, *J* = 7.6 Hz, 2H), 6.99 (d, *J* = 8.9 Hz, 2H), 2.56 (s, 3H). ^{13}C NMR (75 MHz, $CDCl_3$) δ (ppm) 196.8, 162.0, 155.5, 132.0, 130.7, 130.1, 124.7, 120.2, 117.3, 26.5.

Compound (3n)[8]:

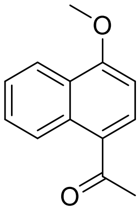

1-methoxynaphtalene (145 µL, 1.0 mmol); acetic anhydride (190 µL, 2 mmol), Iron(III) Chloride Hexahydrate (13.5 mg, 0.05 mmol, 5 mol %);

Purification (White solid, 172 mg, 0.86 mmol, 86 %). Mp: 73-74 °C. ^{1}H NMR (300 MHz, $CDCl_3$) δ (ppm) 9.04 (dd, *J* = 8.7, 0.6 Hz, 1H), 8.31 (dd, *J* = 8.4, 0.9 Hz, 1H), 7.97 (d, *J* = 8.2 Hz, 1H), 7.63 (ddd, *J* = 8.6, 6.8, 1.5 Hz, 1H), 7.51 (ddd, *J* = 8.2, 6.8, 1.3 Hz, 1H), 6.72 (d, *J* = 8.2 Hz, 1H), 4.02 (s, 3H), 2.69 (s, 3H). ^{13}C NMR (75 MHz, $CDCl_3$) δ (ppm) 200.0, 159.3, 132.1, 128.7, 127.2, 126.3, 125.8, 122.1, 102.1, 55.8, 29.3.

Compound (3o)[9]:

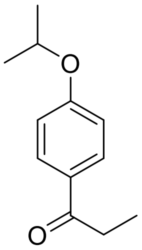

Isopropoxybenzene (151 µL, 1.0 mmol); propanoic anhydride (256 µL, 2 mmol), Iron(III) Chloride Hexahydrate (13.5 mg, 0.05 mmol, 5 mol %);

Purification (Yellow solid, 158 mg, 0.82 mmol, 82 %). Mp: 52-53 °C. ^{1}H NMR (300 MHz, $CDCl_3$) □ (ppm) 7.92 (d, *J* = 9.0 Hz, 2H), 6.89 (d, *J* = 8,9 Hz, 2H), 4.63 (hept, *J* = 6.1 Hz, 1H), 2.93 (q, *J* = 7.3 Hz, 2H), 1.35 (d, *J* = 6.1 Hz, 6H), 1.20 (t, *J* = 7.3 Hz, 3H) . ^{13}C NMR (75 MHz, $CDCl_3$) □ (ppm) 199.5, 161.8, 130.3, 129.6, 115.1, 70.0, 31.4, 21.9, 8.5.

Compound (3p)[10]:

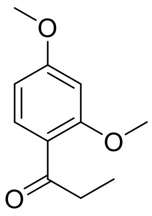

1,3-dimethoxybenzene (131 µL, 1.0 mmol); butanoic anhydride (315 µL, 2 mmol), Iron(III) Chloride Hexahydrate (13.5 mg, 0.05 mmol, 5 mol %);

Purification (White solid, 165 mg, 0.85 mmol, 85 %). Mp: 66-68 °C. ^{1}H NMR (300 MHz, $CDCl_3$) δ (ppm) 7.81 (d, *J* = 8.7 Hz, 1H), 6.52 (dd, *J* = 8.7, 2.3 Hz, 1H), 6.45 (d, *J* = 2.3 Hz, 1H), 3.88 (s, 3H), 3.85 (s, 3H), 2.96 (q, J = 7.8 Hz, 2H), 1.15 (t, *J* = 7.3 Hz, 3H). ^{13}C NMR (75 MHz, $CDCl_3$) δ (ppm) 201.3, 164.3, 160.9, 132.7, 121.3, 105.1, 98.5, 55.6, 55.6, 37.0, 8.7.

Compound (3q)[11]:

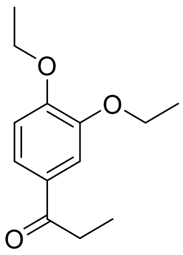

1,2-diethoxybenzene (166 mg, 1.0 mmol); propanoic anhydride (256 µL, 2 mmol), Iron(III) Chloride Hexahydrate (13.5 mg, 0.05 mmol, 5 mol %);

Purification (colorless oil, 198 mg, 0.89 mmol, 89%). ^{1}H NMR (300 MHz, $CDCl_3$) δ (ppm) 7.57 – 7.53 (m, 2H), 6.87 (d, *J* = 8.2 Hz, 1H), 4.16 (q, J = 7.0 Hz, 2H), 4.15 (q, *J* = 7.0 Hz, 2H), 2.95 (q, *J* = 7.3 Hz, 2H), 1.48 (t, *J* = 7.0 Hz, 3H), 1.46 (t, *J* = 7.0 Hz, 3H), 1.21 (t, *J* = 7.3 Hz, 3H). ^{13}C NMR (75 MHz, $CDCl_3$) δ (ppm) 199.7,153.0, 148.6, 130.1, 122.6, 112.2, 111.5, 64.7, 64.6, 31.4, 14.9, 14.8, 8.7.

Compound (3r)[12]:

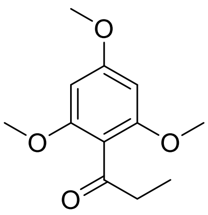

1,3,5-trimethoxybenzene (168 mg, 1.0 mmol); propanoic anhydride (256 µL, 2 mmol), Iron(III) Chloride Hexahydrate (13.5 mg, 0.05 mmol, 5 mol %);

Purification (White solid, 184 mg, 0.82 mmol, 82 %). Mp: 94-95 °C. ^{1}H NMR (300 MHz, $CDCl_3$) □ (ppm) 6.09 (s, 2H), 3.81 (s, 3H), 3.76 (s, 6H), 2.74 (q, *J* = 7.3 Hz, 2H), 1.12

(t, *J* = 7.3 Hz, 3H). ^{13}C NMR (75 MHz, $CDCl_3$) □ (ppm) 205.5, 162.2, 158.1, 113.8, 90.7, 55.9, 55.5, 38.2, 8.1.

Compound (3s)[13]:

anisole (110 µL, 1.0 mmol); butanoic anhydride (315 µL, 2 mmol), Iron(III) Chloride Hexahydrate (13.5 mg, 0.05 mmol, 5 mol %);

Purification (Yellow oil, 168 mg, 0.94 mmol, 94 %).

- **Product 3s : (81%)**

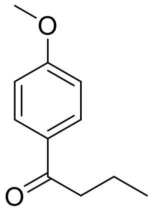

^{1}H NMR (300 MHz, $CDCl_3$) δ (ppm) 7.94 (d, *J* = 8.9 Hz, 2H), 6.93 (d, *J* = 8.9 Hz, 2H), 3.87 (s, 3H), 2.89 (t, *J* = 7.5 Hz, 2H), 1.76 (h, *J* = 7.4 Hz, 2H), 1.00 (t, *J* = 7.4 Hz, 3H). ^{13}C NMR (75 MHz, $CDCl_3$) δ (ppm) 199.2, 163.4, 131.2, 130.4, 113.8, 55.6, 40.4, 18.2, 14.1.

- **Product 3s': (13%)**

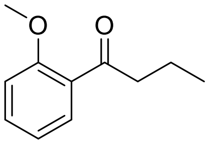

^{1}H NMR (300 MHz, $CDCl_3$) δ (ppm) 7.99 – 7.92 (m, 2H), 6.96 – 6.90 (m, 2H), 3.87 (s, 3H), 1.52 (h, *J* = 7.4 Hz, 2H), 0.92 (t, *J* = 7.4 Hz, 3H), 0.81 (t, *J* = 7.4 Hz, 3H). ^{13}C NMR (75 MHz, $CDCl_3$) δ (ppm) 208,8, 130.4, 114.1, 55.7, 42.3, 17.0, 13.6.

Compound (3t)[13]:

Ethoxybenzene (127 µL, 1.0 mmol); butanoic anhydride (315 µL, 2 mmol), Iron(III) Chloride Hexahydrate (13.5 mg, 0.05 mmol, 5 mol %);

Purification (white solid, 158 mg, 0.82 mmol, 82 %).

- **Product 3t: (70%)**

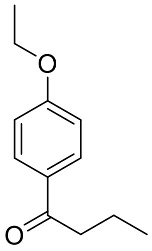 ^{1}H NMR (300 MHz, $CDCl_3$) δ (ppm) 7.93 (d, *J* = 8.9 Hz, 2H), 6.91 (d, *J* = 8.9 Hz, 2H), 4.09 (q, *J* = 7.0 Hz, 2H), 2.88 (t, *J* = 7.3 Hz, 2H), 1.75 (h, *J* = 7.4 Hz, 2H), 1.43 (t, *J* = 7.0 Hz, 3H), 0.99 (t, *J* = 7.4 Hz, 2H). ^{13}C NMR (75 MHz, $CDCl_3$) δ (ppm) 199.2, 162.9, 131.2, 130.4, 114.2, 63.8, 40.3, 18.2, 14.8, 14.1.

- **Product 3t': (12%)**

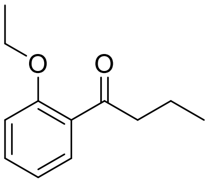 ^{1}H NMR (300 MHz, $CDCl_3$) δ (ppm) 7.97 – 7.90 (m, 2H), 6.94 – 6.88 (m, 2H), 4.09 (q, *J* = 7.0 Hz, 2H), 2.88 (t, *J* = 7.3 Hz, 2H), 1.52 (h, *J* = 7.4 Hz, 2H), 0.92 (t, *J* = 7.0 Hz, 3H), 0.81 (t, *J* = 7.4 Hz, 2H). ^{13}C NMR (75 MHz, $CDCl_3$) δ (ppm) 206.9, 195.0, 163.5, 130.2, 129.7, 114.5, 64.7, 42.3, 17.0, 13.7, 12.4.

Compound (3u)[15]:

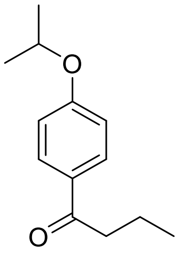 Isopropoxybenzene (151 µL, 1.0 mmol); butanoic anhydride (315 µL, 2 mmol), Iron(III) Chloride Hexahydrate (13.5 mg, 0.05 mmol, 5 mol %);

Purification (colorless oil, 167 mg, 0.81 mmol, 81 %). ^{1}H NMR (300 MHz, $CDCl_3$) δ (ppm) 7.92 (d, *J* = 8.9 Hz, 2H), 6.89 (d, *J* = 8,9 Hz, 2H), 4.64 (hept, *J* = 6.0 Hz, 1H), 2.88 (t, *J* = 7.7 Hz, 2H), 1.75 (h, *J* = 7.4 Hz, 2H), 1.36 (d, *J* = 6.1 Hz, 6H), 0.99 (t, *J* = 7.4 Hz, 3H) . ^{13}C NMR (75 MHz, $CDCl_3$) δ (ppm) 199.2, 161.9, 130.5, 129.9, 115.2, 70.2, 40.3, 22.1, 18.2, 14.1.

Compound (3v)[16]:

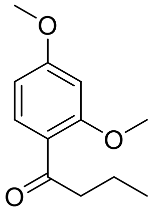 1,3-dimethoxybenzene (131 µL, 1.0 mmol); butanoic anhydride (315 µL, 2 mmol), Iron(III) Chloride Hexahydrate (13.5 mg, 0.05 mmol, 5 mol %);

Purification (colorless oil, 167 mg, 0.80 mmol, 80 %). ^{1}H NMR (300 MHz, $CDCl_3$) □ (ppm) 7.77 (d, *J* = 8.7 Hz, 1H), 6.51 (dd, *J* = 8.7, 2.3 Hz, 1H), 6.44 (d, *J* = 2.3 Hz, 1H), 3.87 (s, 3H), 3.84 (s, 3H), 2.9 (t, J = 7.8 Hz, 2H), 1.68 (h, *J* = 7.4 Hz, 2H), 0.95 (t, *J* = 7.4 Hz, 3H). ^{13}C NMR (75 MHz, $CDCl_3$) □ (ppm) 200.9, 164.3, 160.7, 132.7, 121.6, 105.1, 98.5, 55.6, 55.6, 45.7, 18.1, 14.1.

Compound (3w)[12]:

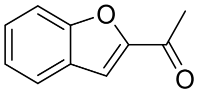 2,3-benzofuran (110 µL, 1.0 mmol); acetic anhydride (190 µL, 2 mmol), Iron(III) Chloride Hexahydrate (13.5 mg, 0.05 mmol, 5 mol %);

Purification (White solid, 130 mg, 0.81 mmol, 81 %). Mp: 76-77 °C. ^{1}H NMR (300 MHz, $CDCl_3$) δ (ppm) 7.73 – 7.68 (m, 1H), 7.60 – 7.56 (m, 1H), 7.51 – 7.44 (m, 2H), 7.31 (ddd, *J* = 8.0, 7.1, 1.0 Hz, 1H), 2.61 (s, 3H). ^{13}C NMR (75 MHz, $CDCl_3$) δ (ppm) 188.8, 155.8, 152.8, 128.4, 127.2, 124.0, 123.4, 113.2, 112.6, 26.6.

Compound (3x)[17]:

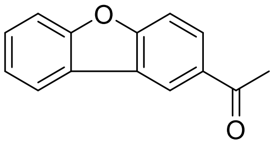 Dibenzofuran (168 mg, 1.0 mmol); acetic anhydride (190 µL, 2 mmol), Iron(III) Chloride Hexahydrate (13.5 mg, 0.05 mmol, 5 mol %);

Purification (Yellow solid, 183 mg, 0.87 mmol, 87 %). Mp: 67-69 °C. ^{1}H NMR (300 MHz, $CDCl_3$) δ (ppm) 8.57 (s, 1H), 8.09 (dd, *J* = 8.6, 1.9 Hz, 1H), 8.02 – 7.95 (m, 1H), 7.62 – 7.54 (m, 2H), 7.54 – 7.44 (m, 1H), 7.42 – 7.34 (m, 1H), 2.70 (s, 3H). ^{13}C NMR (75 MHz, $CDCl_3$) δ (ppm) 197.4, 159.0, 157.0, 132.6, 128.1, 124.7, 123.8, 123.5, 121.7, 121.1, 112.0, 111.7, 26.9.

Compound (3y)[5]:

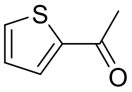 Thiophene (80 mg, 1.0 mmol); acetic anhydride (190 µL, 2 mmol), Iron(III) Chloride Hexahydrate (13.5 mg, 0.05 mmol, 5 mol %);

Purification (colorless oil, 122 mg, 0.97 mmol, 97 %). ^{1}H NMR (300 MHz, $CDCl_3$) δ (ppm) 7.67 (dd, *J* = 3.8, 1.1 Hz, 1H), 7.60 (dd, *J* = 5.0, 1.2 Hz, 1H), 7.09 (dd, *J* = 5.0, 3.8 Hz, 1H), 2.53 (s, 3H). ^{13}C NMR (75 MHz, $CDCl_3$) δ (ppm) 190.8, 144.6, 133.8, 132.5, 128.2, 26.9.

Compound (3z)[18]:

benzothiophene (117 µL, 1.0 mmol); acetic anhydride (190 µL, 2 mmol), Iron(III) Chloride Hexahydrate (13.5 mg, 0.05 mmol, 5 mol %).

Purification (White solid, 144 mg, 0.82 mmol, 82 %).

- **Product (3z) : (57.5%)**

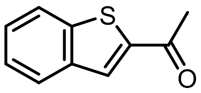

^{1}H NMR (300 MHz, $CDCl_3$) δ (ppm) 8.77 (d, *J* = 8.2 Hz, 1H), 8.23 (s, 1H), 7.83 (d, *J* = 7.7 Hz, 1H), 7.52 – 7.34 (m, 2H), 2.61 (s, 3H). ^{13}C NMR (75 MHz, $CDCl_3$) δ (ppm) 193.0, 139.8, 137.5, 136.4, 127.4, 125.8, 125.7, 125.4, 122.2, 28.1.

- **Product (3z') : (24.5%)**

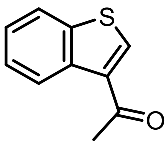 ^{1}H NMR (300 MHz, $CDCl_3$) δ (ppm) 7.89 – 7.85 (m, 2H), 7.54 – 7.34 (m, 1H), 2.63 (s, 3H). ^{13}C NMR (75 MHz, $CDCl_3$) δ (ppm) 192.2, 143.9, 142.6, 139.1, 135.4, 129.7, 125.9, 125.0, 122.9, 26.7.

General procedure for Friedel-Crafts reaction (using acid chloride).

A 8 mL pressure tube was charged with aromatic compound (1.2 mmol, 1.2 equiv.) and propylene carbonate (1 mL) was added, anhydrous Iron(III) Chloride (5 mol %) and the acid chloride (1 mmol, 1 equiv.) were then added in one portion. The reaction mixture was stirred at 80℃ for specified time (3-10h). After completion of the reaction

(monitored by TLC), the mixture was washed with saturated solution of Na_2CO_3 (5 mL). The organic layer was separated from the mixture by centrifugation, washed twice with 2 mL of water (to remove traces of PC and the catalyst), and purified by recrystallization (EtOH / H_2O).

Compound (5a)[19]:

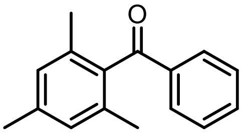

1,3,5-trimethylbenzene (168 µL, 1.2 mmol); benzoyl chloride (117 mg, 1 mmol), Iron(III) Chloride (8 mg, 0.05 mmol, 5 mol %);

Purification (White solid, 173 mg, 0.77 mmol, 77 %). Mp: 35-36 °C. ^{1}H NMR (300 MHz, $CDCl_3$) δ (ppm) 7.82 – 7.79 (m, 2H), 7.60 – 7.55 (m, 1H), 7.47 – 7.41 (m, 2H) 6.90 (s, 2H), 2.33 (s, 3H), 2.08 (s, 6H).^{13}C NMR (75 MHz, $CDCl_3$) δ (ppm) 201.0, 138.6, 137.5, 137.0, 134.3, 133.7, 129.6, 128.9, 128.5, 21.3, 19.5.

Compound (5b)[20]:

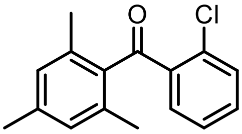

1,3,5-trimethylbenzene (168 µL, 1.2 mmol); 2-chlorobenzoyl chloride (127 µL, 1 mmol), Iron(III) Chloride (8 mg, 0.05 mmol, 5 mol %);

Purification (White solid, 196 mg, 0.76 mmol, 76 %). Mp: 100-102 °C. ^{1}H NMR (300 MHz, $CDCl_3$) δ (ppm) 7.49 – 7.41 (m, 3H), 7.29 – 7.24 (m, 1H), 6.90 (s, 2H), 2.31 (s, 3H), 2.12 (s, 6H).^{13}C NMR (75 MHz, $CDCl_3$) δ (ppm) 199.15, 139.5, 137.8, 137.5, 135.2, 133.4, 132.9, 131.9, 131.6, 128.9, 127.0, 21.3, 19.9.

Compound (5c)[21]:

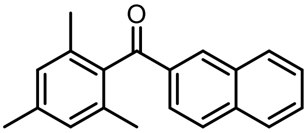

1,3,5-trimethylbenzene (168 µL, 1.2 mmol); 2-naphtoylchloride (191 mg, 1 mmol), Iron(III) Chloride (8 mg, 0.05 mmol, 5 mol %);

Purification (White solid, 227 mg, 0.83 mmol, 83 %). Mp: 81-83 °C. ^{1}H NMR (300 MHz, $CDCl_3$) δ (ppm) 8.17 (s, 1H), 8.03 (dd, J = 8.6, 1.7 Hz, 1H), 7.94 – 7.84 (m, 3H), 7.56 (dddd, J = 25.6, 8.2, 6.9, 1.1 Hz, 2H), 6.94 (s, 2H), 2.37 (s, 3H), 2.12 (s, 6H).^{13}C NMR

(75 MHz, $CDCl_3$) δ (ppm) 200.9, 138.7, 137.2, 136.1, 134.9, 134.5, 132.9, 132.2, 129.9, 128.9, 128.9, 128.5, 128.0, 126.9, 124.4, 21.4, 19.6.

Compound (5d)[22]:

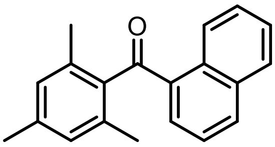
1,3,5-trimethylbenzene (168 µL, 1.2 mmol); 1-naphtoylchloride (191 mg, 1 mmol), Iron(III) Chloride (8 mg, 0.05 mmol, 5 mol %);

Purification (White solid, 218 mg, 0.80 mmol, 80 %). Mp: 98-100 °C. ^{1}H NMR (300 MHz, $CDCl_3$) δ (ppm) 9.20 (d, J = 8.4 Hz, 1H), 8.13 – 7.87 (m, 2H), 7.77 – 7.29 (m, 4H), 6.91 (s, 2H), 2.35 (s, 3H), 2.13 (s, 6H).^{13}C NMR (75 MHz, $CDCl_3$) δ (ppm) 202.9, 138.8, 134.9, 134.5, 134.3, 134.3, 132.5, 130.9, 128.8, 128.7, 128.7, 126.8, 126.3, 124.7, 21.3, 19.6.

Compound (5e)[22]:

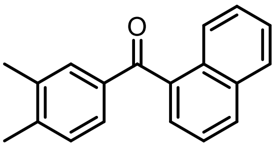
1,2-dimethylbenzene (145 µL, 1.2 mmol); 1-naphtoylchloride (191 mg, 1 mmol), Iron(III) Chloride (8 mg, 0.05 mmol, 5 mol %);

Purification (White solid, 203 mg, 0.78 mmol, 78 %). Mp: 92-94 °C. ^{1}H NMR (300 MHz, $CDCl_3$) δ (ppm) 8.08 – 7.96 (m, 2H), 7.94 – 7.87 (m, 1H), 7.71 – 7.68 (m, 1H), 7.59 – 7.45 (m, 5H), 7.20 (d, J = 7.8 Hz, 1H), 2.34 (s, 3H), 2.30 (s, 3H). ^{13}C NMR (75 MHz, $CDCl_3$) δ (ppm) 198.2, 143.1, 137.1, 137.0, 136.2, 133.8, 131.4, 131.1, 131.0, 129.8, 128.6, 128.5, 127.4, 127.2, 126.5, 125.9, 124.5, 20.3, 19.9.

Compound (5f)[2]:

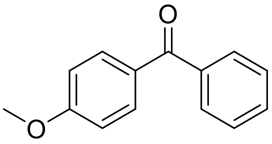
Anisole (132 µL, 1.2 mmol); benzoyl chloride (117 mg, 1 mmol), Iron(III) Chloride (8 mg, 0.05 mmol, 5 mol %);

Purification (White solid, 182 mg, 0.86 mmol, 86 %). Mp: 60-62 °C. ^{1}H NMR (300 MHz, $CDCl_3$) δ (ppm) 7.83 (d, J = 8.9 Hz, 1H), 7.77 – 7.74 (m, 2H), 7.60 – 7.54 (m, 2H) 7.50

– 7.45 (m, 2H), 6.97 (d, *J* = 8.9 Hz, 1H), 3.89 (s, 3H). ^{13}C NMR (75 MHz, $CDCl_3$) δ (ppm) 196.7, 163.4, 138.5, 132.7, 132.0, 130.3, 129.9, 128.3, 113.7, 55.6.

Compound (5g)[23]:

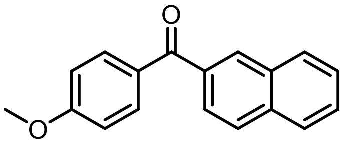
Anisole (132 µL, 1.2 mmol); 2-naphtoylchloride (191 mg, 1 mmol), Iron(III) Chloride (8 mg, 0.05 mmol, 5 mol %);

Purification (White solid, 223 mg, 0.85 mmol, 85 %). Mp: 90-92 °C. ^{1}H NMR (300 MHz, $CDCl_3$) δ (ppm) 8.23 (s, 1H), 7.90 (dd, *J* = 9.0, 7.3 Hz, 6H), 7.65 – 7.49 (m, 2H), 7.00 (d, *J* = 8.8 Hz, 2H), 3.91 (s, 3H). ^{13}C NMR (75 MHz, $CDCl_3$) δ (ppm) 196.7, 163.4, 135.7, 135.2, 132.8, 132.4, 131.3, 130.6, 129.4, 128.3, 128.2, 128.0, 126.9, 126.0, 113.8, 55.7.

Compound (5h)[22]:

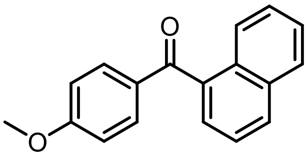
Anisole (132 µL, 1.2 mmol); 1-naphtoylchloride (191 mg, 1 mmol), Iron(III) Chloride (8 mg, 0.05 mmol, 5 mol %);

Purification (White solid, 220 mg, 0.83 mmol, 83 %). Mp: 99-102 °C. ^{1}H NMR (300 MHz, $CDCl_3$) δ (ppm) 7.99 (ddd, *J* = 7.5, 6.0, 1.8 Hz, 2H), 7.92 (dd, *J* = 7.6, 1.9 Hz, 1H), 7.86 (d, *J* = 8.9 Hz, 3H), 7.59 – 7.43 (m, 4H), 6.93 (d, *J* = 8.9 Hz, 2H), 3.87 (s, 3H). ^{13}C NMR (75 MHz, $CDCl_3$) δ (ppm) 196.9, 163.9, 137.2, 133.8, 132.9, 131.2, 131.0, 130.8, 128.5, 127.1, 126.9, 126.5, 125.9, 124.6, 113.8, 55.7.

Compound (5i)[23]:

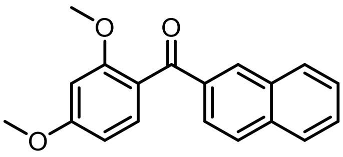
1,3-dimethoxybenzene (157 µL, 1.2 mmol); 2-naphtoylchloride (191 mg, 1 mmol), Iron(III) Chloride (8 mg, 0.05 mmol, 5 mol %);

Purification (White solid, 267 mg, 0.92 mmol, 92 %). Mp: 114-116 °C. ^{1}H NMR (300 MHz, $CDCl_3$) δ (ppm) 8.30 – 8.14 (m, 1H), 7.90 (qd, *J* = 8.5, 2.6 Hz, 4H), 7.62 – 7.48 (m, 2H), 7.47 (d, *J* = 8.3 Hz, 1H), 6.65 – 6.49 (m, 2H), 3.90 (s, 3H), 3.70 (s, 3H). ^{13}C NMR (75 MHz, $CDCl_3$) δ (ppm) 195.7, 163.5, 159.8, 136.3, 135.5, 132.6, 132.4, 131.7, 129.7, 128.2, 127.9, 127.9, 126.6, 125.6, 121.9, 104.7, 99.1, 55.8, 55.7.

Compound (5j)[24]:

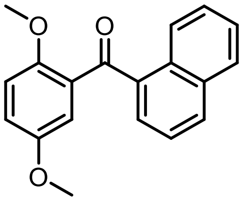

1,4-dimethoxybenzene (190 µL, 1.2 mmol); 1-naphtoylchloride (191 mg, 1 mmol), Iron(III) Chloride (8 mg, 0.05 mmol, 5 mol %);

Purification (White solid, 263 mg, 0.9 mmol, 90 %). Mp: 96-98 °C. ^{1}H NMR (300 MHz, $CDCl_3$) δ (ppm) 8.61 (d, *J* = 7.3 Hz, 1H), 7.98 (d, *J* = 8.2 Hz, 1H), 7.94 – 7.88 (m, 1H), 7.67 – 7.50 (m, 3H), 7.43 (dd, *J* = 8.2, 7.2 Hz, 1H), 7.12 (d, *J* = 3.2 Hz, 1H), 7.05 (dd, *J* = 9.0, 3.2 Hz, 1H), 6.90 (d, *J* = 8.9 Hz, 1H), 3.80 (s, 3H), 3.50 (s, 4H). ^{13}C NMR (75 MHz, $CDCl_3$) δ (ppm) 197.8, 153.6, 152.8, 136.9, 133.9, 132.5, 130.9, 130.8, 129.9, 128.5, 126.4, 126.0, 124.5, 118.9, 115.2, 113.9, 56.6, 56.0.

4- Computational details

All calculations were performed using Gaussian 09 Rev D.01.[25] Following the methodology proposed in *JACS* **2023**, *145,* 13069 by Zimmerman and Devery,[26] geometry optimization and frequency calculations were performed at the DFT level of theory using uB97-D/6-31+G* method (spin multiplicity=6). Liquid phase values of entropy, enthalpy and free energy for each reaction were calculated as follow:

- Entropy in the liquid phase is half of the entropy in the gas phase,
- Entropy in the gas phase is calculated from H , G and T values in the gas phase according to the equation S=[G-H]/T
- Enthalpy in the liquid phase is calculated as the sum of the CPCM energy (solvent=benzene) from a wB97X-D3/def2-TZVP simple point calculation, and the correction to enthalpy provided after the frequency calculation.
- Free energy in the liquid phase is then calculated as G=H-TS with the two previously calculated H and S values in liquid phase.

No transition state could be located on any dissociative scan using the opt=TS option of Gaussian starting from any point of higher energy in the scan. Geometries were plotted using Chemcraft visualization software.[27] Optimized geometries and their coordinates are listed in the table below.

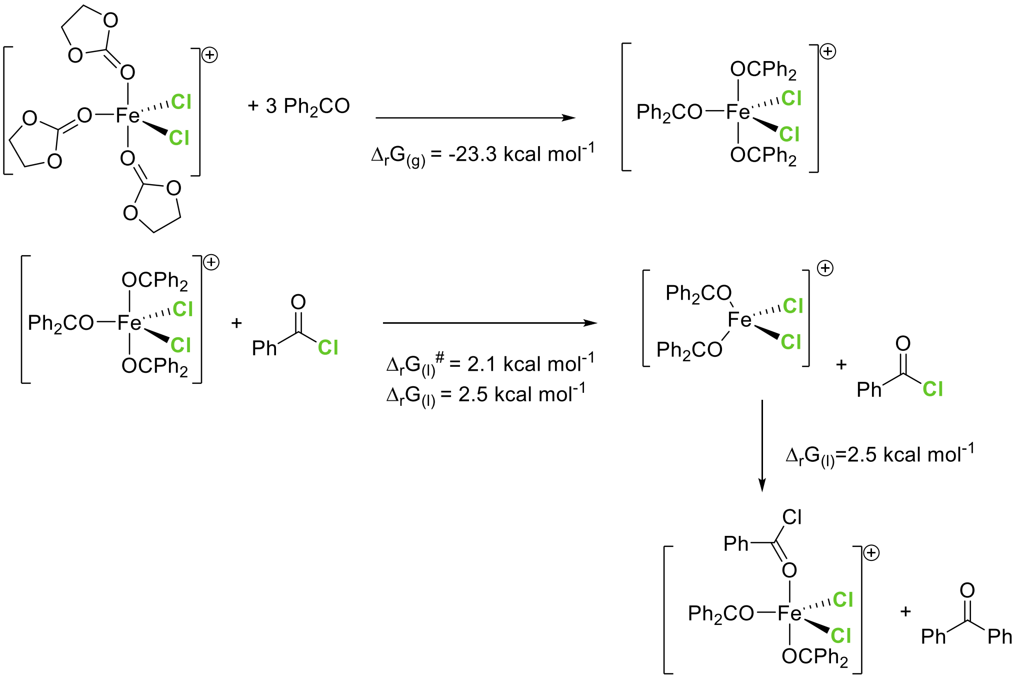

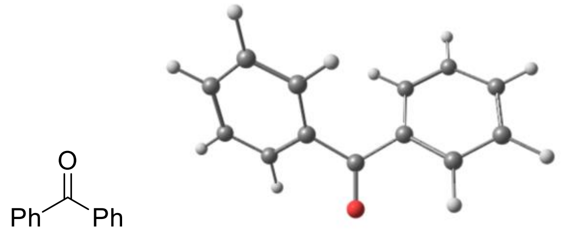				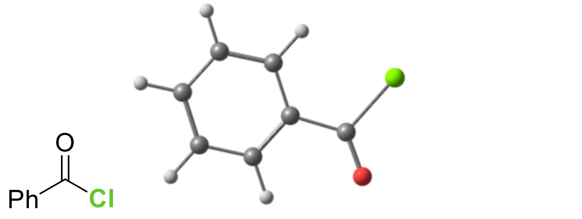			
8	3.083026000	10.188559000	2.949023000	8	2.770372000	10.342574000	3.317901000
6	2.143387000	10.740061000	2.368366000	6	2.151167000	10.766414000	2.380544000
6	0.806974000	10.053877000	2.270609000	6	2.333286000	12.089291000	1.726345000
6	0.775598000	8.643367000	2.329547000	6	3.339994000	12.920715000	2.275261000
1	1.721971000	8.106885000	2.406083000	6	1.579223000	12.543145000	0.622924000
6	-0.443808000	7.959316000	2.291465000	6	3.584329000	14.182522000	1.726066000
1	-0.457727000	6.867906000	2.322063000	6	1.830843000	13.808894000	0.079439000
6	-1.651609000	8.676855000	2.222533000	6	2.830447000	14.629376000	0.627073000
1	-2.603908000	8.142713000	2.200628000	1	3.915760000	12.560439000	3.127304000
6	-1.632876000	10.080135000	2.190158000	1	0.803912000	11.910281000	0.195101000
1	-2.569518000	10.640209000	2.154997000	1	4.361591000	14.818099000	2.153646000
6	-0.410038000	10.766459000	2.206967000	1	1.244884000	14.154642000	-0.773614000
1	-0.395063000	11.856678000	2.194506000	1	3.022032000	15.615178000	0.198816000
6	2.328177000	12.103103000	1.756688000	17	0.822863000	9.688046000	1.646324000
6	3.311166000	12.954093000	2.307530000				
6	1.616632000	12.525310000	0.612881000				
6	3.552614000	14.213380000	1.748502000				

6	1.874707000	13.779296000	0.040531000
6	2.834624000	14.628856000	0.612640000
1	3.872501000	12.604122000	3.174551000
1	0.880493000	11.860367000	0.160376000
1	4.303492000	14.871178000	2.190963000
1	1.329087000	14.091290000	-0.852323000
1	3.028361000	15.609150000	0.172067000

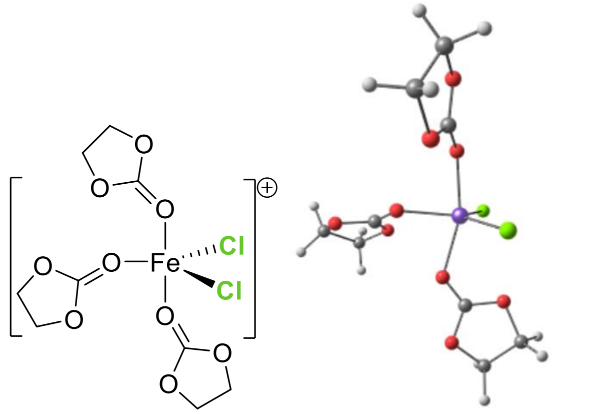

26	5.952954000	10.020603000	2.953393000
17	6.446786000	11.146643000	4.766957000
17	6.269819000	11.014786000	1.046993000
8	5.263243000	8.057313000	2.967896000
8	7.814055000	8.956940000	2.944160000
6	5.512497000	7.144509000	2.172286000
6	8.947658000	9.450035000	2.964483000
8	10.044006000	8.697912000	3.090302000
8	9.197376000	10.754094000	2.862022000
6	10.622322000	10.977535000	3.101371000
8	5.215557000	5.873264000	2.434676000
8	6.080294000	7.334732000	0.980489000
6	5.479773000	5.079801000	1.232244000
1	5.986734000	4.160529000	1.544525000
1	4.511289000	4.851044000	0.767920000
6	6.356980000	6.026767000	0.385256000
1	7.433132000	5.837103000	0.493793000
1	6.074461000	6.073659000	-0.671786000
6	3.119410000	10.281379000	4.009822000
8	3.888820000	10.425644000	3.054580000
8	1.870210000	10.744319000	3.992701000
8	3.448965000	9.654321000	5.141999000
6	1.290743000	10.567573000	5.323988000
6	2.265326000	9.574459000	5.996447000
1	1.271556000	11.552869000	5.808065000
1	0.274279000	10.179157000	5.197674000
1	2.564848000	9.862360000	7.010014000
1	1.912791000	8.534621000	5.976539000
6	11.216396000	9.562140000	2.936004000
1	11.938313000	9.287365000	3.712639000
1	11.632903000	9.379684000	1.936302000
1	10.975930000	11.701088000	2.359235000
1	10.725332000	11.379551000	4.117872000

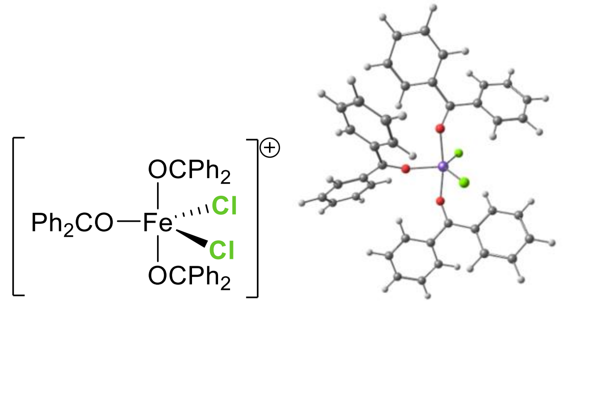

26	5.102044000	9.767410000	2.686956000
17	5.485303000	10.861197000	4.590649000
17	5.170185000	10.491966000	0.571725000
8	3.088134000	10.120742000	2.834845000
8	4.546292000	7.874236000	2.824852000
8	7.052927000	9.114273000	2.627692000
6	2.128895000	10.737231000	2.285391000
6	0.817832000	10.057237000	2.246287000
6	0.783659000	8.643179000	2.336325000
1	1.722865000	8.095894000	2.400139000
6	-0.438007000	7.968232000	2.333463000
1	-0.455704000	6.878808000	2.392263000
6	-1.641950000	8.694289000	2.260539000
1	-2.597236000	8.166392000	2.264068000
6	-1.619921000	10.097666000	2.191492000
1	-2.554608000	10.658691000	2.154595000
6	-0.397819000	10.780002000	2.181448000
1	-0.377624000	11.869047000	2.156342000
6	4.601339000	6.628021000	2.601546000
6	5.714158000	6.050483000	1.829019000
6	6.345123000	6.823214000	0.824416000
1	5.963007000	7.814190000	0.589138000
6	7.436040000	6.302625000	0.125261000
1	7.907271000	6.895459000	-0.659495000
6	7.926807000	5.021939000	0.435431000
1	8.787714000	4.624350000	-0.104417000
6	7.315843000	4.252299000	1.440993000
1	7.705174000	3.263595000	1.687491000
6	6.207929000	4.755379000	2.129682000
1	5.745580000	4.172687000	2.926220000
6	8.225404000	9.401526000	3.011072000
6	9.166634000	8.285460000	3.238588000
6	8.656333000	7.005597000	3.562742000
1	7.581100000	6.878041000	3.670139000
6	9.525260000	5.929845000	3.751761000
1	9.126019000	4.950387000	4.017544000
6	10.912341000	6.109433000	3.596681000

1	11.590047000	5.266011000	3.740164000
6	11.428346000	7.370779000	3.254488000
1	12.502323000	7.505233000	3.119237000
6	10.563687000	8.457462000	3.081478000
1	10.958728000	9.431692000	2.795346000
6	3.503905000	5.800800000	3.140700000
6	3.134663000	4.576549000	2.529189000
6	2.743955000	6.291836000	4.233359000
6	2.037316000	3.855024000	3.011821000
6	1.659553000	5.558227000	4.718533000
6	1.304798000	4.339178000	4.109411000
1	3.683196000	4.219447000	1.658393000
1	3.022086000	7.242177000	4.688155000
1	1.747921000	2.920944000	2.528747000
1	1.088081000	5.932377000	5.568825000
1	0.454106000	3.769956000	4.488067000
6	8.638734000	10.801643000	3.212737000
6	9.534698000	11.144925000	4.254755000
6	8.093873000	11.820013000	2.395191000
6	9.865924000	12.485059000	4.479329000
6	8.454532000	13.152509000	2.605767000
6	9.332772000	13.488371000	3.652228000
1	9.928149000	10.364618000	4.905697000
1	7.419740000	11.552410000	1.583681000
1	10.535119000	12.748555000	5.299351000
1	8.050490000	13.931437000	1.957499000
1	9.600779000	14.532282000	3.823883000
6	2.332288000	12.084891000	1.725810000
6	3.291756000	12.939700000	2.320725000
6	1.628497000	12.515341000	0.575198000
6	3.520207000	14.209910000	1.787647000
6	1.885340000	13.777562000	0.029879000
6	2.823529000	14.628258000	0.639036000
1	3.824426000	12.608085000	3.211275000
1	0.919304000	11.842526000	0.093872000
1	4.241661000	14.875543000	2.263844000
1	1.359048000	14.097214000	-0.870449000
1	3.013690000	15.616724000	0.217315000

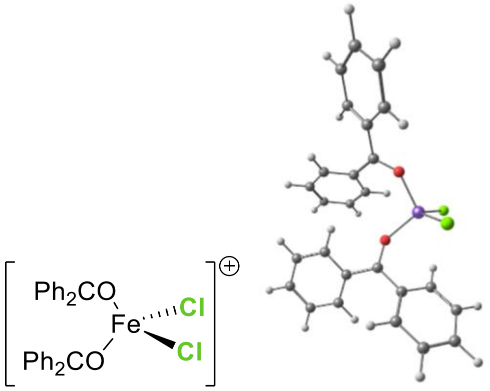

26	5.821275000	10.039119000	2.746971000
17	5.768236000	11.100704000	4.636115000
17	5.285028000	11.137714000	0.947121000
8	4.577819000	8.575784000	2.951966000
8	7.552319000	9.220127000	2.536275000
6	4.457169000	7.312124000	2.790362000
6	5.409231000	6.598935000	1.920769000

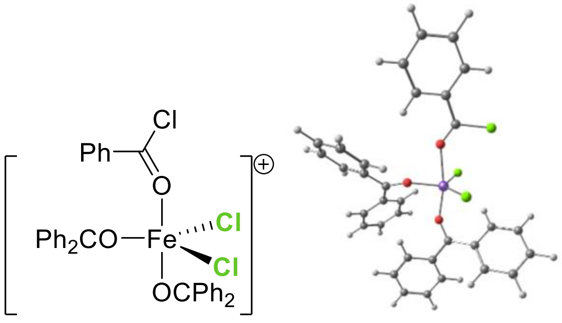

26	5.134132000	9.481793000	2.245790000
17	5.079252000	10.756129000	4.037084000
17	5.219814000	10.102219000	0.124844000
8	2.875612000	9.623089000	2.149533000
8	4.606657000	7.633937000	2.581487000
8	7.073984000	8.969169000	2.402569000
6	2.012914000	10.485406000	2.015458000

6	5.938934000	7.254316000	0.782711000	6	0.570681000	10.207071000	2.125251000
1	5.588349000	8.253350000	0.521938000	6	0.196072000	8.874907000	2.443421000
6	6.866584000	6.601728000	-0.032427000	1	0.970338000	8.122880000	2.590711000
1	7.252962000	7.102068000	-0.920918000	6	-1.153090000	8.540374000	2.567550000
6	7.294777000	5.301336000	0.290014000	1	-1.436657000	7.515871000	2.812769000
1	8.024613000	4.797242000	-0.345255000	6	-2.143028000	9.521436000	2.375091000
6	6.784072000	4.646649000	1.424692000	1	-3.197411000	9.257200000	2.471412000
1	7.120739000	3.639721000	1.675172000	6	-1.778832000	10.841177000	2.058622000
6	5.839035000	5.285079000	2.235138000	1	-2.546932000	11.600460000	1.908770000
1	5.453193000	4.791282000	3.126773000	6	-0.430126000	11.188815000	1.933974000
6	8.765978000	9.424426000	2.907725000	1	-0.155188000	12.213124000	1.688906000
6	9.669193000	8.272822000	2.920556000	6	4.708900000	6.372231000	2.493747000
6	9.126018000	6.961679000	2.966285000	6	5.856043000	5.770623000	1.794270000
1	8.046417000	6.831878000	3.018637000	6	6.468091000	6.469893000	0.725468000
6	9.973396000	5.854429000	2.959716000	1	6.046202000	7.414574000	0.387405000
1	9.552295000	4.849906000	3.011634000	6	7.591740000	5.934371000	0.093089000
6	11.368298000	6.033384000	2.884191000	1	8.047726000	6.467804000	-0.741553000
1	12.027836000	5.164101000	2.875189000	6	8.134676000	4.714435000	0.534674000
6	11.916044000	7.326185000	2.809258000	1	9.020058000	4.305504000	0.045326000
1	12.994992000	7.460194000	2.726327000	6	7.544490000	4.020574000	1.605777000
6	11.075964000	8.442885000	2.828214000	1	7.973670000	3.079700000	1.952823000
1	11.494386000	9.443962000	2.735203000	6	6.404595000	4.537309000	2.229625000
6	3.362948000	6.637538000	3.486246000	1	5.958106000	4.016496000	3.076302000
6	2.836618000	5.406561000	3.010102000	6	8.184990000	9.386948000	2.861295000
6	2.780430000	7.254528000	4.626720000	6	9.221680000	8.379680000	3.152041000
6	1.763997000	4.803521000	3.671251000	6	8.836199000	7.041391000	3.412092000
6	1.720894000	6.635376000	5.289019000	1	7.778209000	6.787952000	3.424306000
6	1.211320000	5.411155000	4.813083000	6	9.804187000	6.067928000	3.661762000
1	3.242765000	4.957480000	2.104543000	1	9.499128000	5.043154000	3.876318000
1	3.183004000	8.202293000	4.983304000	6	11.169621000	6.408274000	3.633209000
1	1.349439000	3.868452000	3.293449000	1	11.925155000	5.644293000	3.824416000
1	1.285718000	7.101448000	6.173556000	6	11.564714000	7.727406000	3.352872000
1	0.376069000	4.935183000	5.329187000	1	12.623591000	7.985529000	3.312173000
6	9.187974000	10.776270000	3.282473000	6	10.599259000	8.711868000	3.116751000
6	10.115382000	10.982318000	4.336173000	1	10.903056000	9.728989000	2.872473000
6	8.609326000	11.894369000	2.629342000	17	2.517850000	12.163920000	1.679548000
6	10.442861000	12.282517000	4.730784000	6	3.643926000	5.566934000	3.115318000
6	8.966168000	13.188477000	3.010052000	6	3.336359000	4.263919000	2.649119000
6	9.875713000	13.384330000	4.065942000	6	2.855840000	6.144538000	4.144908000
1	10.528068000	10.124951000	4.867111000	6	2.272445000	3.551489000	3.212834000
1	7.922368000	11.741057000	1.796990000	6	1.805726000	5.420504000	4.711996000
1	11.132344000	12.439358000	5.560886000	6	1.512414000	4.123570000	4.247214000
1	8.535839000	14.044209000	2.489271000	1	3.903519000	3.836148000	1.823352000
1	10.140205000	14.397376000	4.372476000	1	3.093040000	7.149063000	4.494646000
				1	2.029506000	2.555620000	2.840523000
				1	1.215737000	5.859507000	5.517693000
				1	0.687726000	3.561863000	4.689067000
				6	8.422072000	10.821889000	3.077149000
				6	9.202970000	11.266826000	4.173506000
				6	7.823783000	11.771545000	2.213785000
				6	9.369503000	12.635831000	4.402644000
				6	8.022811000	13.136166000	2.430612000
				6	8.787206000	13.570519000	3.528725000
				1	9.634117000	10.537758000	4.859071000
				1	7.240428000	11.430882000	1.360159000
				1	9.947978000	12.975482000	5.262574000
				1	7.578907000	13.862758000	1.749209000

	1	8.926949000	14.638293000	3.704885000

5-References

[1] G. Oss, J. Ho, T. V. Nguyen. *Eur. J. Org. Chem.* **2018**, *3974–3981*.
[2] G. Pandey, S. K. Tiwari, B. Singh, K. Vankab, S. Jain. *Chem. Commun.*, **2017**, *53*, 12337-12340.
[3] J. A. Terrett, J. D. Cuthbertson, V. W. Shurtleff, D. W. C. MacMillan. Nature, **2015,** *524*, 330–334.
[4] M. Hosseini, S. H. Sharghi. J. Org. Chem. **2004**, *69*, 6953–6956.
[5] S. P. Chavan, S. Garai, A. K. Dutta, S. Pal. *Eur. J. Org. Chem.* **2012**, 6841-6845.
[6] L. Zhanga, X. Hu. *Chem. Sci.*, **2017**, 8, 7009-7013.
[7] K. Polidano, B. G. Reed-Berendt, A. Basset, A. J. A. Watson, J. M. J. Williams, L. C. Morrill. *Org. Lett.* **2017**, *19*, 6716–6719.
[8] D. Uno, H. Minami, S. Otsuka, K. Nogi, H. Yorimitsu. *Chem. Asian J.* **2018**, *13*, 2397 – 2400.
[9] H. Tokuyama, S. Yokoshima, T. Yamashita, T. Fukuyama. *Tetrahedron Lett.* **1998**, *39,* 3189-3192.
[10] N. Al-Rifai, H. Rücker, Sa. Amslinger. *Chem. Eur. J.* **2013**, *19*, 15384 – 15395.
[11] V. Dauksas, P. Gaidelis, E. Udrenaite, L. Labanauskas, G. Gasperaviciene, L. Gumbaragite, D. Ramanauskas. *Khim.-Farm. Zh.* **1989**, *23*, 1466-1470.
[12] X. Rao, H. Ishitani, W-J Yoo, S. Kobayashi. *Asian J. Org. Chem*. *2019*, 316 – 319.
[13] J. Ruan, O. Saidi, J. A. Iggo, J. Xiao. *J. Am. Chem. Soc*. **2015**, 56, 10510–10511.
[14] P. H. Tran, P. E. Hansen, H. M. Hoang, D-K. N. Chau, T. N. Le. *Tetrahedron Lett,* **1998**, *39,* 2187-2192.
[15] L. Pengfei, Z. Jinbo, L. Jie, Y. Yanming, S. Zhonggang, L. Yumeng, G. Aiying. WO2021082997.
[16] R. A. Swyka, W. G. Shuler, B. J. Spinello, W. Zhang, C. Lan, M. J. Krische. *J. Am. Chem. Soc*. **2019**, 141, 6864–6868.
[17] L-X. Liu, X-Q. Wang, J-M. Yan, Y. Li, C-J. Sun, W. Chen, B. Zhou, H-B. Zhang, X-D. Yang. *Eur. J. Med. Chem.* **2013***,* 66*,* 423-437.
[18] V. Morizur, D. Hector, S. Olivero, J. R. Desmurs, E. Duñach. *Eur. J. Org. Chem.* **2016***,* 3126-3129.
[19] F. Xu, D. Lib, W. Han. *Green Chem.,* **2019**, *21*, 2911-2915.
[20] Y-J. Lin, Y-P. Wu, M. Thul, M-W. Hung, S-H. Chou, W-T. Chen, W. Lin, M. Lin, D. M. Reddy, H-R. Wu, W-Y. Ho, S-Y. Luo. *Molecules* **2020**, 25, 352.
[21] H. Park, S. Lee. *Adv. Synth. Catal*. **2023**, 365, 3167–3171.
[22] M.B. Z. Khalid, G. Pallikonda, R.N. P. Tulichala, M. Chakravarty. *Tetrahedron* **2016**, *72,* 2094-2101.
[23] S. Shi, M. Szostak. *Org. Lett*. **2016**, 18, 22, 5872–5875.
[24] H. Waldmann, R. Postuwka, E. Maly. Chem. Ber. **1950**, *83,* 171-8.
[25] M. J. Frisch, G. W. Trucks, H. B. Schlegel, G. E. Scuseria, M. A. Robb, J. R. Cheeseman, G. Scalmani, V. Barone, B. Mennucci, G. A. Petersson, H. Nakatsuji, M. Caricato, X. Li, H. P. Hratchian, A. F. Izmaylov, J. Bloino, G. Zheng, J. L. Sonnenberg, M. Hada, M. Ehara, K. Toyota, R. Fukuda, J. Hasegawa, M. Ishida, T. Nakajima, Y. Honda, O. Kitao, H. Nakai, T. Vreven, J. A. Montgomery, Jr., J. E. Peralta, F. Ogliaro, M. Bearpark, J. J. Heyd, E. Brothers, K. N. Kudin, V. N. Staroverov, T. Keith, R. Kobayashi, J. Normand, K. Raghavachari, A. Rendell, J. C. Burant, S.

S. Iyengar, J. Tomasi, M. Cossi, N. Rega, J. M. Millam, M. Klene, J. E. Knox, J. B. Cross, V. Bakken, C. Adamo, J. Jaramillo, R. Gomperts, R. E. Stratmann, O. Yazyev, A. J. Austin, R. Cammi, C. Pomelli, J. W. Ochterski, R. L. Martin, K. Morokuma, V. G. Zakrzewski, G. A. Voth, P. Salvador, J. J. Dannenberg, S. Dapprich, A. D. Daniels, O. Farkas, J. B. Foresman, J. V. Ortiz, J. Cioslowski, and D. J. Fox, *Gaussian 09, Revision D.01*, Gaussian, Inc., Wallingford CT, 2013.
[26] S. R.Todtz, C. W. Schneider, T. Malakar, C. Anderson, H. Koska, P. M. Zimmerman, J. J.Devery, III, *J. Am. Chem. Soc.* **2023**, *145*, 13069−13080.
[27] Zhurko G. A. Chemcraft - graphical program for visualization of quantum chemistry computations. Ivanovo, Russia, 2005.

6-Copies of NMR spectra

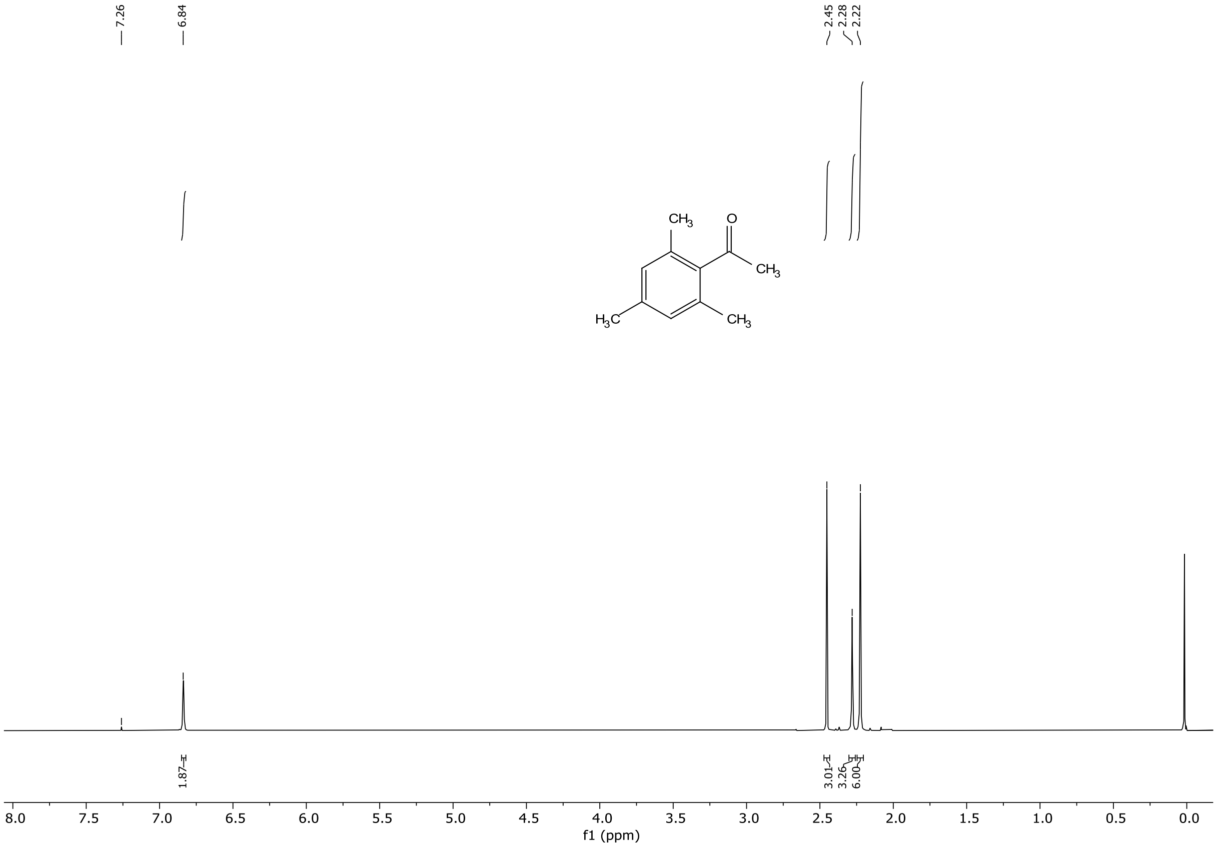

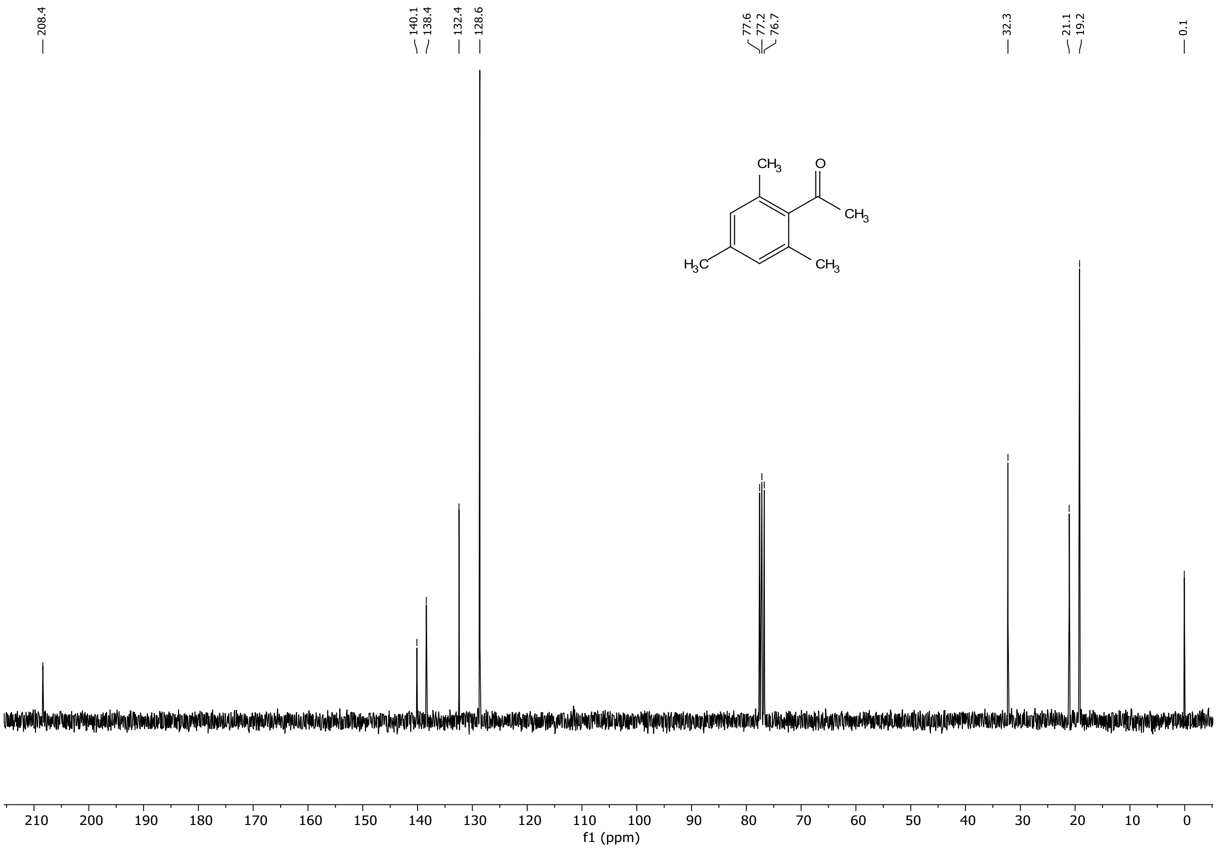

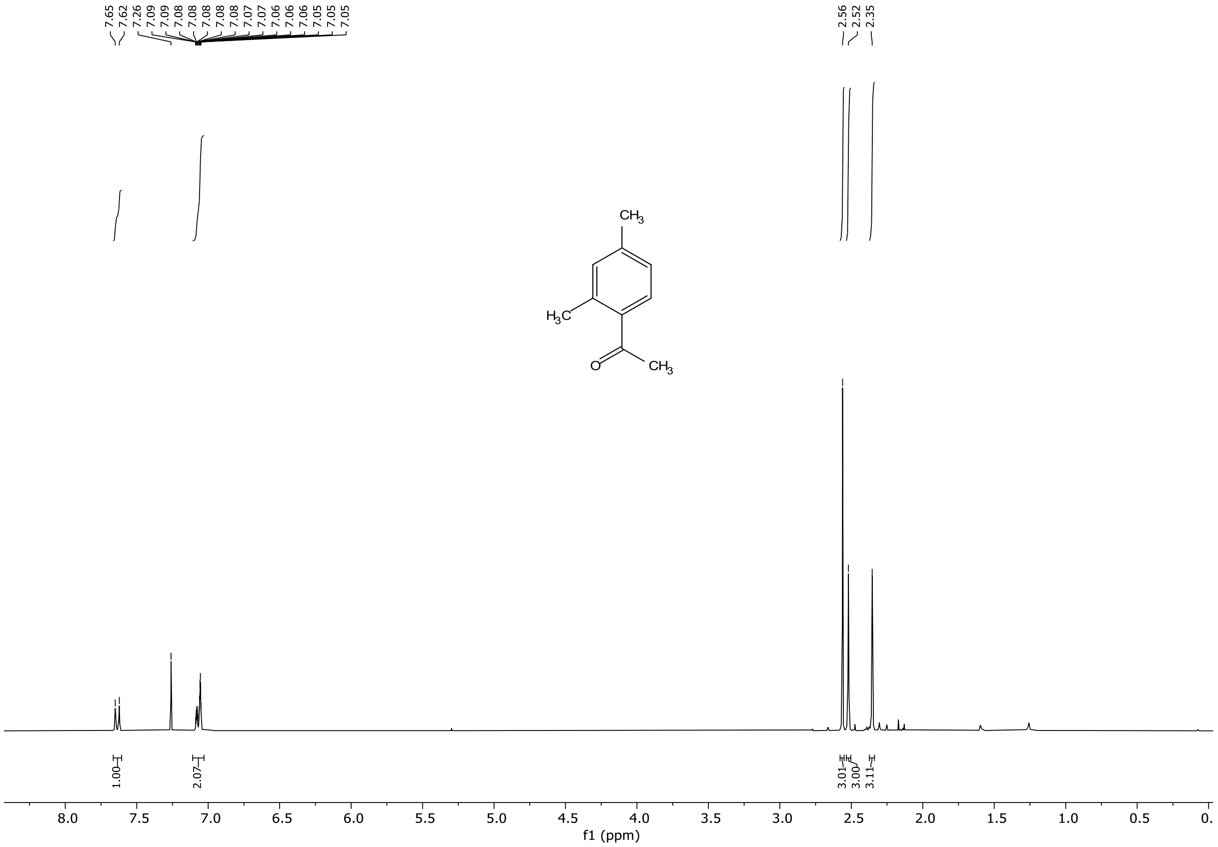
7.65
7.62
7.26
7.09
7.09
7.08
7.08
7.08
7.08
7.07
7.06
7.06
7.06
7.05
7.05
7.05
2.56
2.52
2.35
CH3
H3C
O
CH3
1.00
2.07
3.01
3.00
3.11
8.0 7.5 7.0 6.5 6.0 5.5 5.0 4.5 4.0 3.5 3.0 2.5 2.0 1.5 1.0 0.5 0.
f1 (ppm)

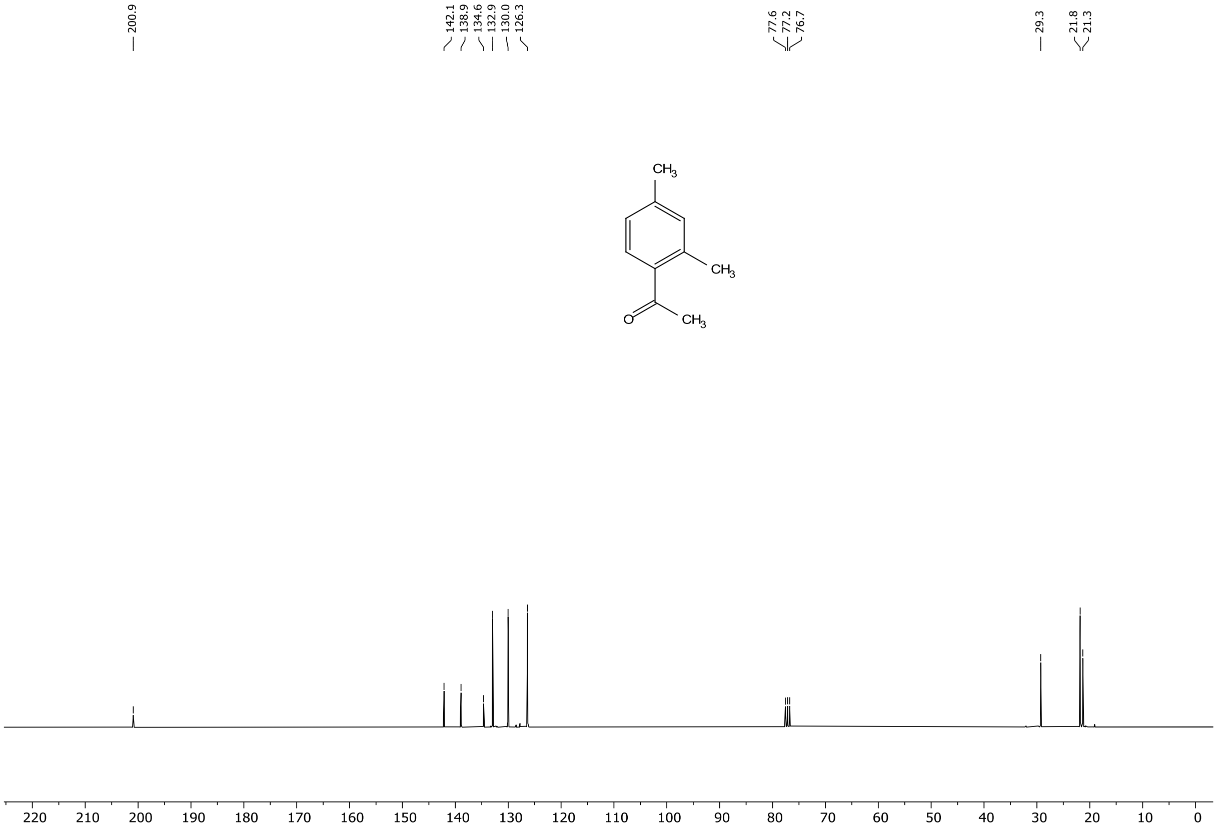
200.9
142.1
138.9
134.6
132.9
130.0
126.3
77.6
77.2
76.7
29.3
21.8
21.3
CH3
CH3
O
CH3
220 210 200 190 180 170 160 150 140 130 120 110 100 90 80 70 60 50 40 30 20 10 0
f1 (ppm)

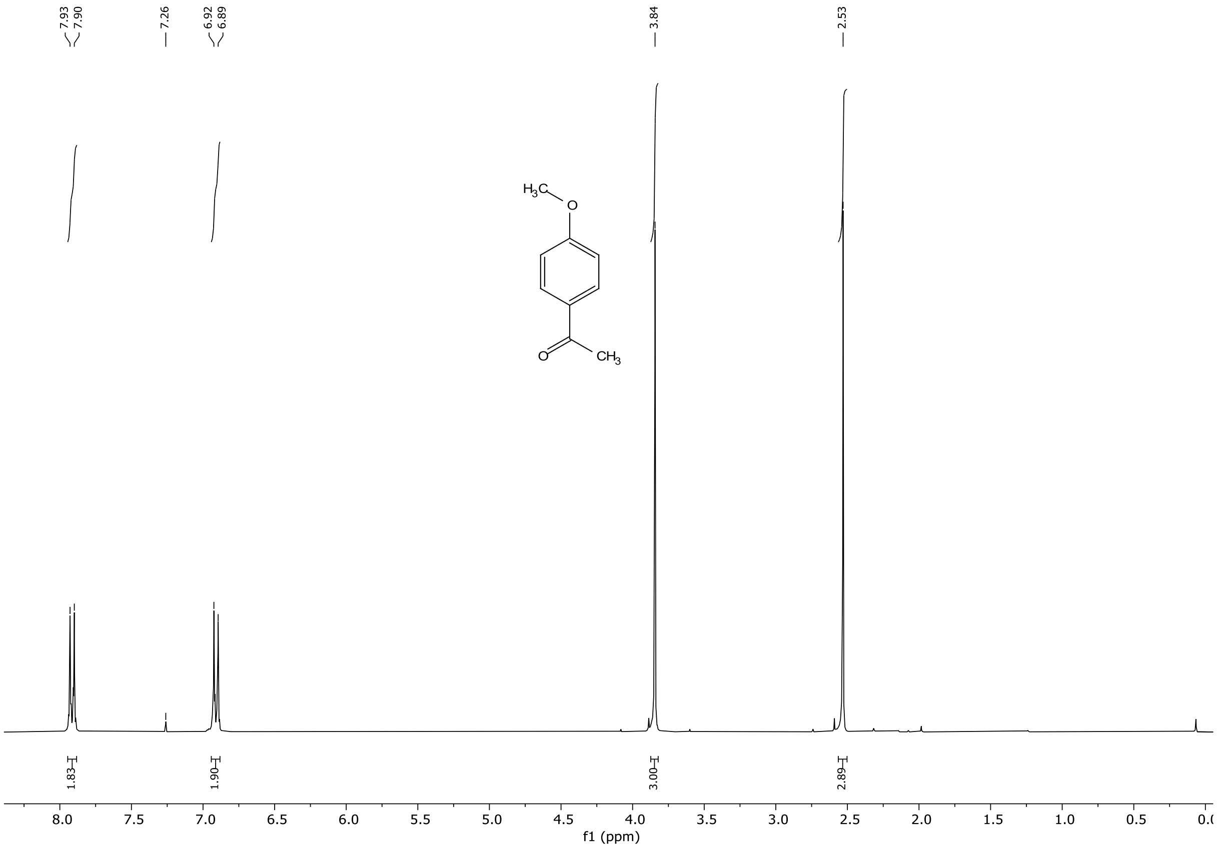
7.93
7.90
7.26
6.92
6.89
3.84
2.53
H_3C
O
O
CH_3
1.83
1.90
3.00
2.89
8.0 7.5 7.0 6.5 6.0 5.5 5.0 4.5 4.0 3.5 3.0 2.5 2.0 1.5 1.0 0.5
f1 (ppm)

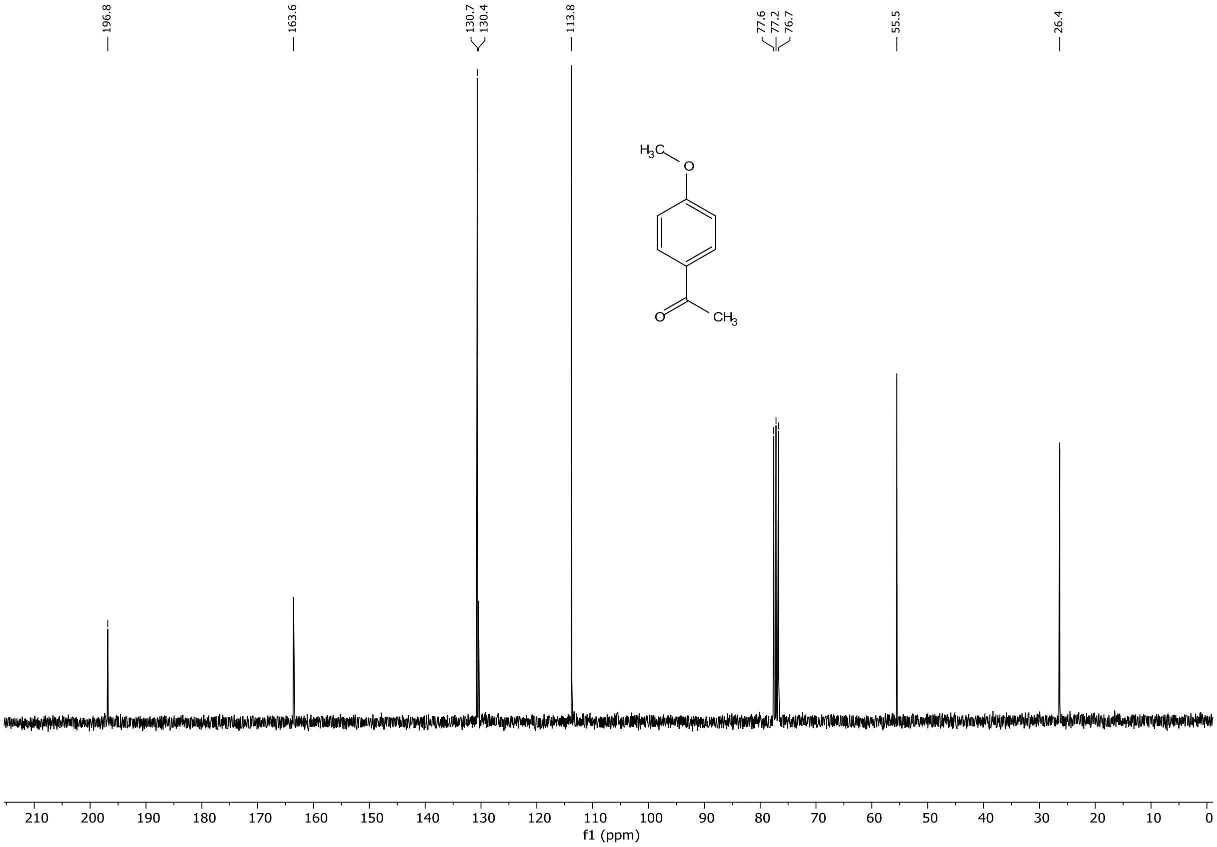
196.8
163.6
130.7
130.4
113.8
77.6
77.2
76.7
55.5
26.4
H_3C
O
O
CH_3
210 200 190 180 170 160 150 140 130 120 110 100 90 80 70 60 50 40 30 20 10 0
f1 (ppm)

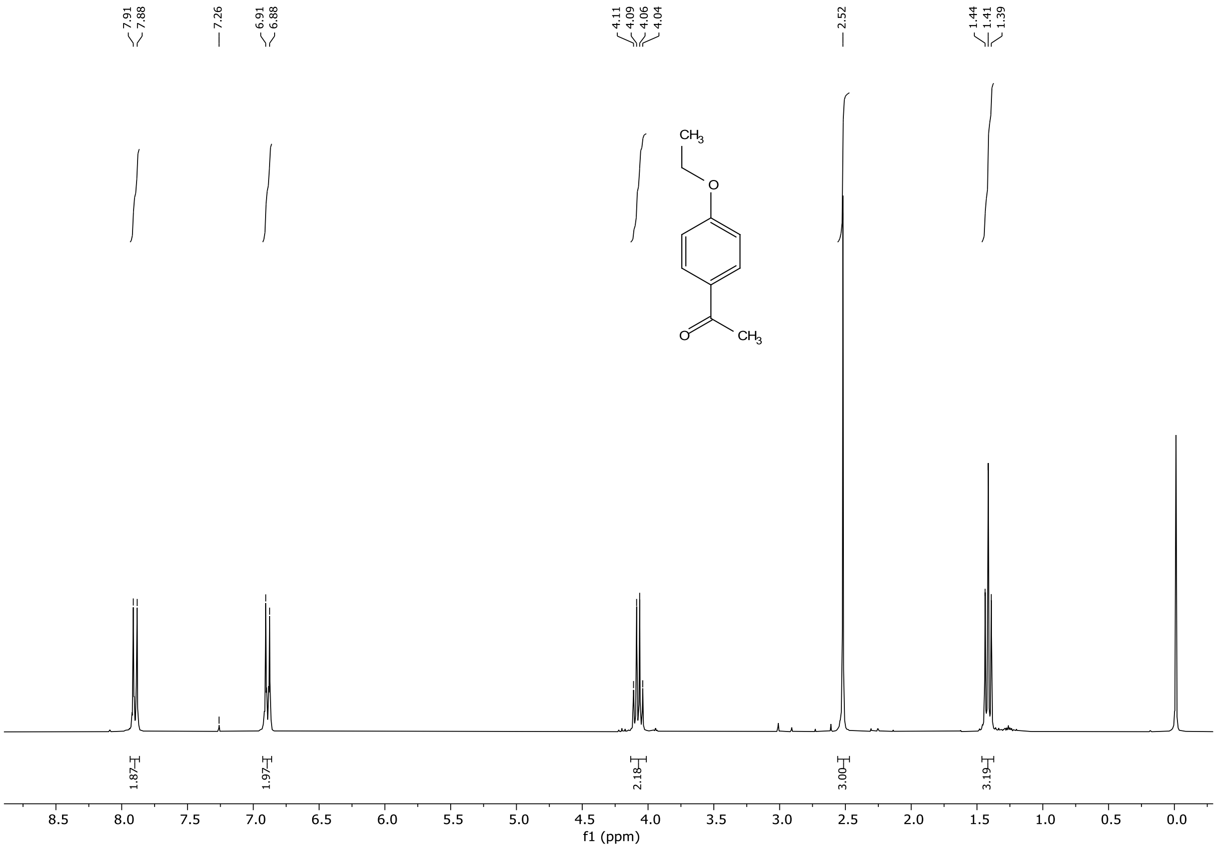
7.91
7.88
7.26
6.91
6.88
4.11
4.09
4.06
4.04
2.52
1.44
1.41
1.39
CH3
O
O
CH3
1.87
1.97
2.18
3.00
3.19
8.5 8.0 7.5 7.0 6.5 6.0 5.5 5.0 4.5 4.0 3.5 3.0 2.5 2.0 1.5 1.0 0.5 0.0
f1 (ppm)

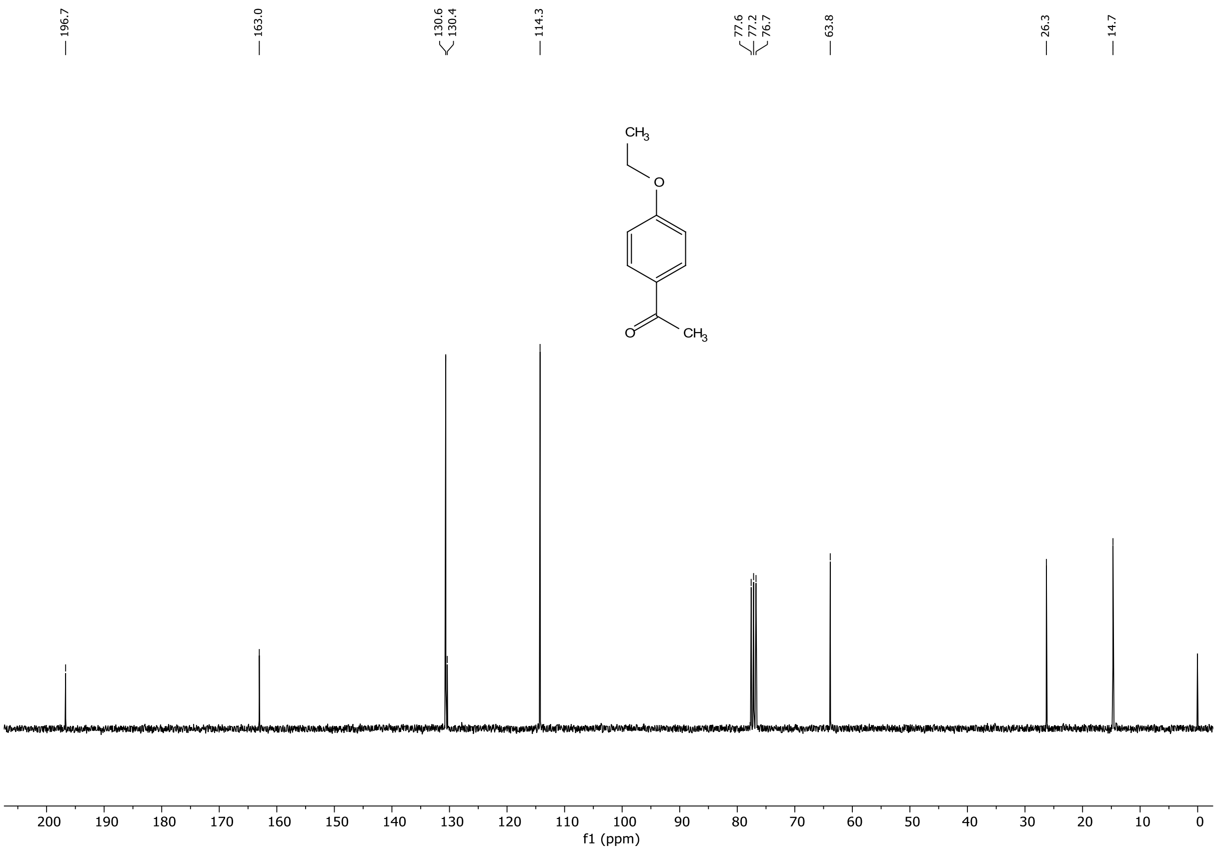
196.7
163.0
130.6
130.4
114.3
77.6
77.2
76.7
63.8
26.3
14.7
CH3
O
O
CH3
200 190 180 170 160 150 140 130 120 110 100 90 80 70 60 50 40 30 20 10 0
f1 (ppm)

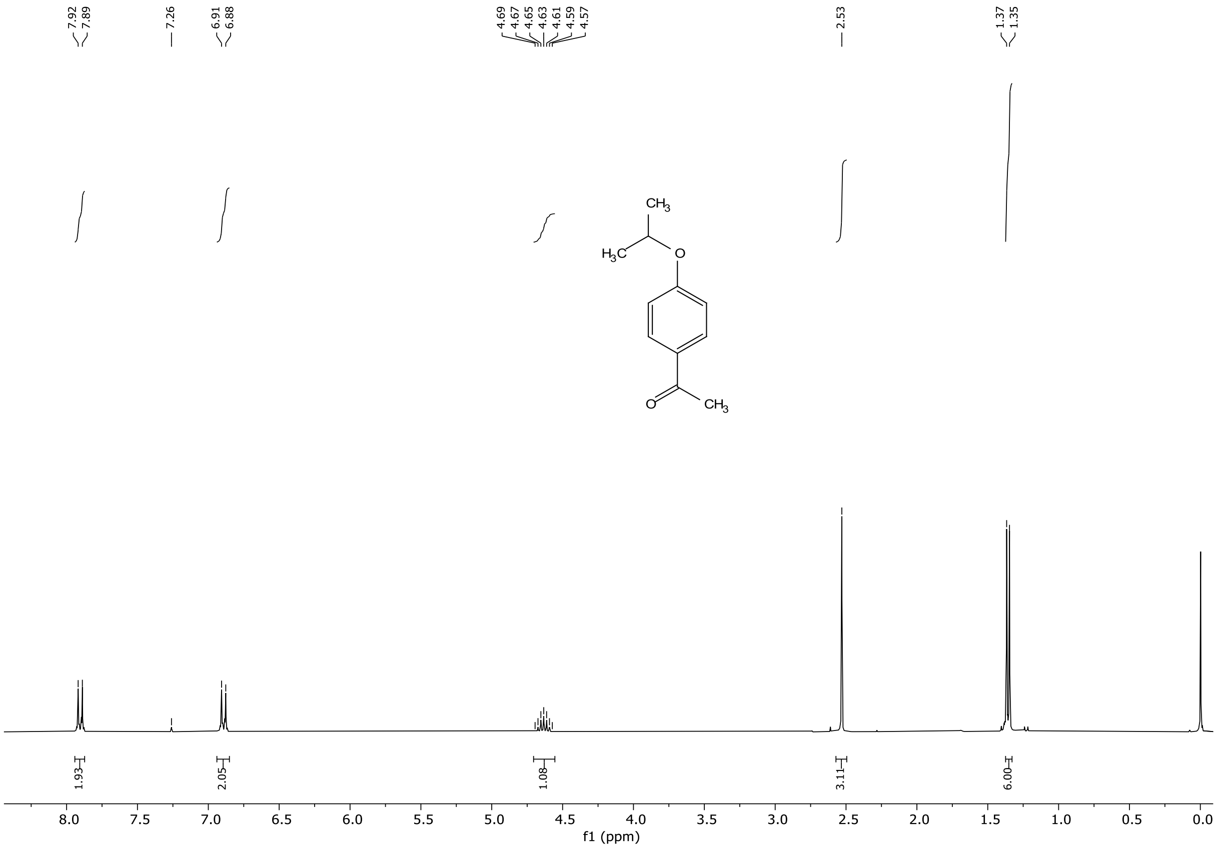
7.92
7.89
7.26
6.91
6.88
4.69
4.67
4.65
4.63
4.61
4.59
4.57
2.53
1.37
1.35
CH3
H3C
O
O
CH3
1.93
2.05
1.08
3.11
6.00
8.0 7.5 7.0 6.5 6.0 5.5 5.0 4.5 4.0 3.5 3.0 2.5 2.0 1.5 1.0 0.5 0.0
f1 (ppm)

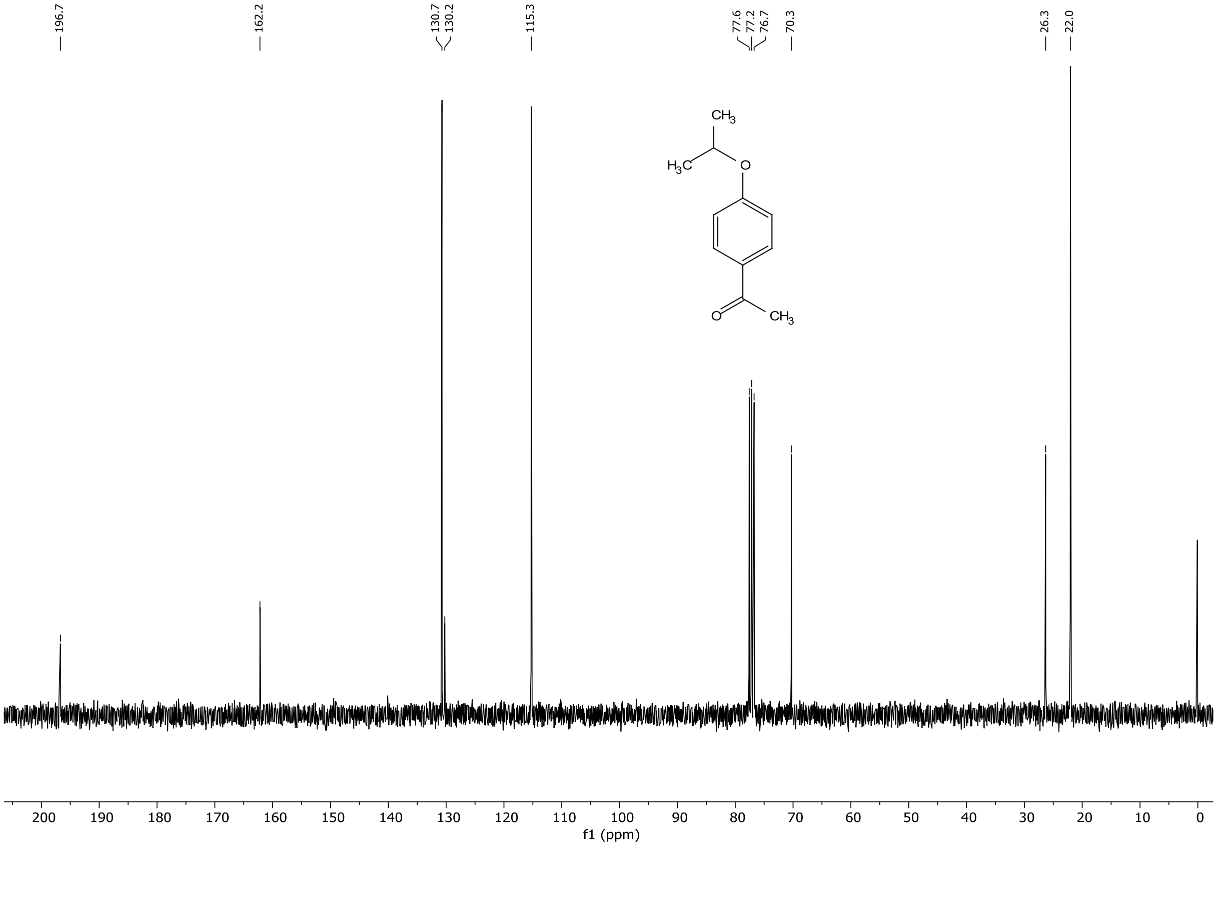
196.7
162.2
130.7
130.2
115.3
77.6
77.2
76.7
70.3
26.3
22.0
CH3
H3C
O
O
CH3
200 190 180 170 160 150 140 130 120 110 100 90 80 70 60 50 40 30 20 10 0
f1 (ppm)

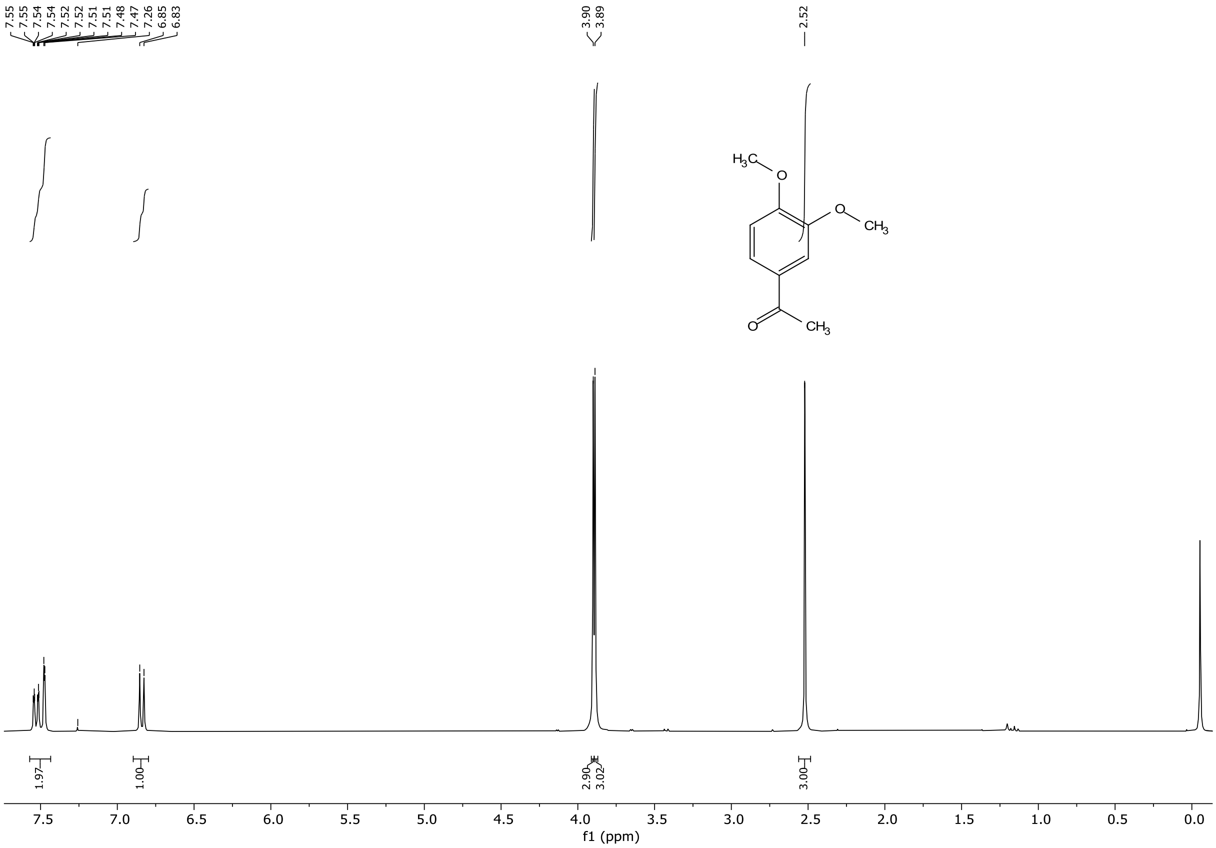
7.55
7.55
7.54
7.54
7.52
7.52
7.51
7.51
7.48
7.47
7.26
6.85
6.83
3.90
3.89
2.52
H3C
O
O
CH3
O
CH3
1.97
1.00
2.90
3.02
3.00
f1 (ppm)

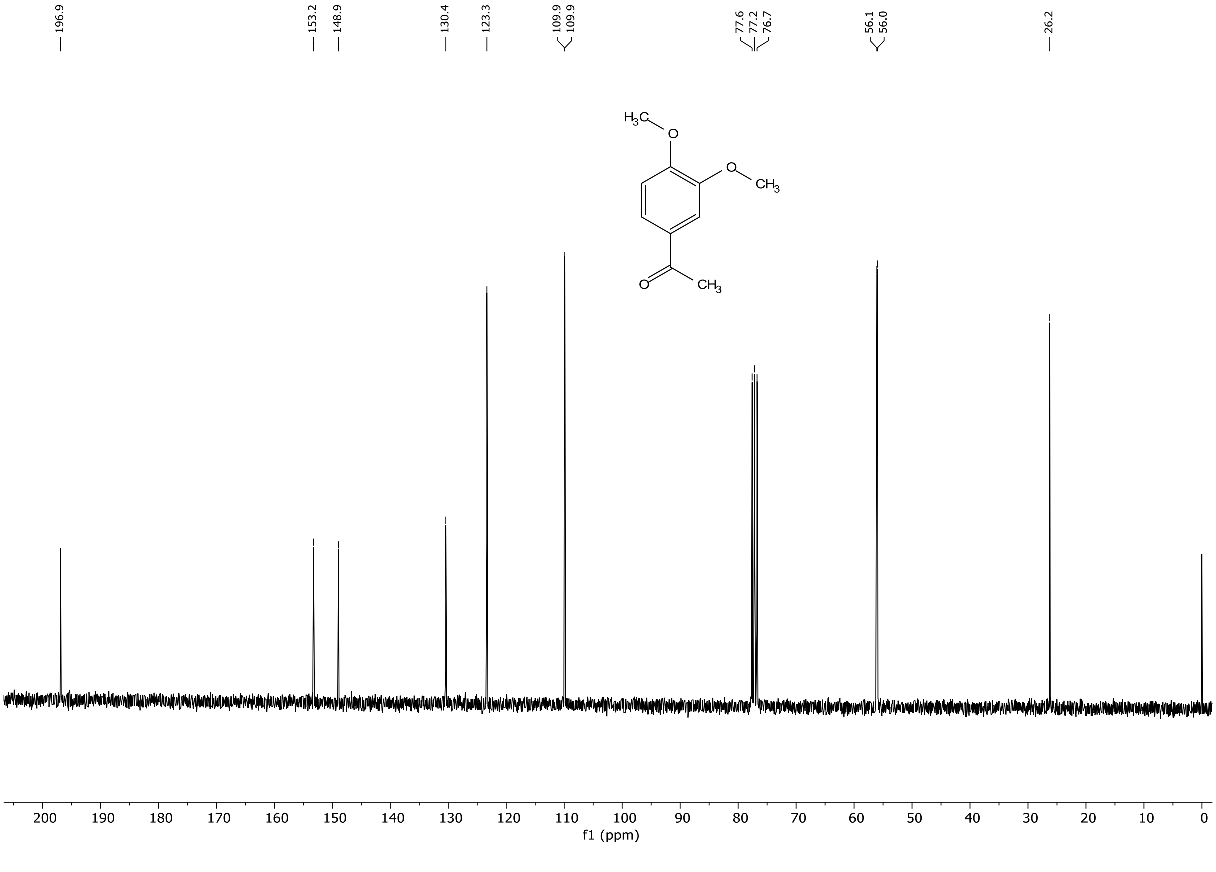
196.9
153.2
148.9
130.4
123.3
109.9
109.9
77.6
77.2
76.7
56.1
56.0
26.2
H3C
O
O
CH3
O
CH3
f1 (ppm)

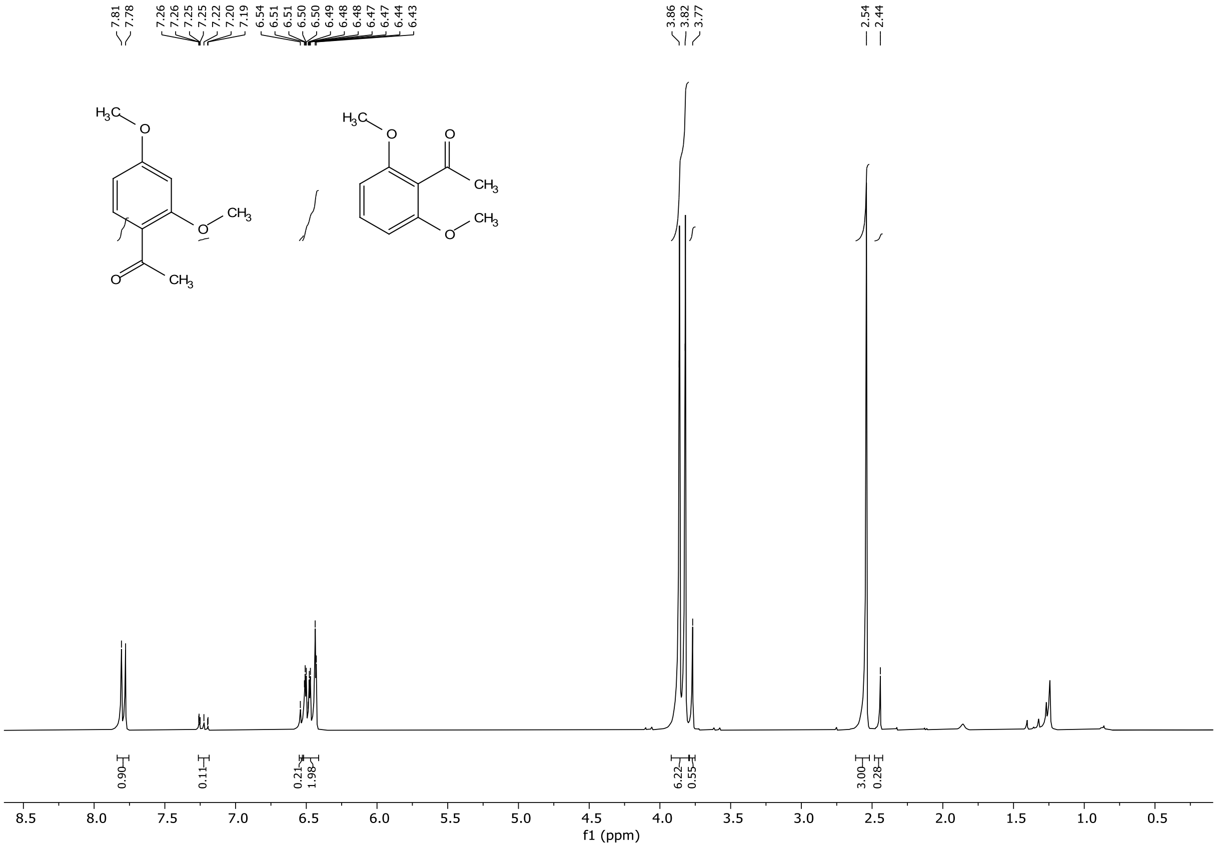
7.81
7.78
7.26
7.26
7.25
7.25
7.22
7.20
7.19
6.54
6.51
6.51
6.50
6.50
6.49
6.48
6.48
6.47
6.47
6.44
6.43
3.86
3.82
3.77
2.54
2.44
H3C
O
CH3
O
CH3
0.90
0.11
0.21
1.98
6.22
0.55
3.00
0.28
8.5
8.0
7.5
7.0
6.5
6.0
5.5
5.0
4.5
4.0
3.5
3.0
2.5
2.0
1.5
1.0
0.5
f1 (ppm)

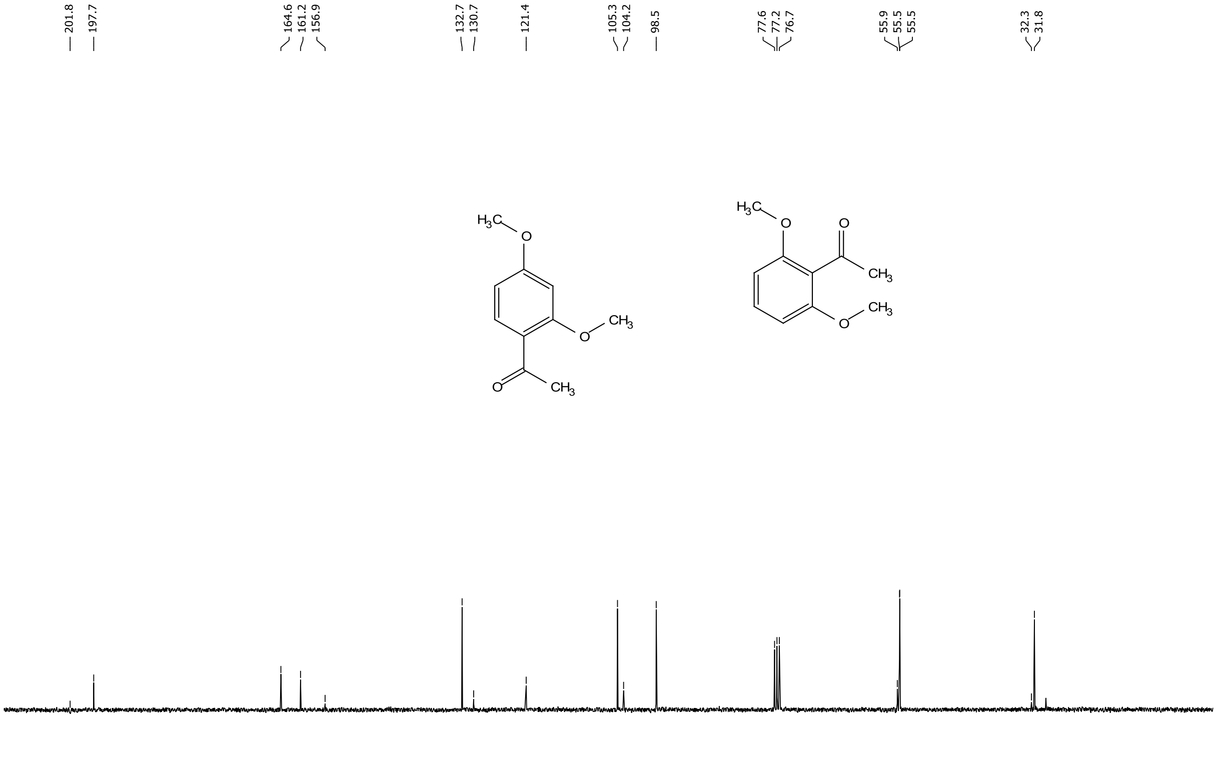
201.8
197.7
164.6
161.2
156.9
132.7
130.7
121.4
105.3
104.2
98.5
77.6
77.2
76.7
55.9
55.5
55.5
32.3
31.8
H3C
O
CH3
O
CH3
210
200
190
180
170
160
150
140
130
120
110
100
90
80
70
60
50
40
30
20
10
f1 (ppm)

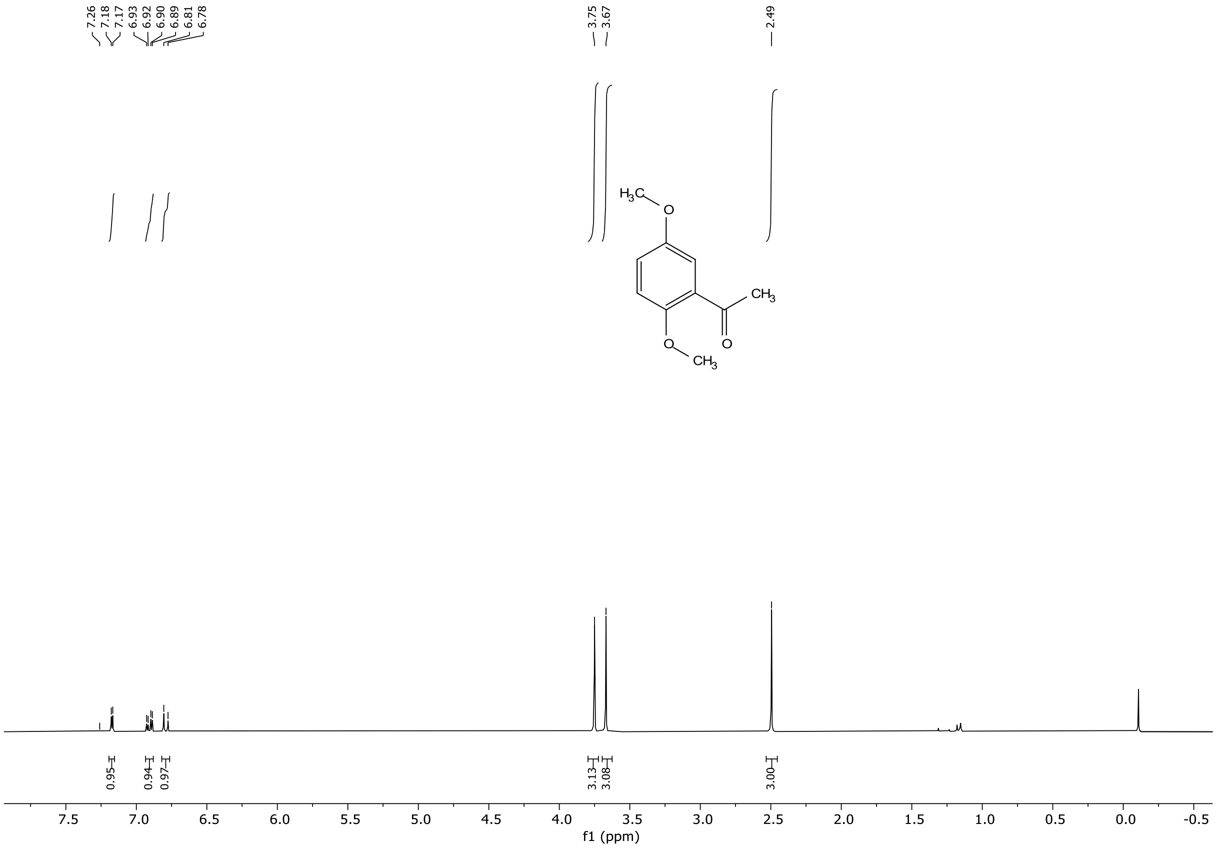
7.26
7.18
7.17
6.93
6.92
6.90
6.89
6.81
6.78
3.75
3.67
2.49
0.95
0.94
0.97
3.13
3.08
3.00
7.5 7.0 6.5 6.0 5.5 5.0 4.5 4.0 3.5 3.0 2.5 2.0 1.5 1.0 0.5 0.0 -0.5
f1 (ppm)

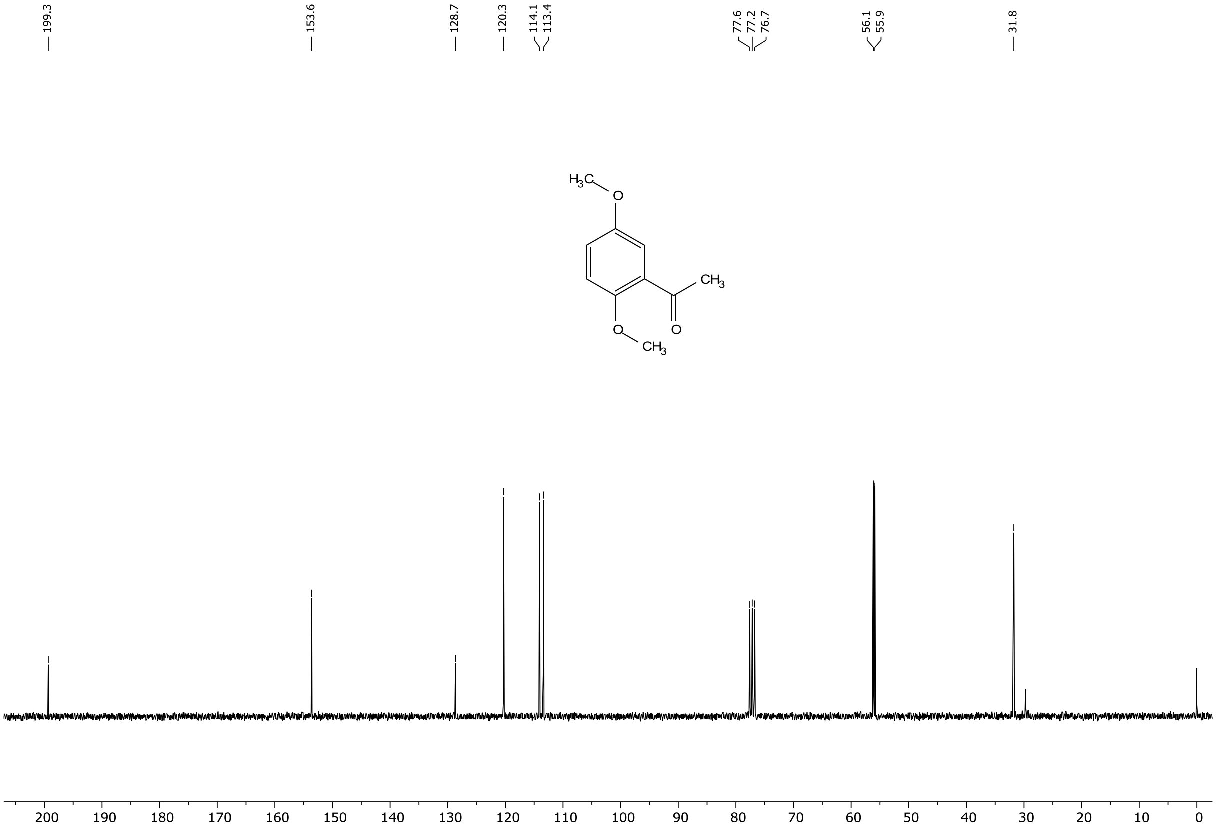
199.3
153.6
128.7
120.3
114.1
113.4
77.6
77.2
76.7
56.1
55.9
31.8
200 190 180 170 160 150 140 130 120 110 100 90 80 70 60 50 40 30 20 10 0
f1 (ppm)

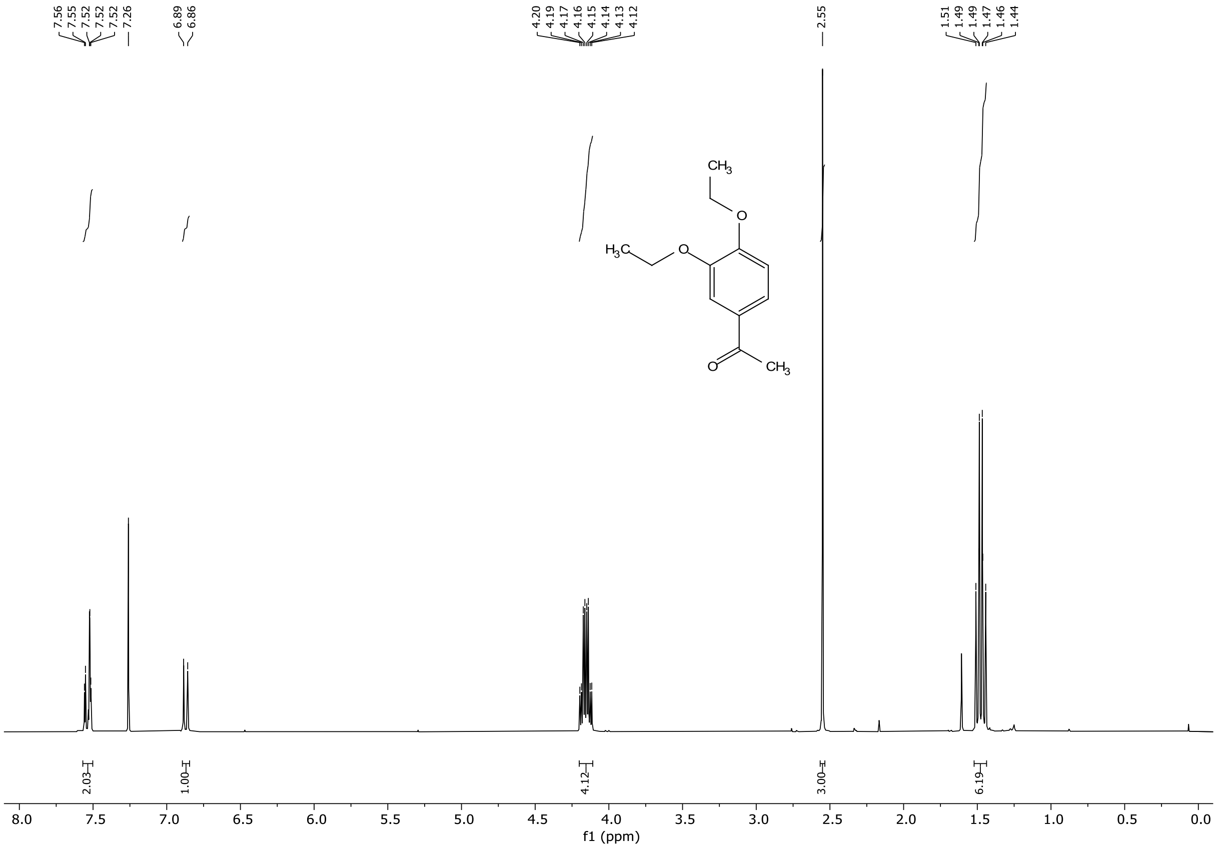
7.56
7.55
7.52
7.52
7.52
7.26
6.89
6.86
4.20
4.19
4.17
4.16
4.15
4.14
4.13
4.12
2.55
1.51
1.49
1.49
1.47
1.46
1.44
CH3
H3C
O
O
O
CH3
2.03
1.00
4.12
3.00
6.19
8.0 7.5 7.0 6.5 6.0 5.5 5.0 4.5 4.0 3.5 3.0 2.5 2.0 1.5 1.0 0.5 0.0
f1 (ppm)

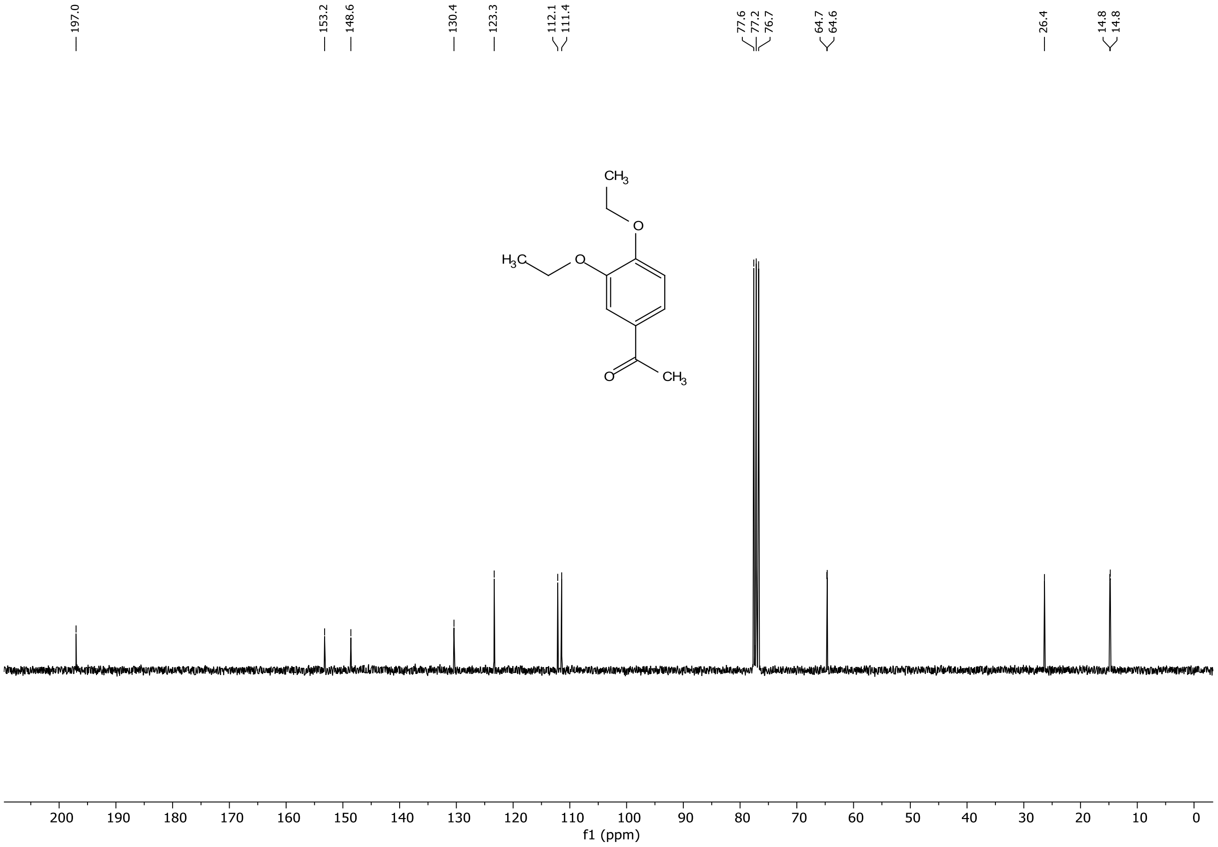
197.0
153.2
148.6
130.4
123.3
112.1
111.4
77.6
77.2
76.7
64.7
64.6
26.4
14.8
14.8
CH3
H3C
O
O
O
CH3
200 190 180 170 160 150 140 130 120 110 100 90 80 70 60 50 40 30 20 10 0
f1 (ppm)

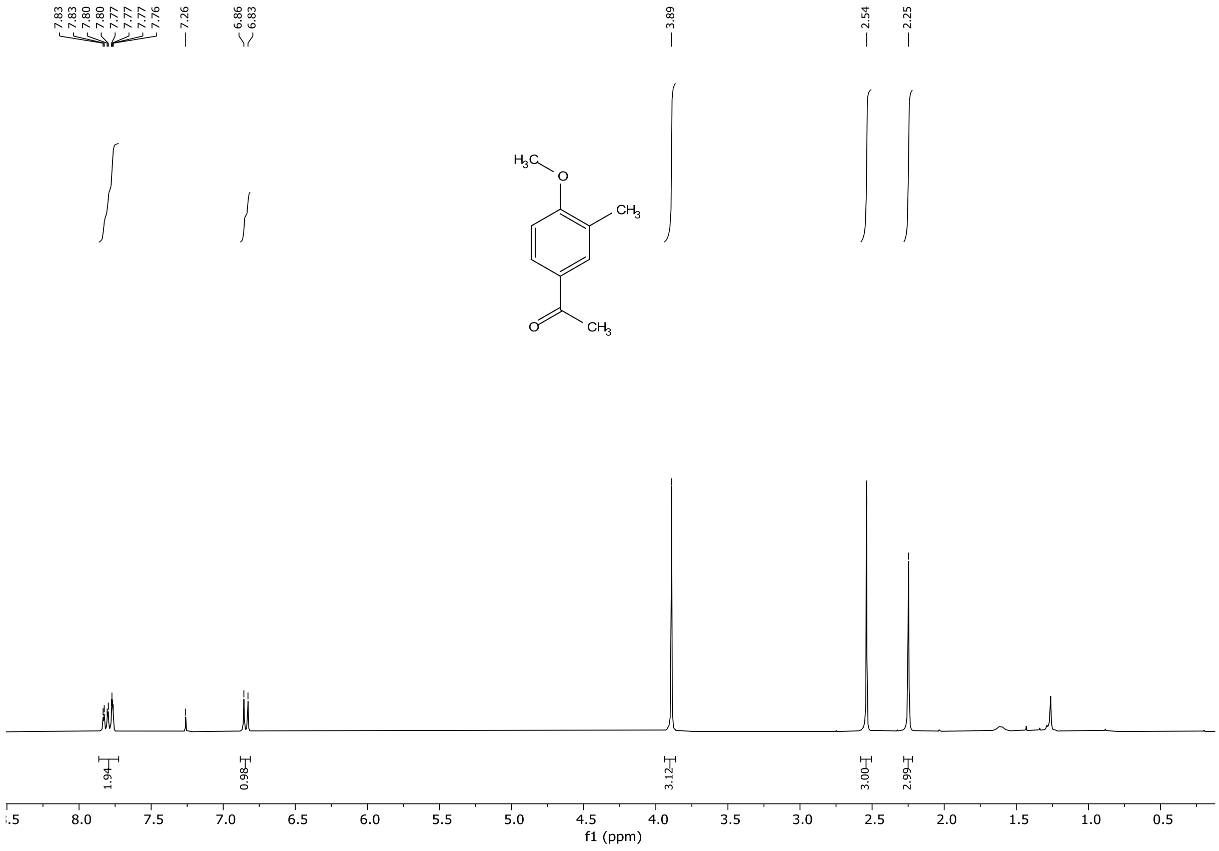
7.83
7.83
7.80
7.80
7.77
7.77
7.77
7.76
7.26
6.86
6.83
3.89
2.54
2.25
H3C
O
CH3
O
CH3
1.94
0.98
3.12
3.00
2.99
8.0
7.5
7.0
6.5
6.0
5.5
5.0
4.5
4.0
3.5
3.0
2.5
2.0
1.5
1.0
0.5
f1 (ppm)

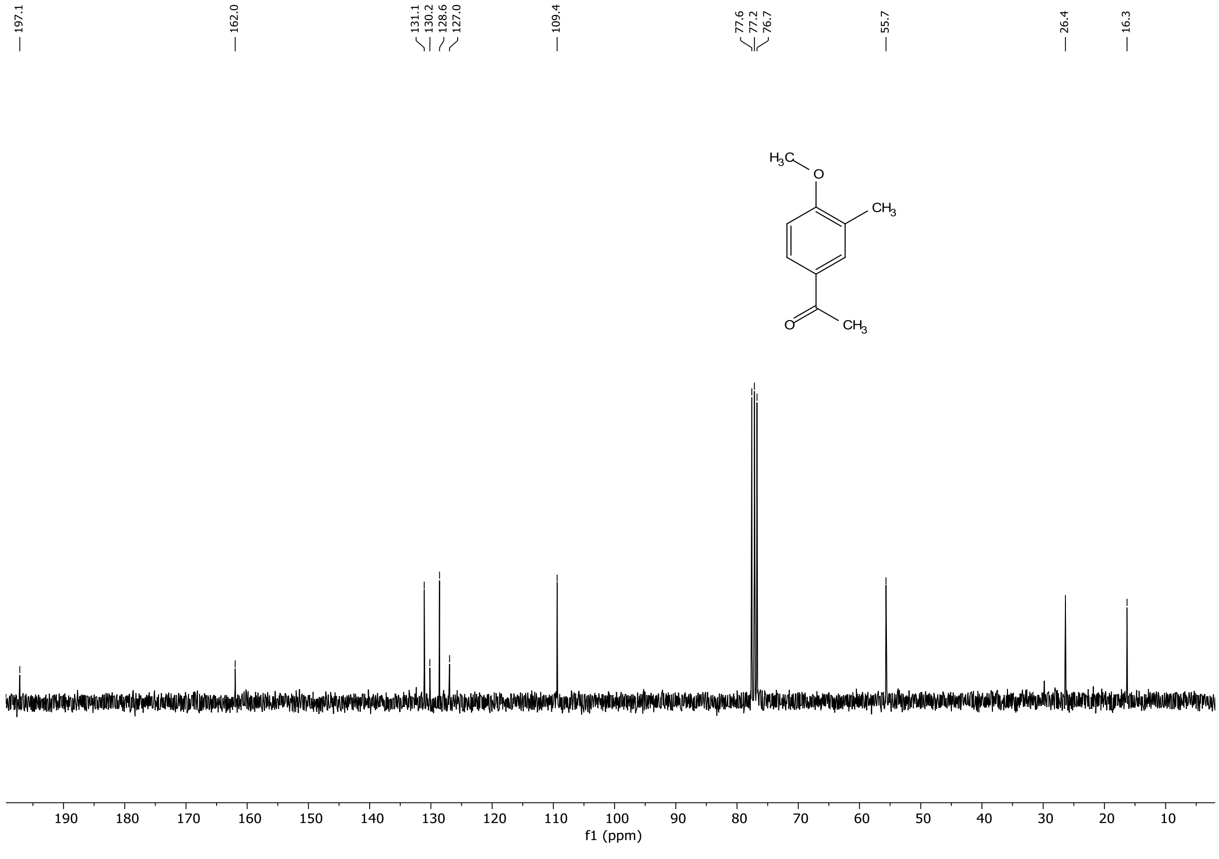
197.1
162.0
131.1
130.2
128.6
127.0
109.4
77.6
77.2
76.7
55.7
26.4
16.3
H3C
O
CH3
O
CH3
190
180
170
160
150
140
130
120
110
100
90
80
70
60
50
40
30
20
10
f1 (ppm)

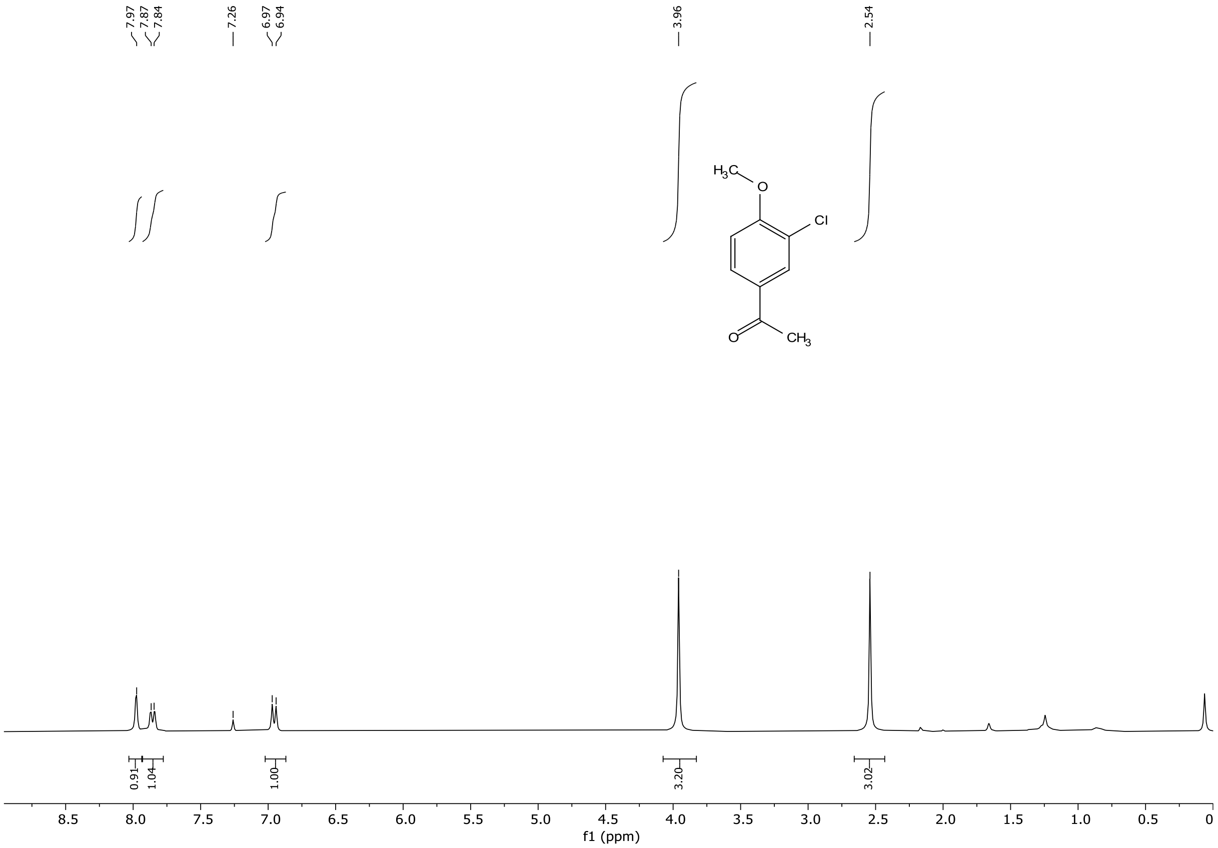
7.97
7.87
7.84
7.26
6.97
6.94
3.96
2.54
H3C
O
Cl
O
CH3
0.91
1.04
1.00
3.20
3.02
8.5 8.0 7.5 7.0 6.5 6.0 5.5 5.0 4.5 4.0 3.5 3.0 2.5 2.0 1.5 1.0 0.5 0
f1 (ppm)

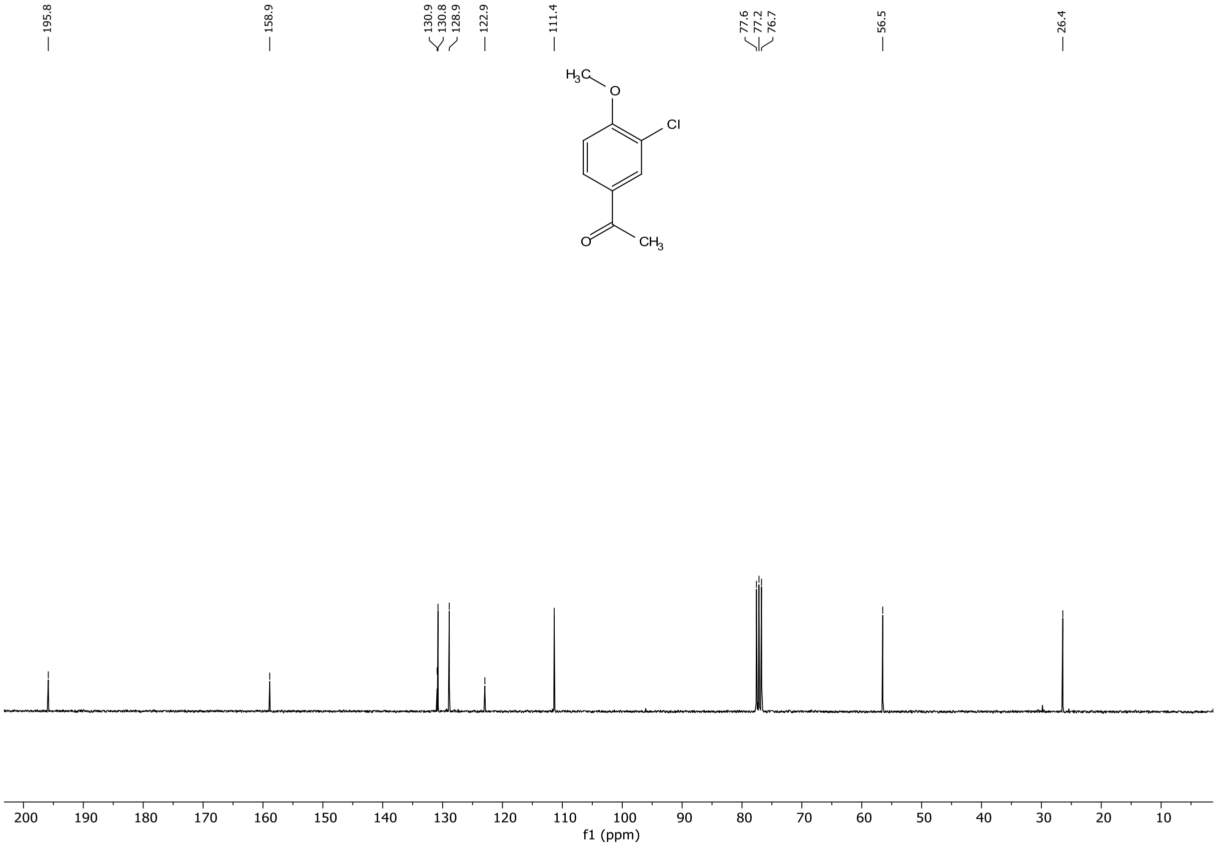
195.8
158.9
130.9
130.8
128.9
122.9
111.4
77.6
77.2
76.7
56.5
26.4
H3C
O
Cl
O
CH3
200 190 180 170 160 150 140 130 120 110 100 90 80 70 60 50 40 30 20 10
f1 (ppm)

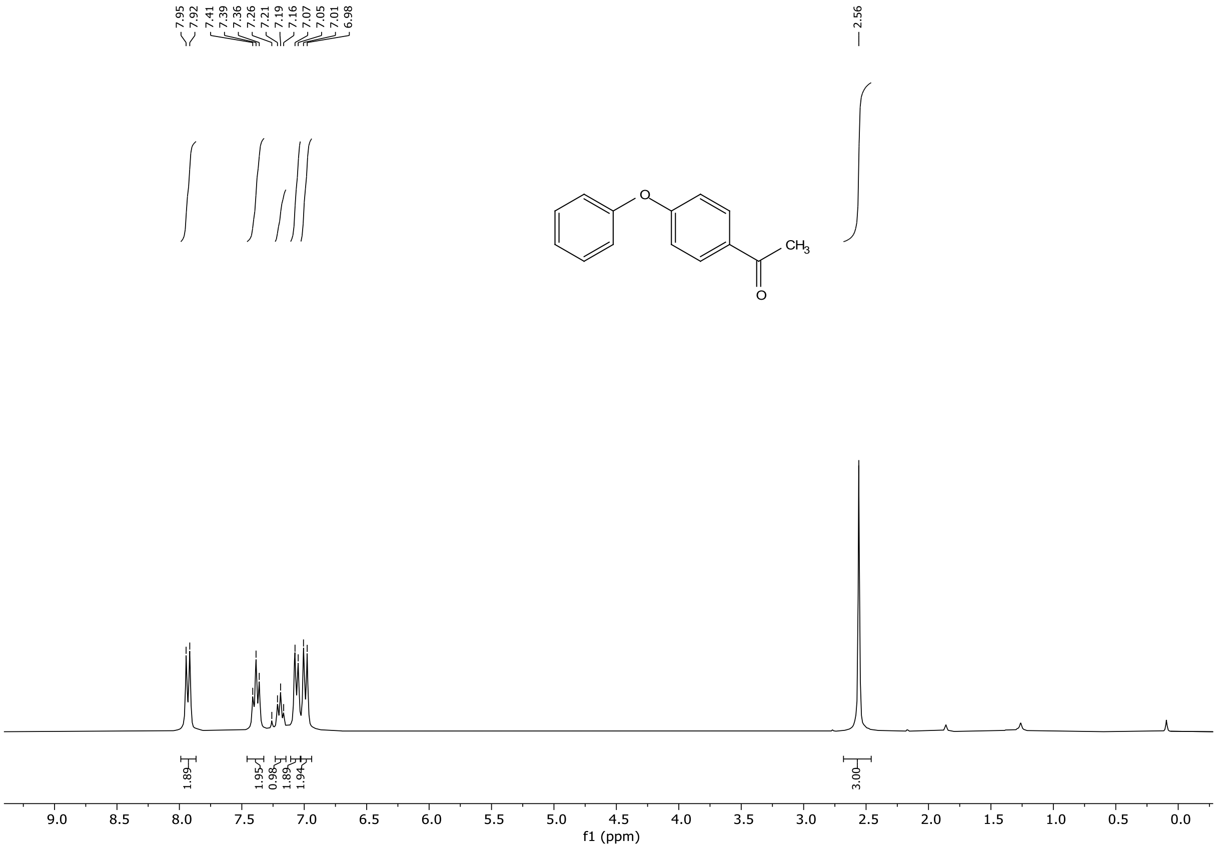
7.95
7.92
7.41
7.39
7.36
7.26
7.21
7.19
7.16
7.07
7.05
7.01
6.98
2.56
O
CH3
O
1.89
1.95
0.98
1.89
1.94
3.00
9.0 8.5 8.0 7.5 7.0 6.5 6.0 5.5 5.0 4.5 4.0 3.5 3.0 2.5 2.0 1.5 1.0 0.5 0.0
f1 (ppm)

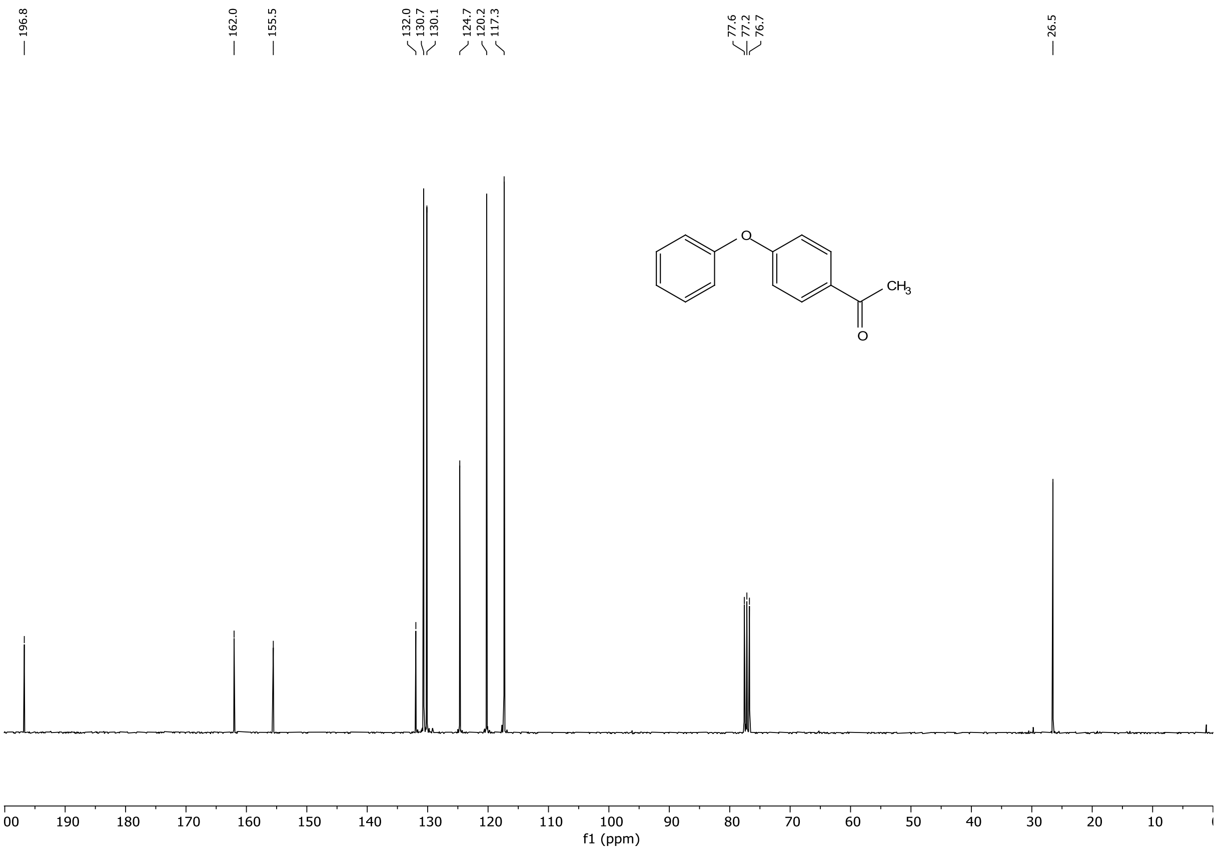
196.8
162.0
155.5
132.0
130.7
130.1
124.7
120.2
117.3
77.6
77.2
76.7
26.5
O
CH3
O
190 180 170 160 150 140 130 120 110 100 90 80 70 60 50 40 30 20 10
f1 (ppm)

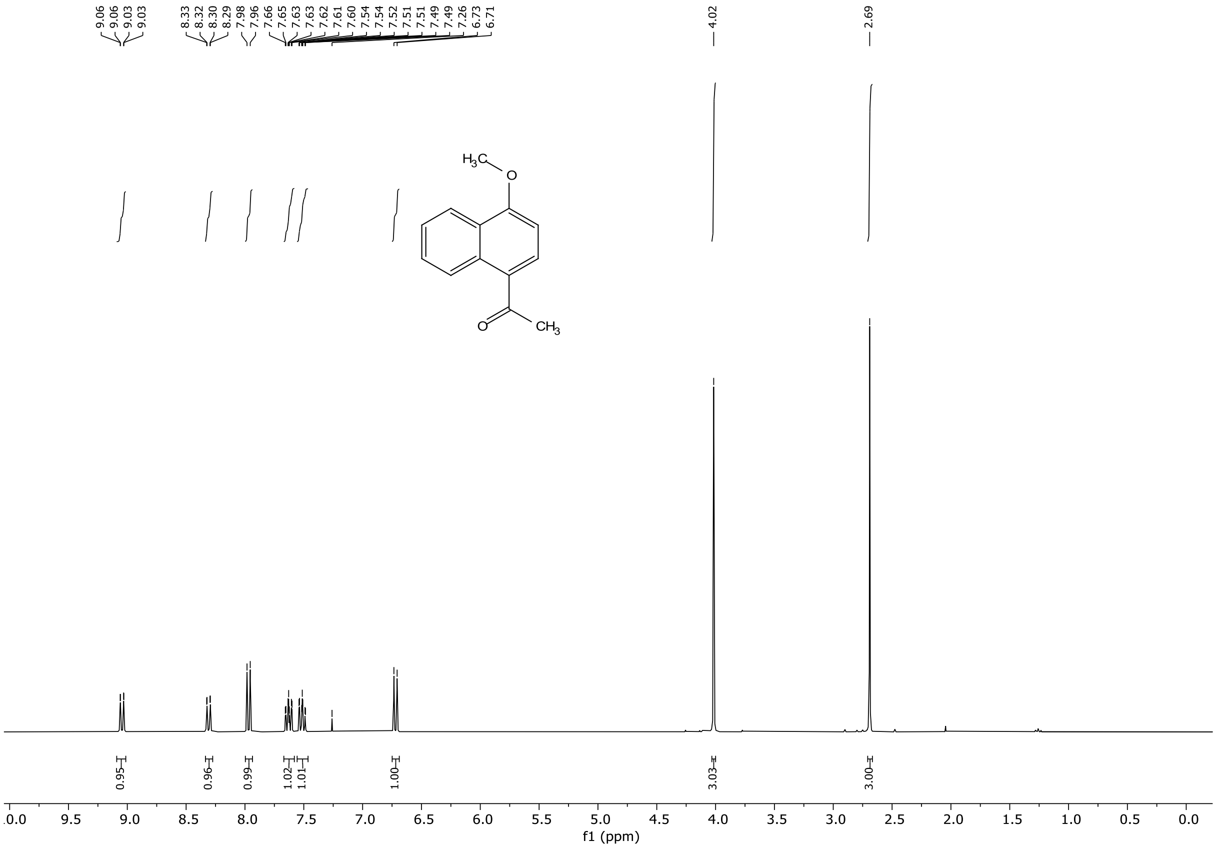
9.06
9.06
9.03
9.03
8.33
8.32
8.30
8.29
7.98
7.96
7.66
7.65
7.63
7.63
7.62
7.61
7.60
7.54
7.54
7.52
7.51
7.51
7.49
7.49
7.26
6.73
6.71
4.02
2.69
H_3C
O
O
CH_3
0.95
0.96
0.99
1.02
1.01
1.00
3.03
3.00
f1 (ppm)

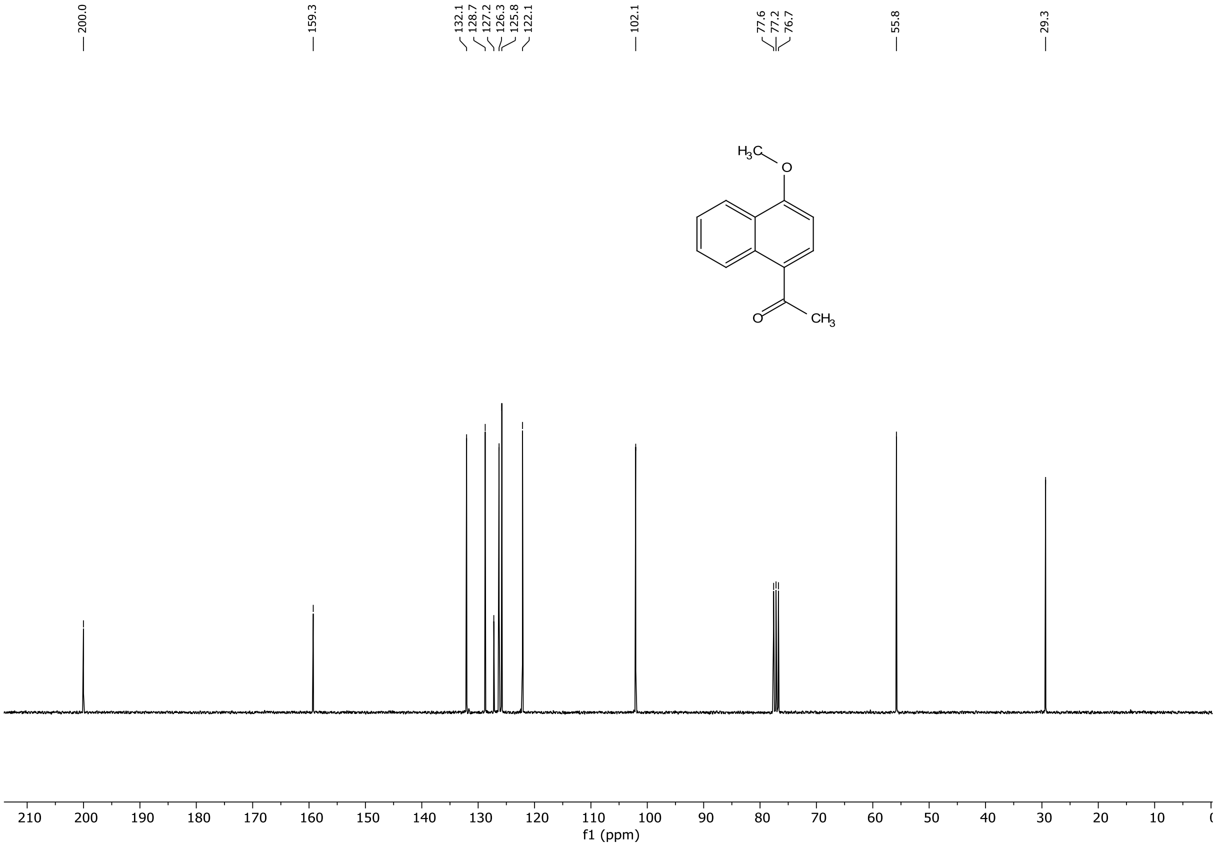
200.0
159.3
132.1
128.7
127.2
126.3
125.8
122.1
102.1
77.6
77.2
76.7
55.8
29.3
H_3C
O
O
CH_3
f1 (ppm)

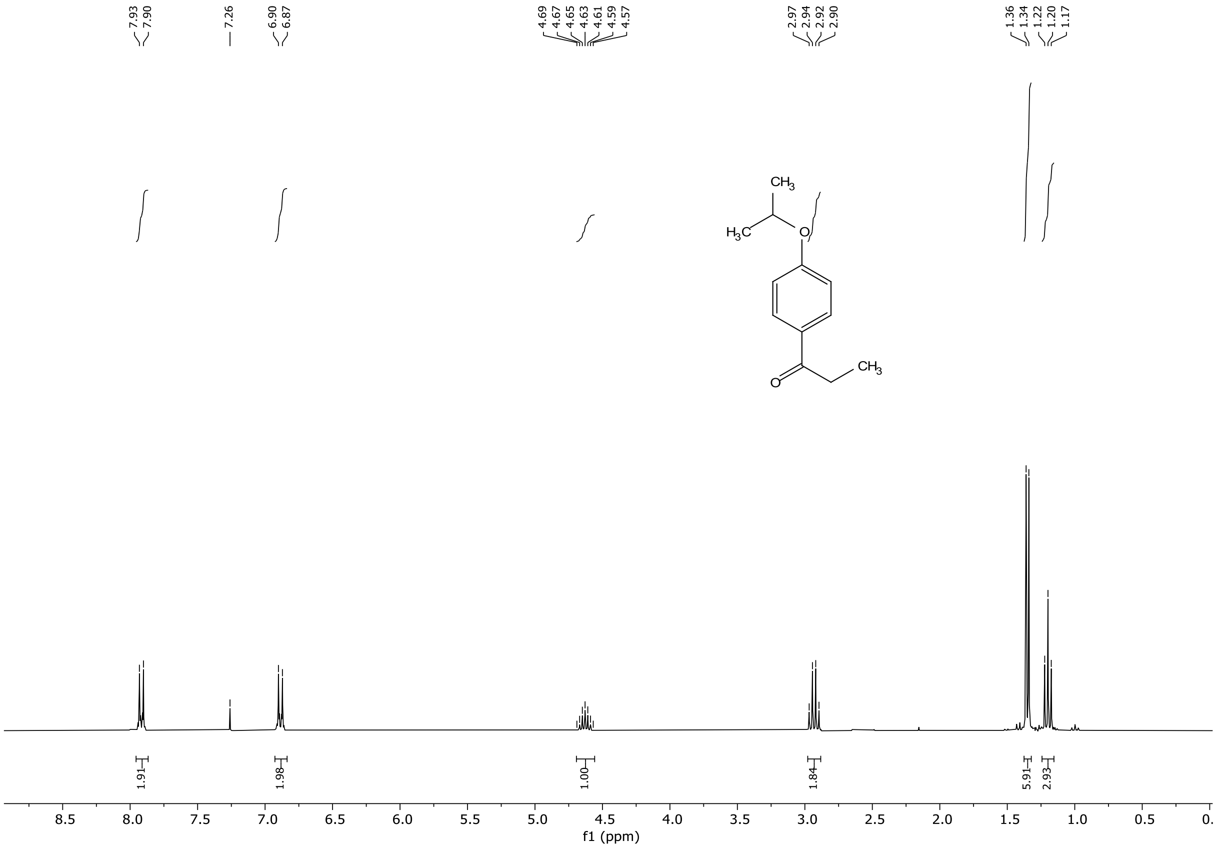
7.93
7.90
7.26
6.90
6.87
4.69
4.67
4.65
4.63
4.61
4.59
4.57
2.97
2.94
2.92
2.90
1.36
1.34
1.22
1.20
1.17
CH3
H3C
O
O
CH3
1.91
1.98
1.00
1.84
5.91
2.93
f1 (ppm)

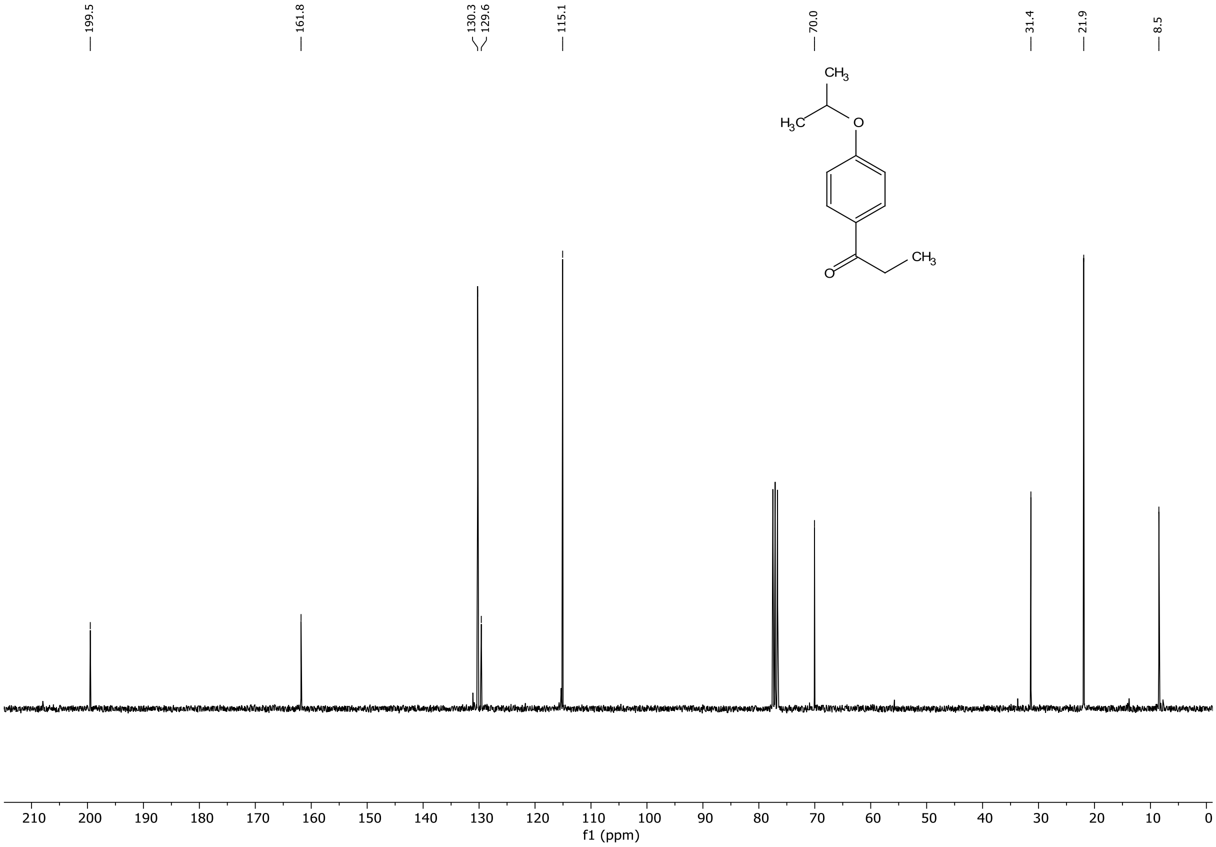
199.5
161.8
130.3
129.6
115.1
70.0
31.4
21.9
8.5
CH3
H3C
O
O
CH3
f1 (ppm)

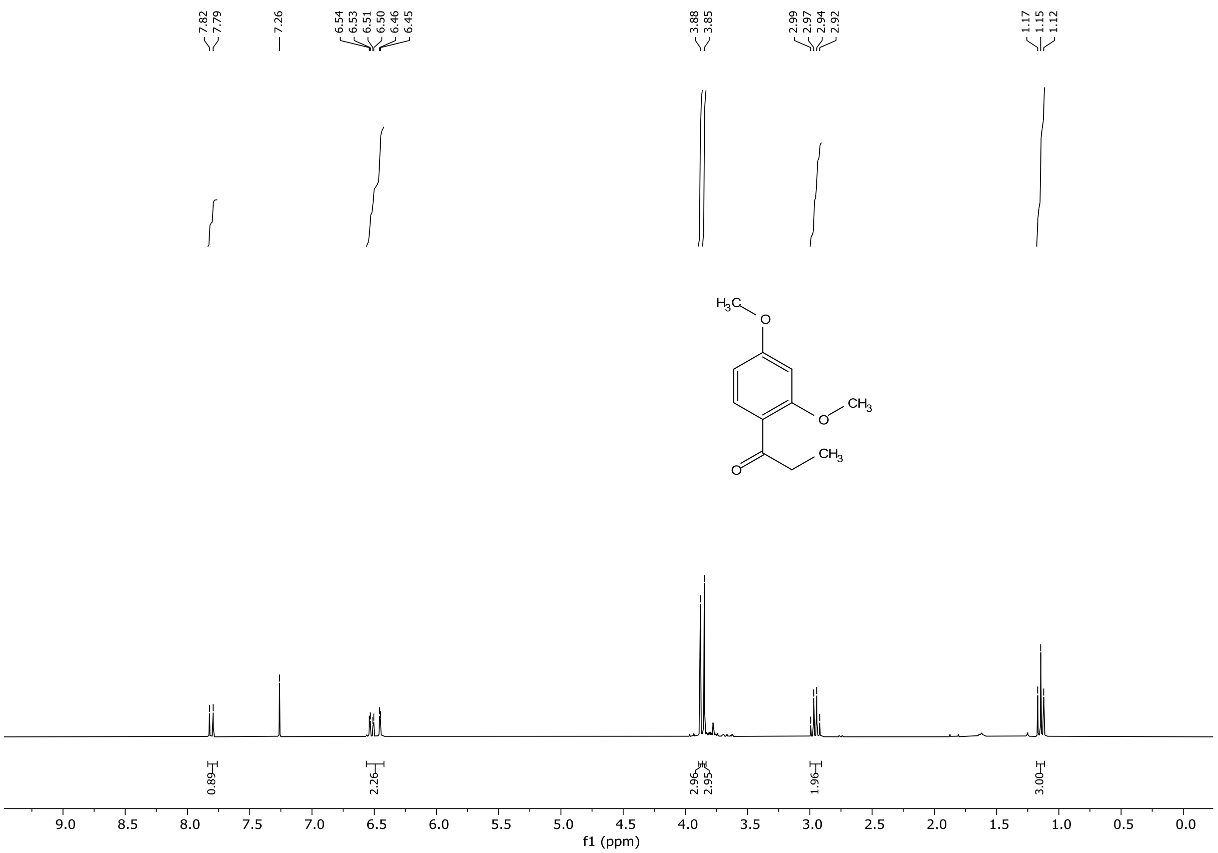

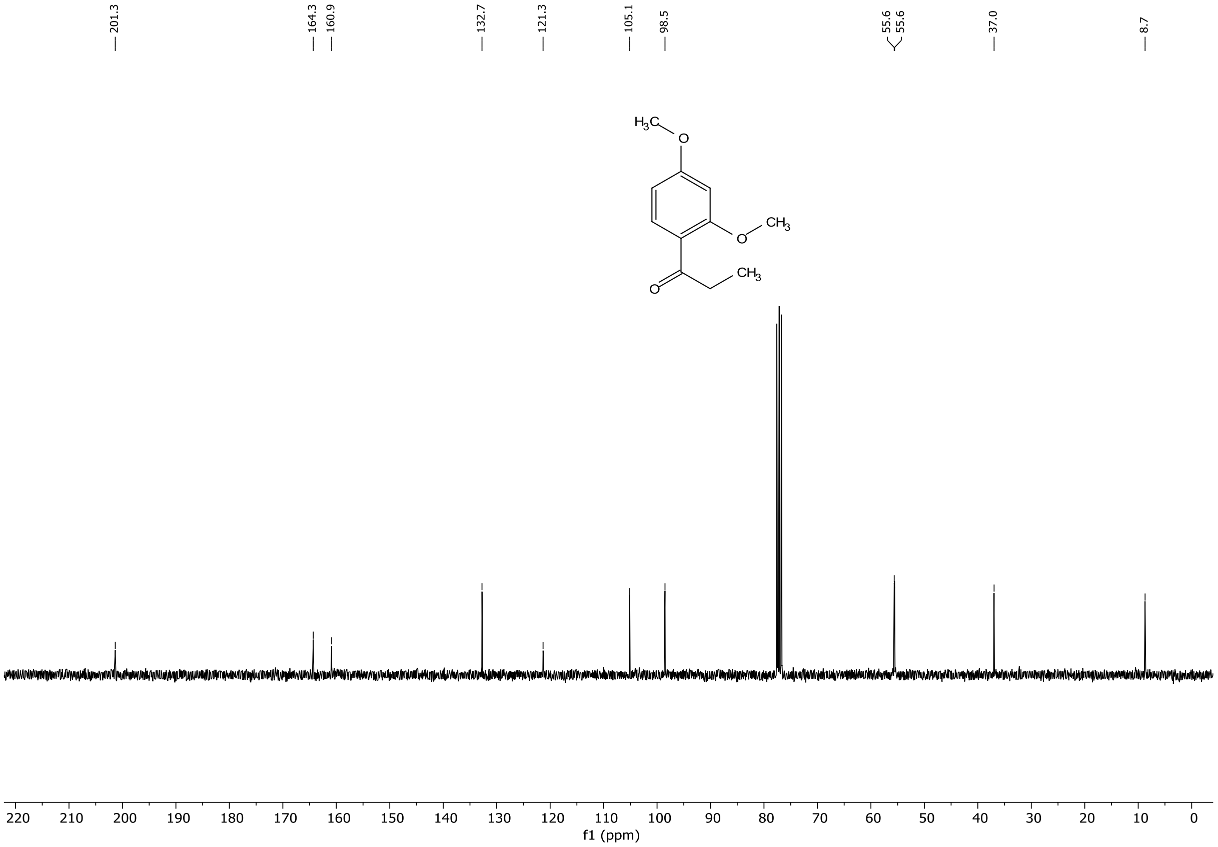

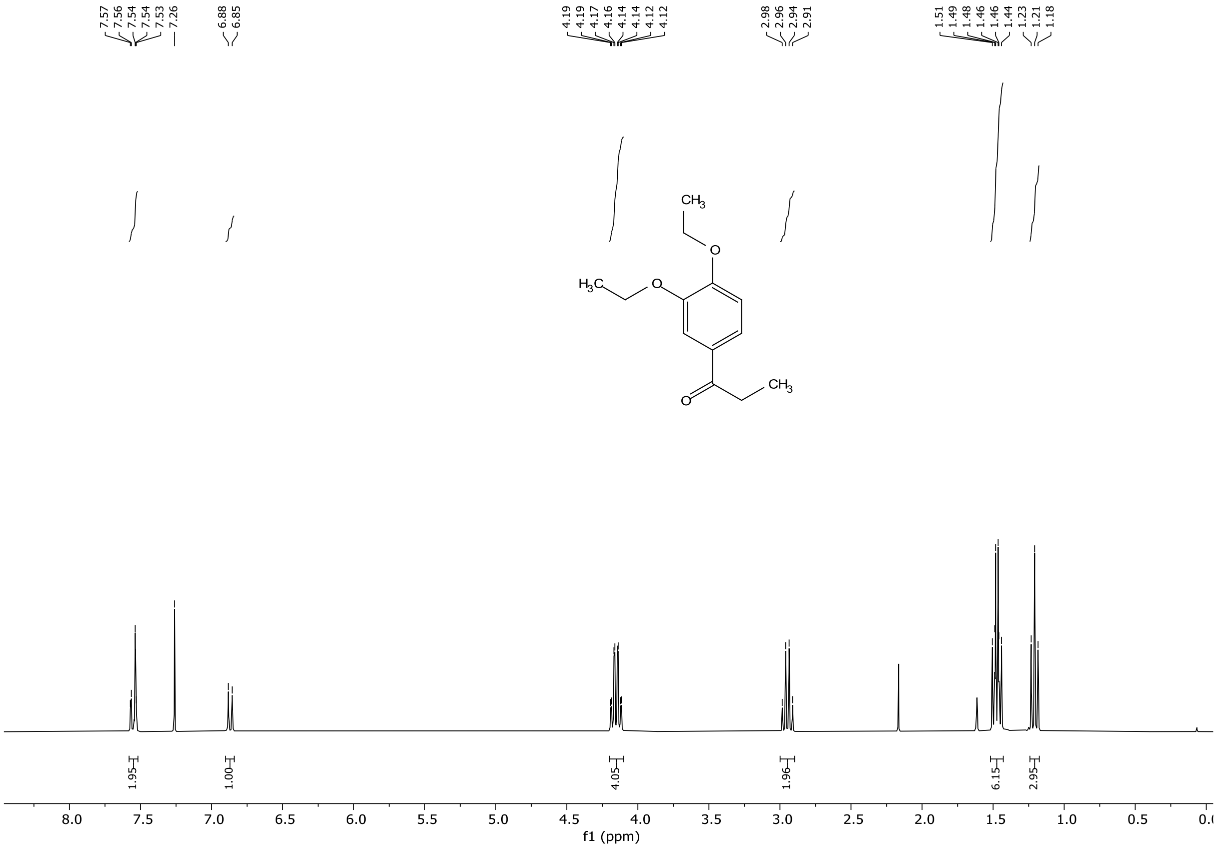
7.57 7.56 7.54 7.54 7.53 7.26
6.88 6.85
4.19 4.19 4.17 4.16 4.14 4.14 4.12 4.12
2.98 2.96 2.94 2.91
1.51 1.49 1.48 1.46 1.46 1.44 1.23 1.21 1.18
CH3
O
H3C
O
O
CH3
1.95
1.00
4.05
1.96
6.15
2.95
8.0 7.5 7.0 6.5 6.0 5.5 5.0 4.5 4.0 3.5 3.0 2.5 2.0 1.5 1.0 0.5
f1 (ppm)

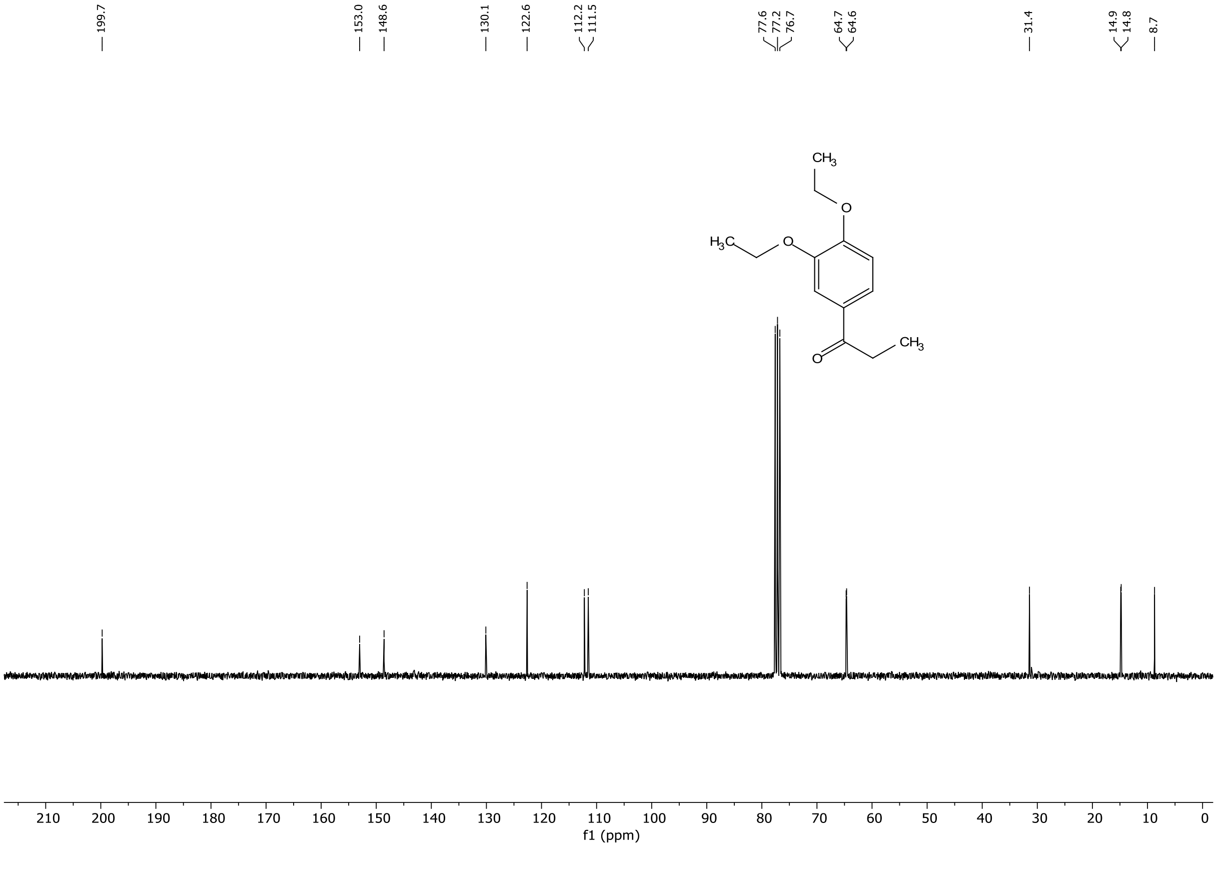
199.7
153.0 148.6
130.1
122.6
112.2 111.5
77.6 77.2 76.7
64.7 64.6
31.4
14.9 14.8
8.7
CH3
O
H3C
O
O
CH3
210 200 190 180 170 160 150 140 130 120 110 100 90 80 70 60 50 40 30 20 10 0
f1 (ppm)

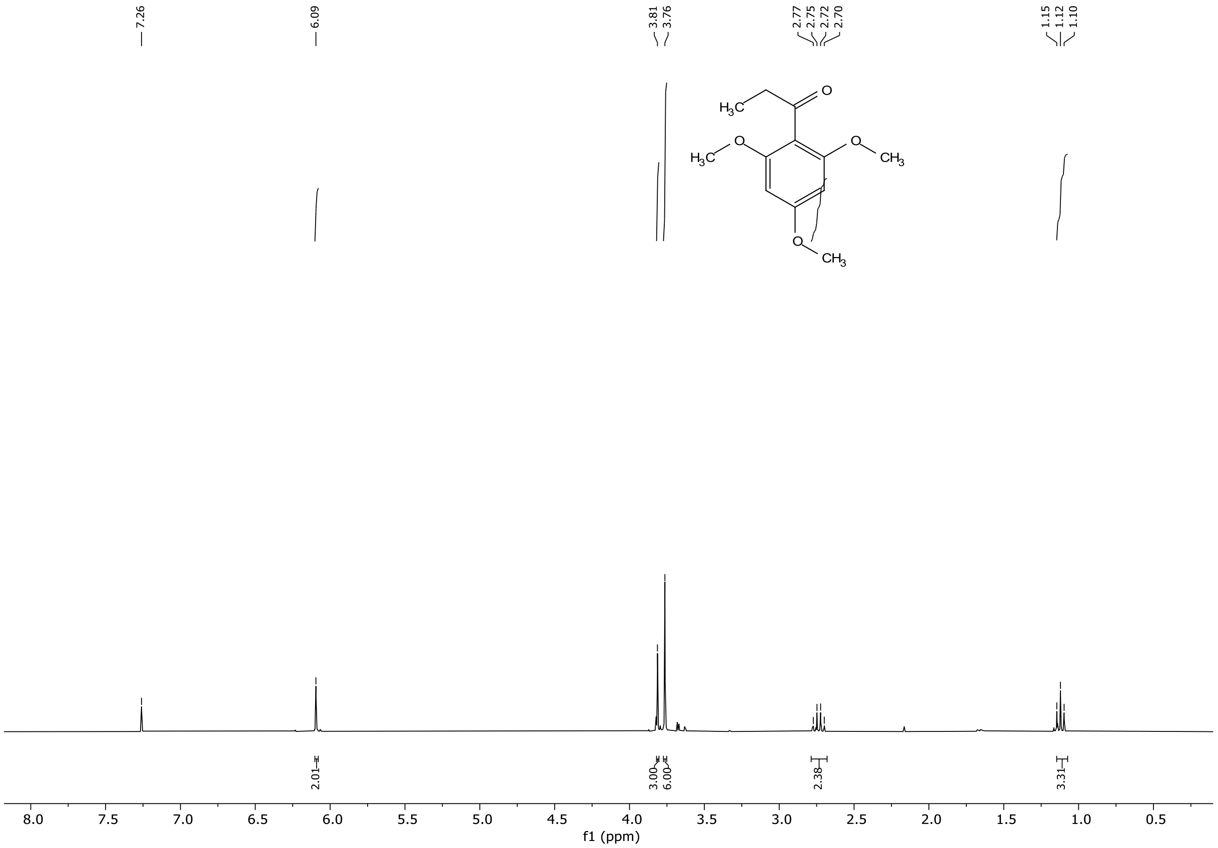
7.26
6.09
3.81
3.76
2.77
2.75
2.72
2.70
1.15
1.12
1.10
2.01
3.00
6.00
2.38
3.31
f1 (ppm)

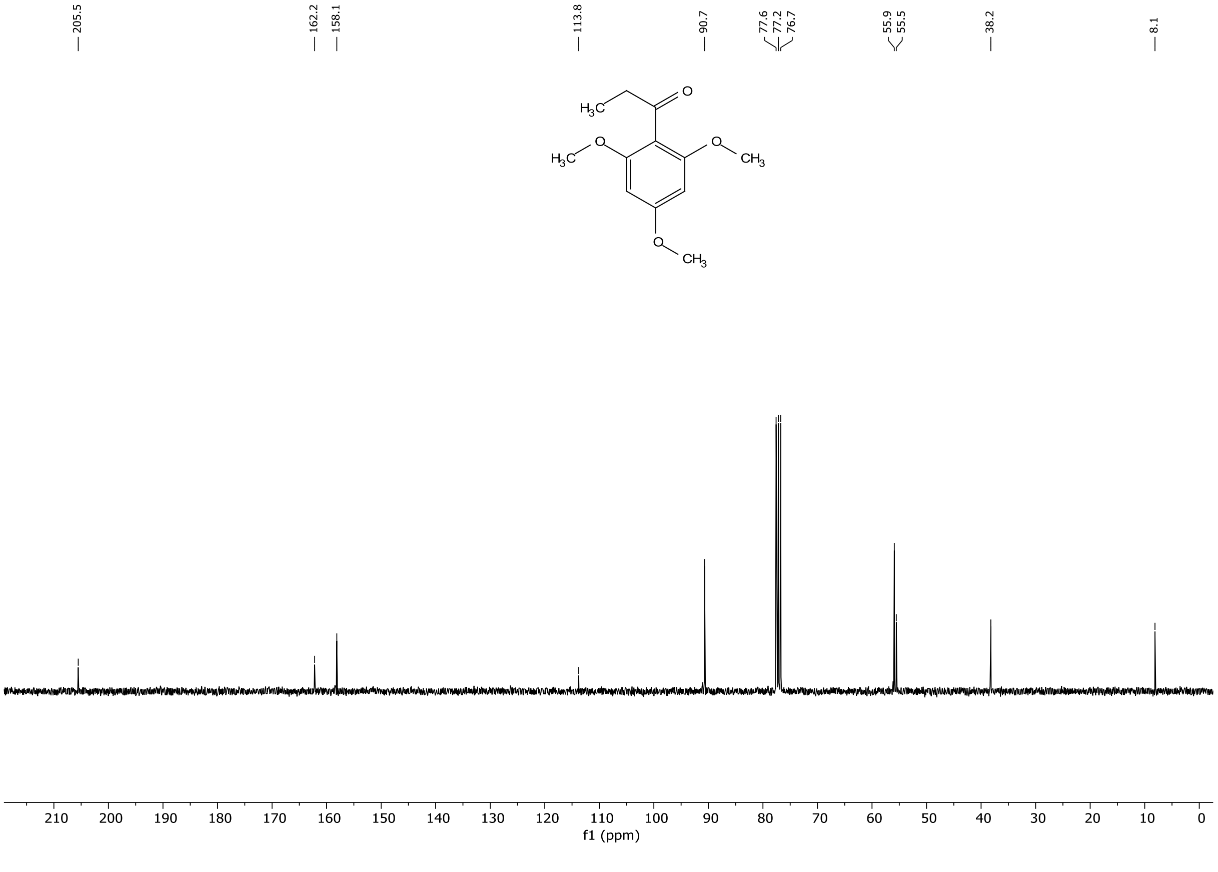
205.5
162.2
158.1
113.8
90.7
77.6
77.2
76.7
55.9
55.5
38.2
8.1
f1 (ppm)

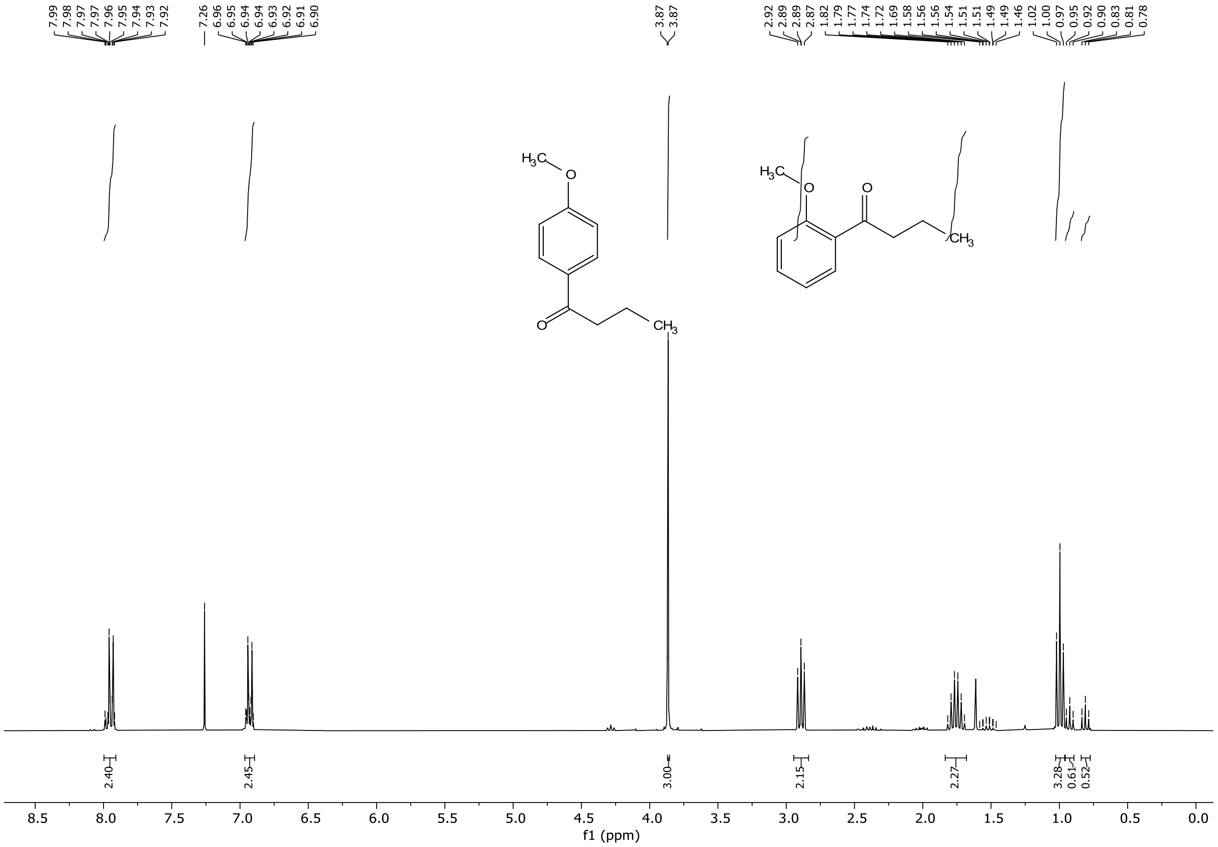
7.99 7.98 7.97 7.97 7.96 7.95 7.94 7.93 7.92
7.26 6.96 6.95 6.94 6.94 6.93 6.92 6.91 6.90
3.87 3.87
2.92 2.89 2.89 2.87 1.82 1.79 1.77 1.74 1.72 1.69 1.58 1.56 1.56 1.54 1.51 1.51 1.49 1.49 1.46 1.02 1.00 0.97 0.95 0.92 0.90 0.83 0.81 0.78
H3C O CH3 O
2.40 2.45 3.00 2.15 2.27 3.28 0.61 0.52
f1 (ppm)

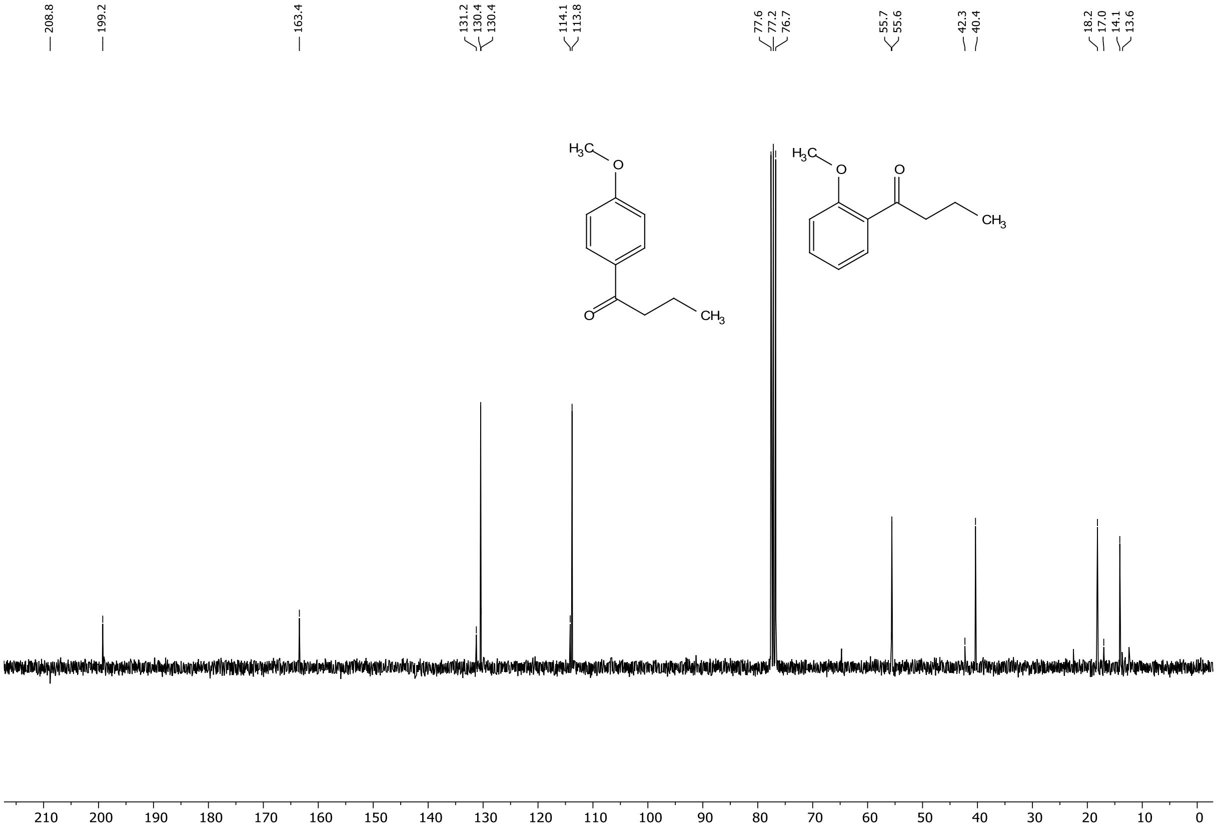
208.8 199.2 163.4 131.2 130.4 130.4 114.1 113.8 77.6 77.2 76.7 55.7 55.6 42.3 40.4 18.2 17.0 14.1 13.6
H3C O CH3 O
f1 (ppm)

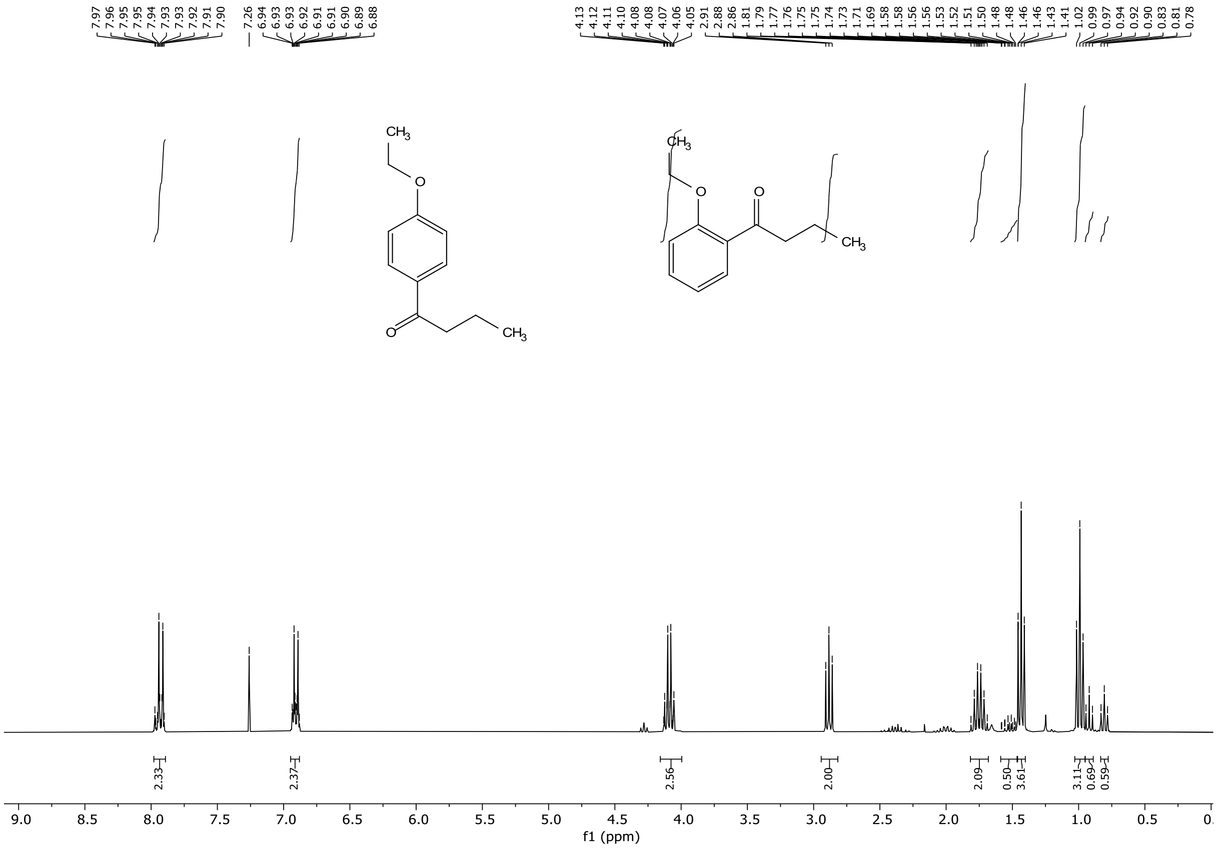
7.97 7.96 7.95 7.95 7.94 7.93 7.93 7.92 7.91 7.90
7.26
6.94 6.93 6.93 6.92 6.91 6.91 6.90 6.89 6.88
4.13 4.12 4.11 4.10 4.08 4.08 4.07 4.06 4.05
2.91 2.88 2.86
1.81 1.79 1.77 1.76 1.75 1.75 1.74 1.73 1.71 1.69 1.58 1.58 1.58 1.56 1.56 1.53 1.52 1.51 1.50 1.48 1.48 1.46 1.46 1.43 1.41
1.02 0.99 0.97 0.94 0.92 0.90 0.83 0.81 0.78
CH3
O
CH3
2.33
2.37
2.56
2.00
2.09
0.50
3.61
3.11
0.69
0.59
9.0 8.5 8.0 7.5 7.0 6.5 6.0 5.5 5.0 4.5 4.0 3.5 3.0 2.5 2.0 1.5 1.0 0.5 0.
f1 (ppm)

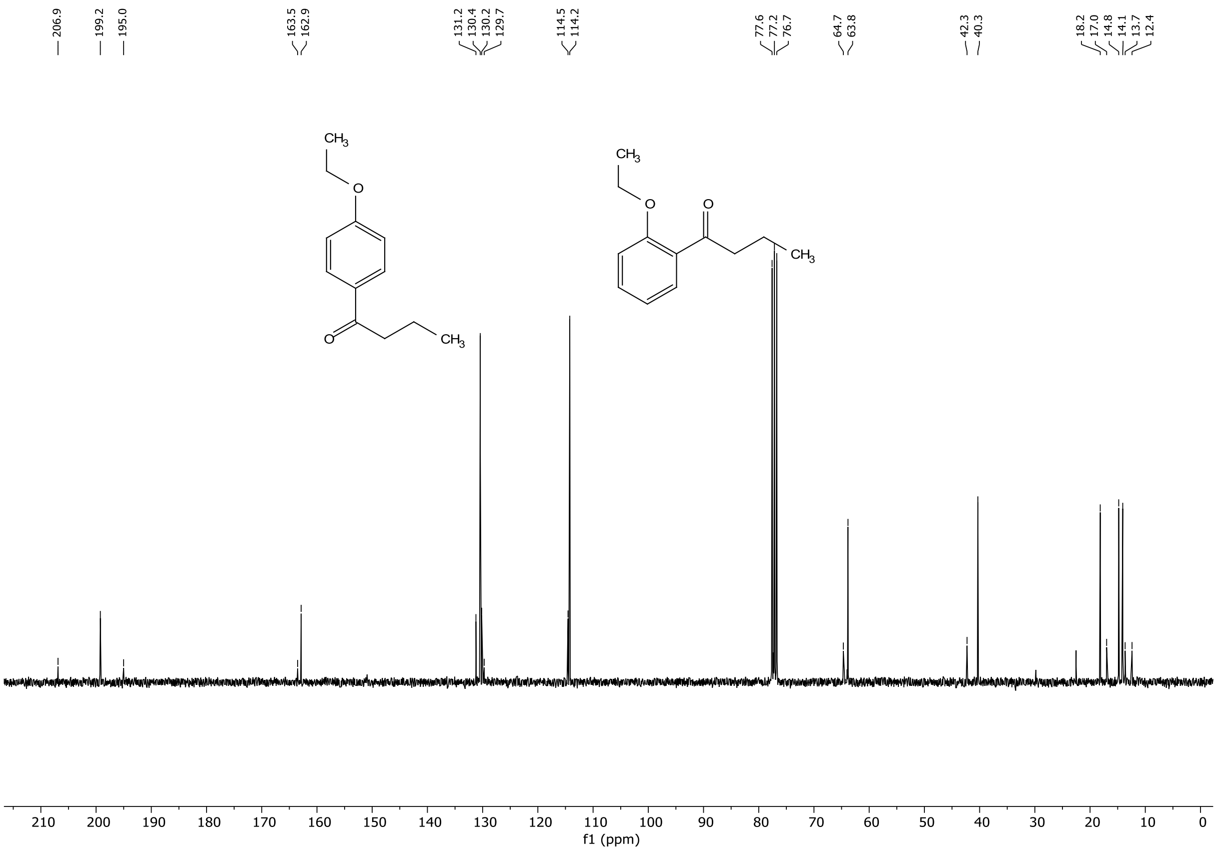
206.9
199.2
195.0
163.5 162.9
131.2 130.4 130.2 129.7
114.5 114.2
77.6 77.2 76.7
64.7 63.8
42.3 40.3
18.2 17.0 14.8 14.1 13.7 12.4
CH3
O
CH3
210 200 190 180 170 160 150 140 130 120 110 100 90 80 70 60 50 40 30 20 10 0
f1 (ppm)

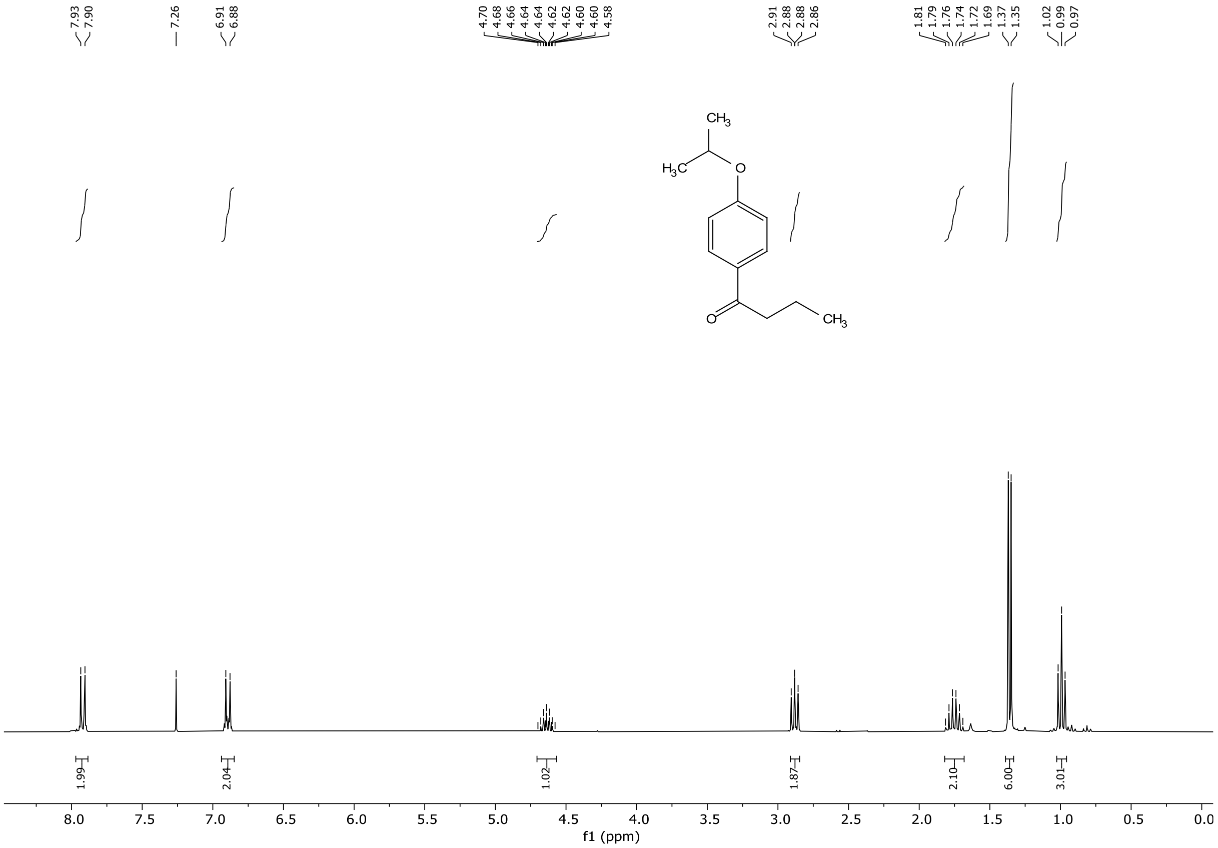
7.93
7.90
7.26
6.91
6.88
4.70
4.68
4.66
4.64
4.64
4.62
4.62
4.60
4.60
4.58
2.91
2.88
2.88
2.86
1.81
1.79
1.76
1.74
1.72
1.69
1.37
1.35
1.02
0.99
0.97
CH3
H3C
O
O
CH3
1.99
2.04
1.02
1.87
2.10
6.00
3.01
f1 (ppm)

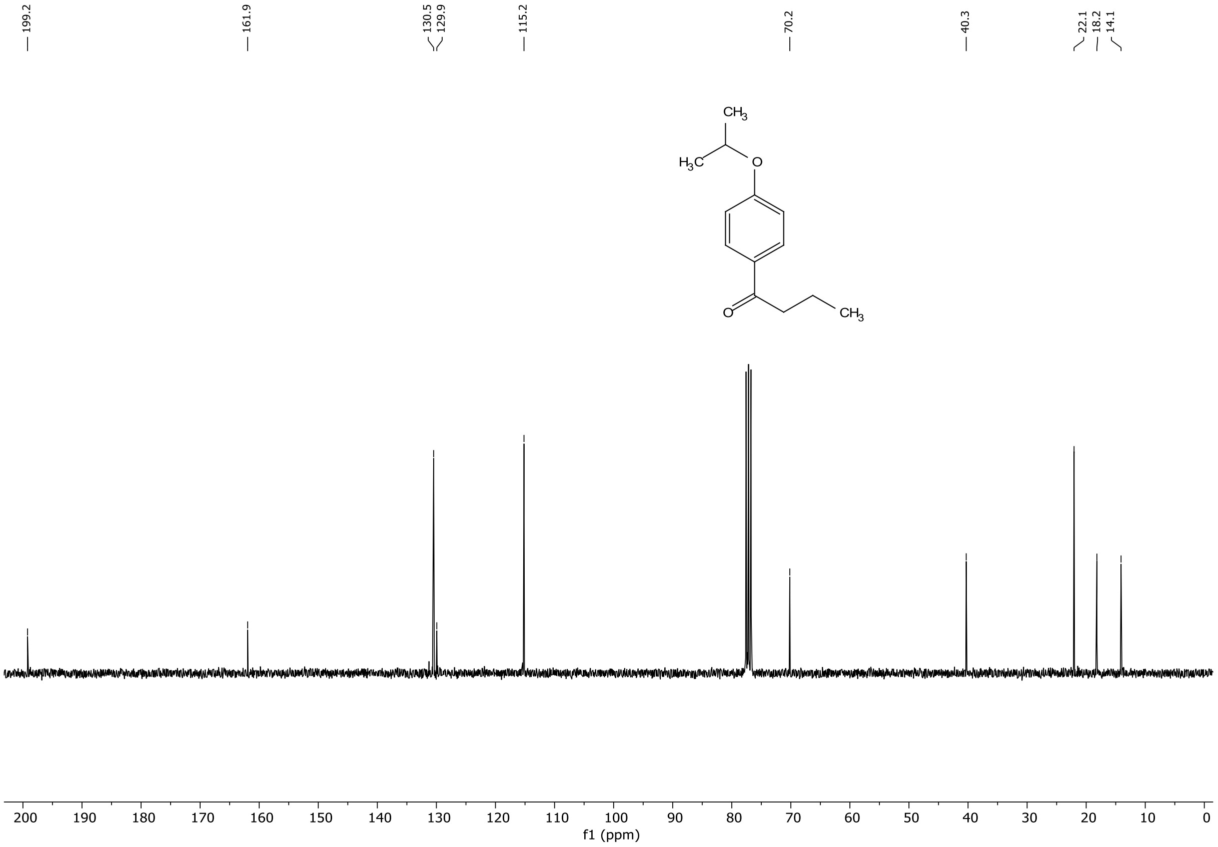
199.2
161.9
130.5
129.9
115.2
70.2
40.3
22.1
18.2
14.1
CH3
H3C
O
O
CH3
f1 (ppm)

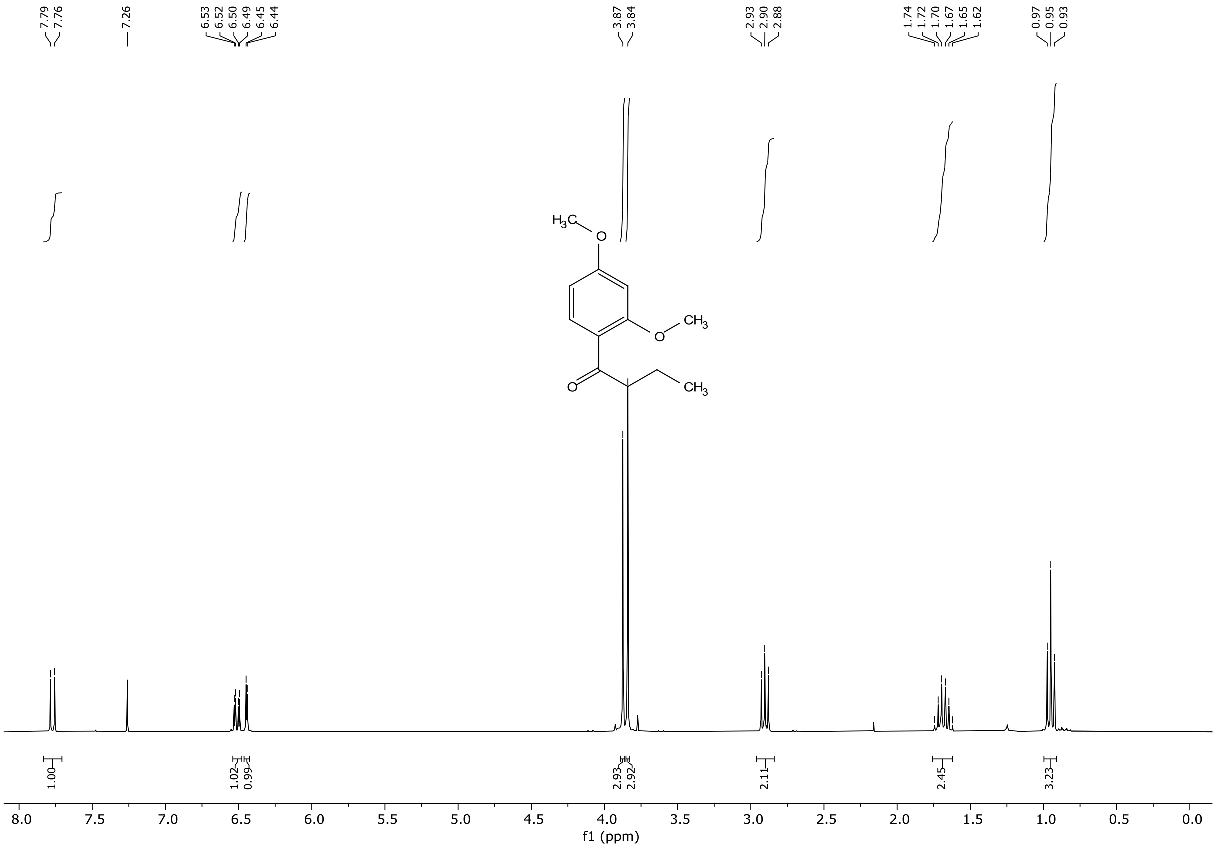
7.79
7.76
7.26
6.53
6.52
6.50
6.49
6.45
6.44
3.87
3.84
2.93
2.90
2.88
1.74
1.72
1.70
1.67
1.65
1.62
0.97
0.95
0.93
H3C
O
O
CH3
O
CH3
1.00
1.02
0.99
2.93
2.92
2.11
2.45
3.23
8.0
7.5
7.0
6.5
6.0
5.5
5.0
4.5
4.0
3.5
3.0
2.5
2.0
1.5
1.0
0.5
0.0
f1 (ppm)

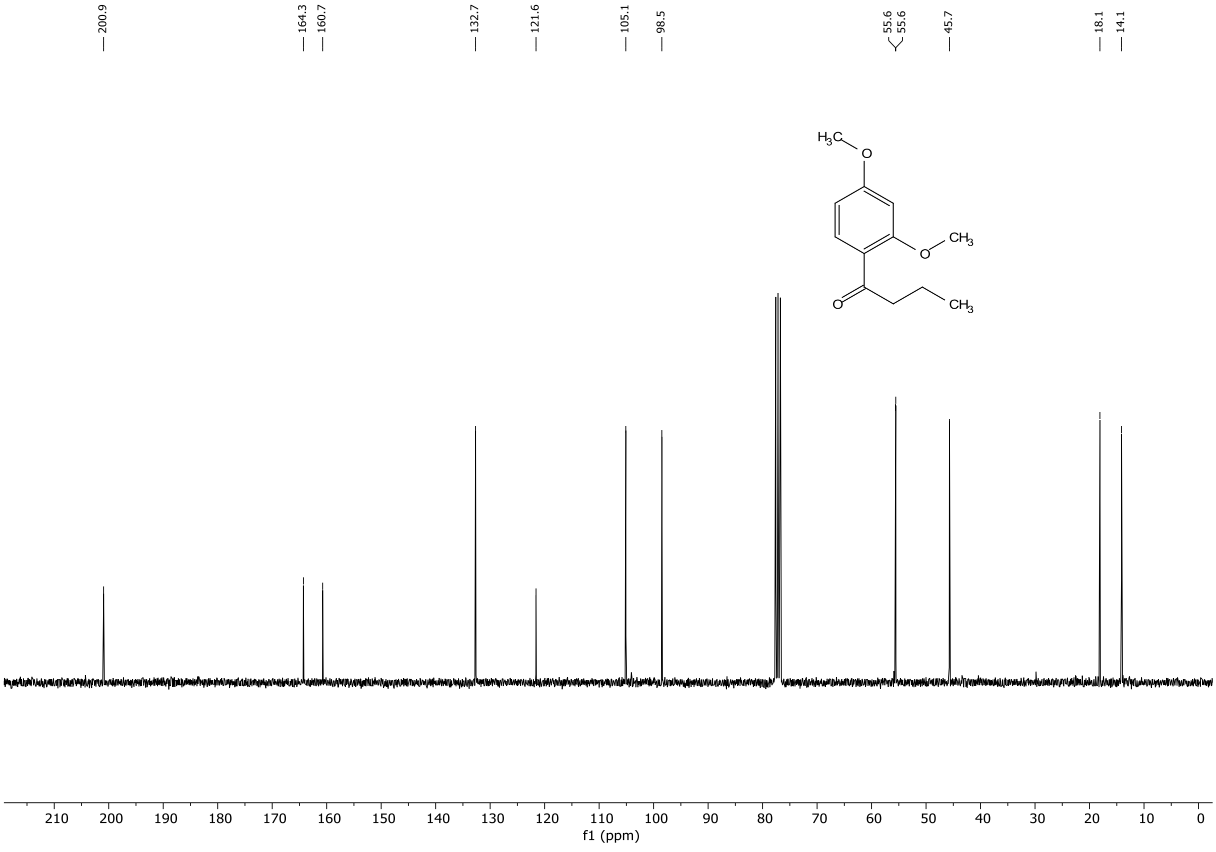
200.9
164.3
160.7
132.7
121.6
105.1
98.5
55.6
55.6
45.7
18.1
14.1
H3C
O
O
CH3
O
CH3
210
200
190
180
170
160
150
140
130
120
110
100
90
80
70
60
50
40
30
20
10
0
f1 (ppm)

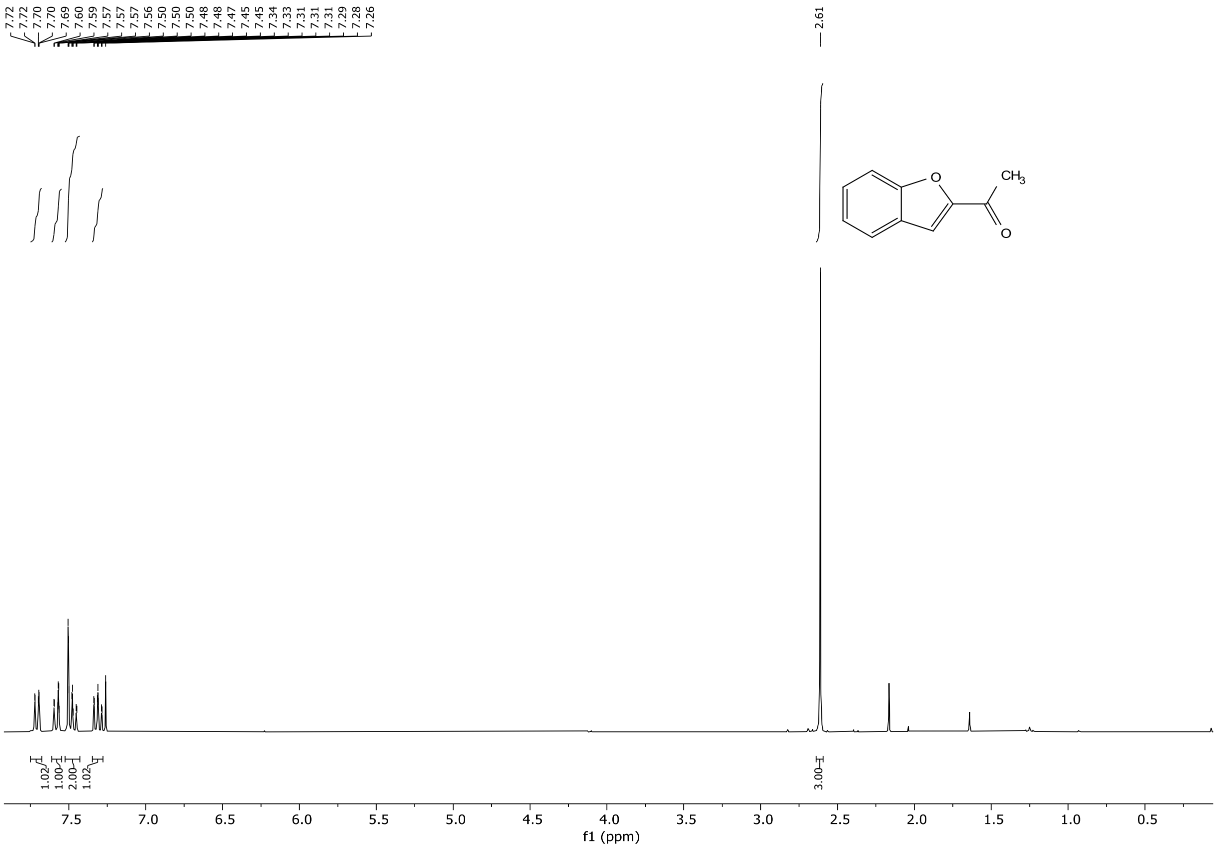
7.72
7.72
7.70
7.70
7.69
7.60
7.59
7.57
7.57
7.57
7.56
7.50
7.50
7.50
7.48
7.48
7.47
7.45
7.45
7.34
7.33
7.31
7.31
7.31
7.29
7.28
7.26
2.61
O
CH3
O
1.02
1.00
2.00
1.02
3.00
7.5
7.0
6.5
6.0
5.5
5.0
4.5
4.0
3.5
3.0
2.5
2.0
1.5
1.0
0.5
f1 (ppm)

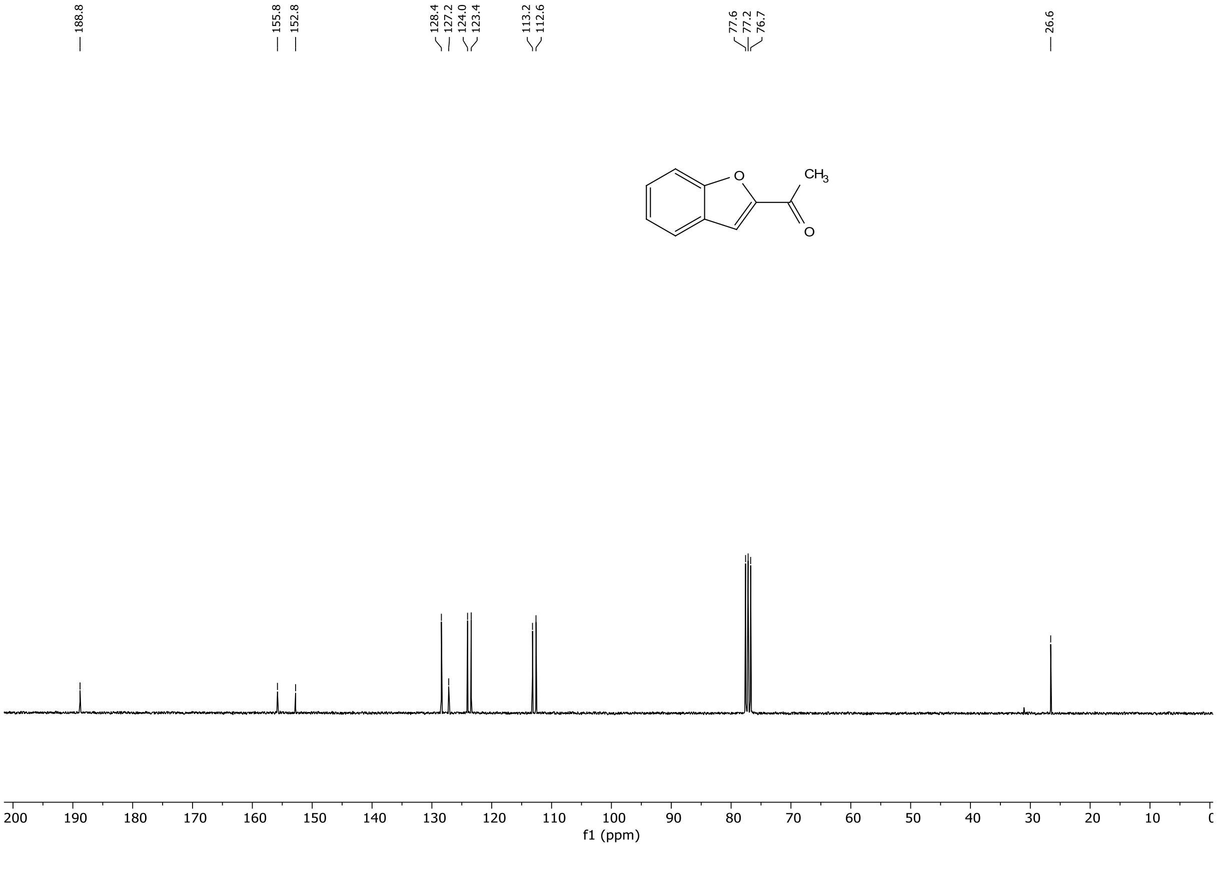
188.8
155.8
152.8
128.4
127.2
124.0
123.4
113.2
112.6
77.6
77.2
76.7
26.6
O
CH3
O
200
190
180
170
160
150
140
130
120
110
100
90
80
70
60
50
40
30
20
10
f1 (ppm)

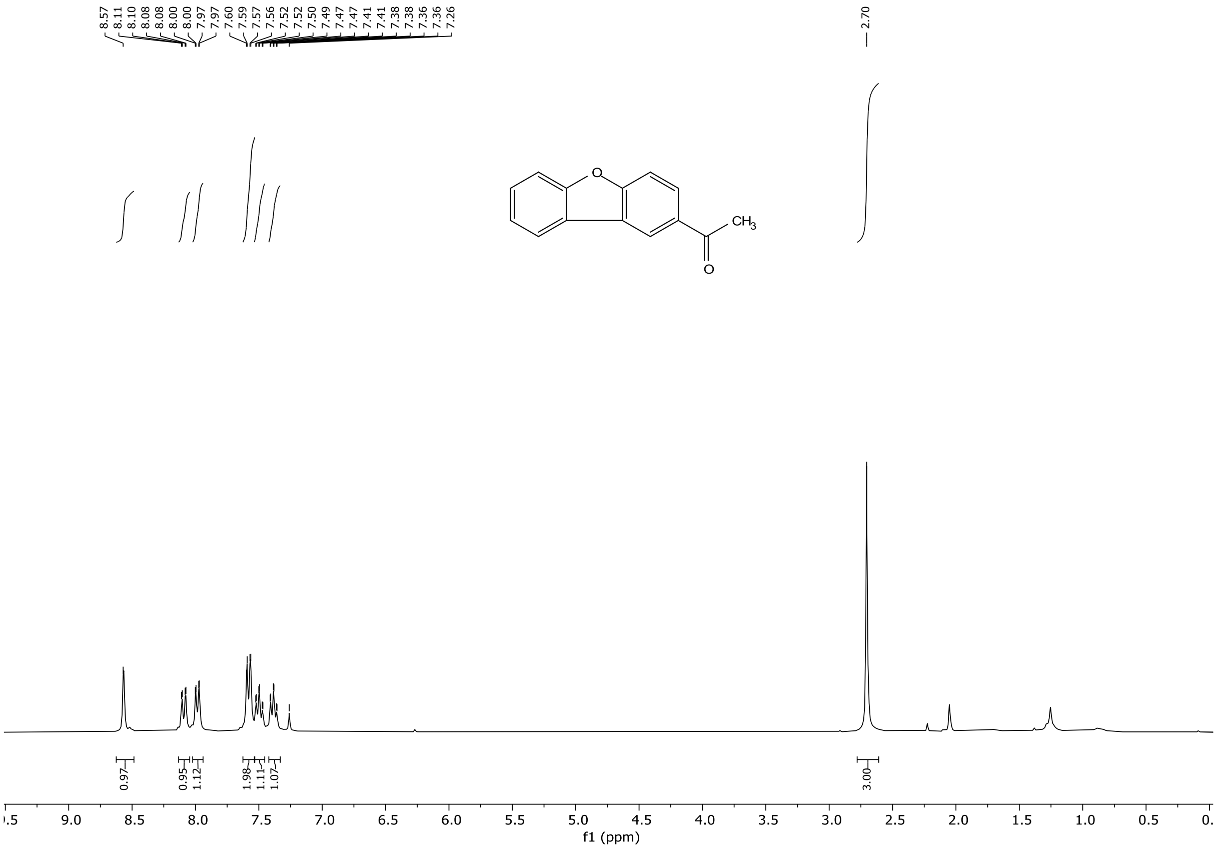
8.57 8.11 8.10 8.08 8.08 8.00 8.00 7.97 7.97 7.60 7.59 7.57 7.56 7.52 7.52 7.50 7.49 7.47 7.47 7.41 7.41 7.38 7.38 7.36 7.36 7.26
2.70
O
CH3
O
0.97
0.95
1.12
1.98
1.11
1.07
3.00
f1 (ppm)

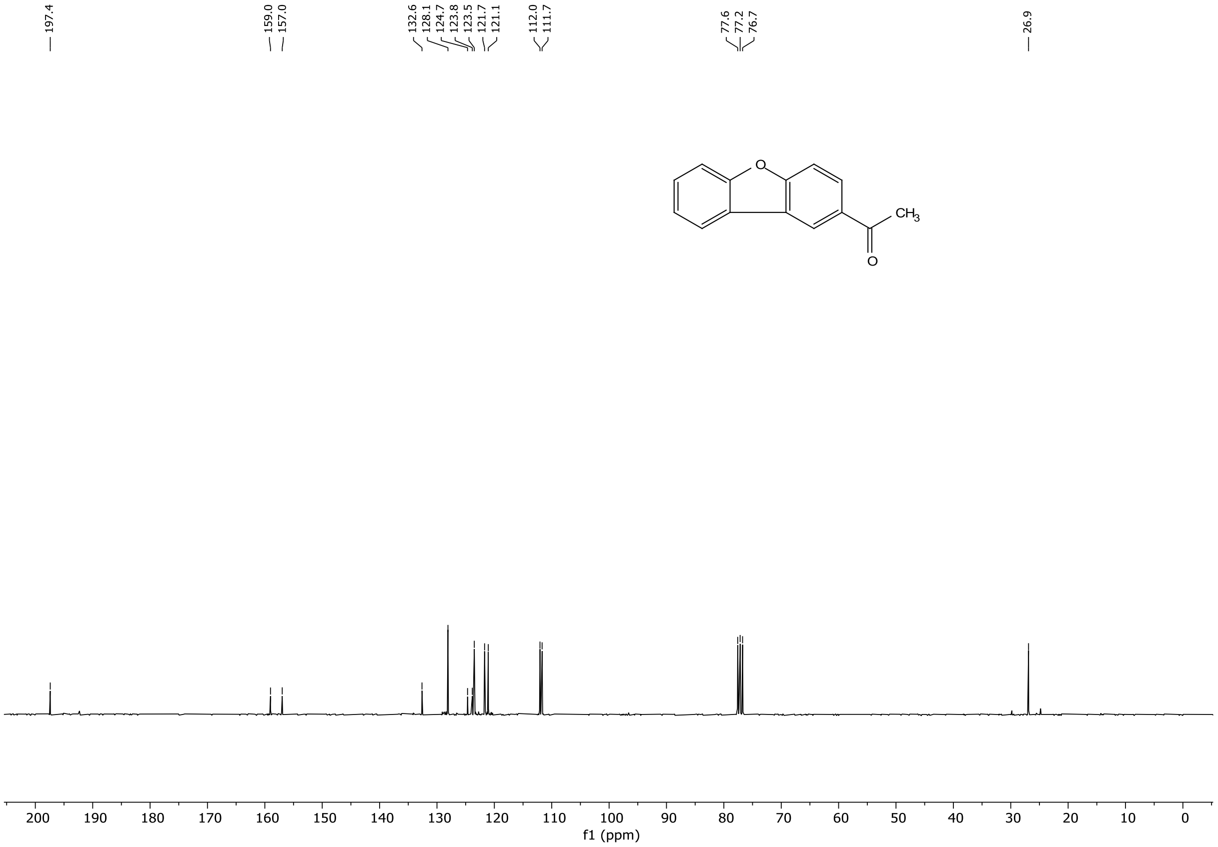
197.4
159.0 157.0
132.6 128.1 124.7 123.8 123.5 121.7 121.1
112.0 111.7
77.6 77.2 76.7
26.9
O
CH3
O
f1 (ppm)

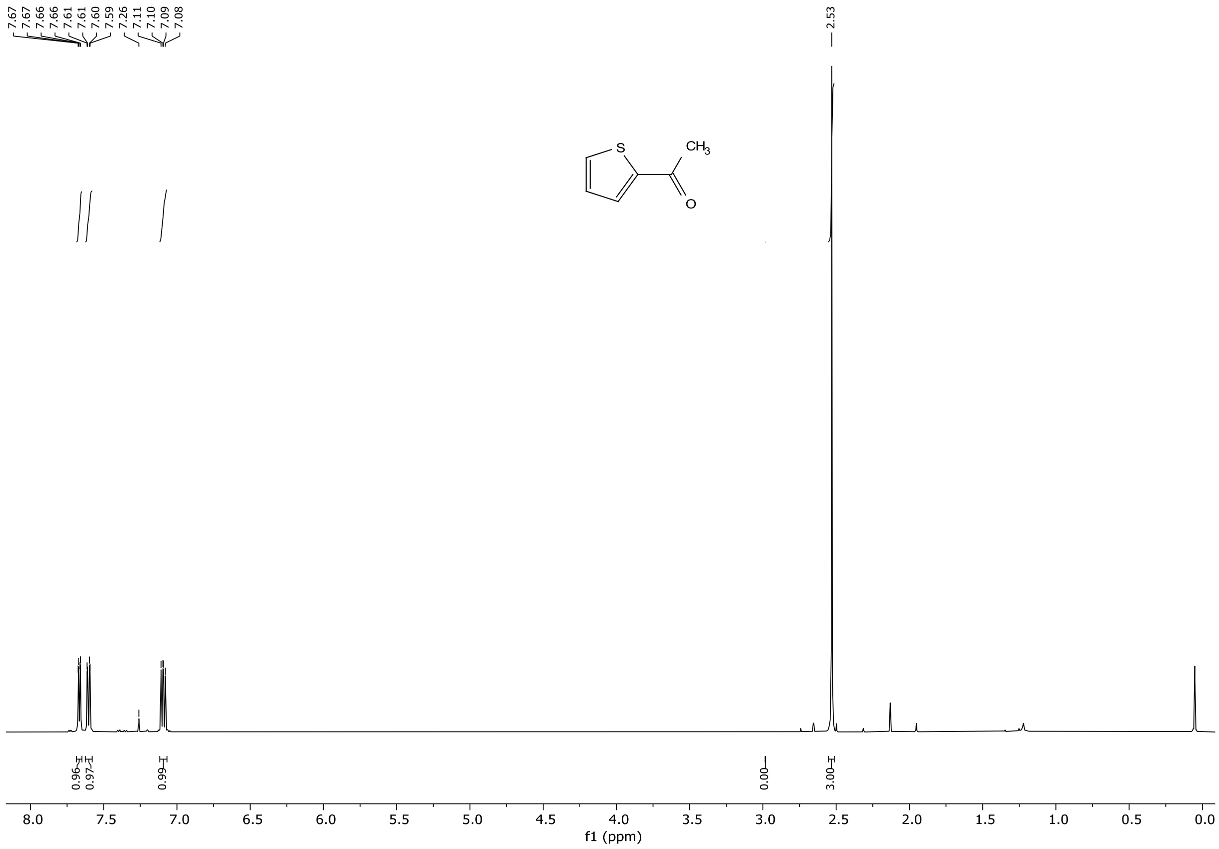
7.67
7.67
7.66
7.66
7.61
7.61
7.60
7.59
7.26
7.11
7.10
7.09
7.08
2.53
S
CH3
O
0.96
0.97
0.99
0.00
3.00
8.0 7.5 7.0 6.5 6.0 5.5 5.0 4.5 4.0 3.5 3.0 2.5 2.0 1.5 1.0 0.5 0.0
f1 (ppm)

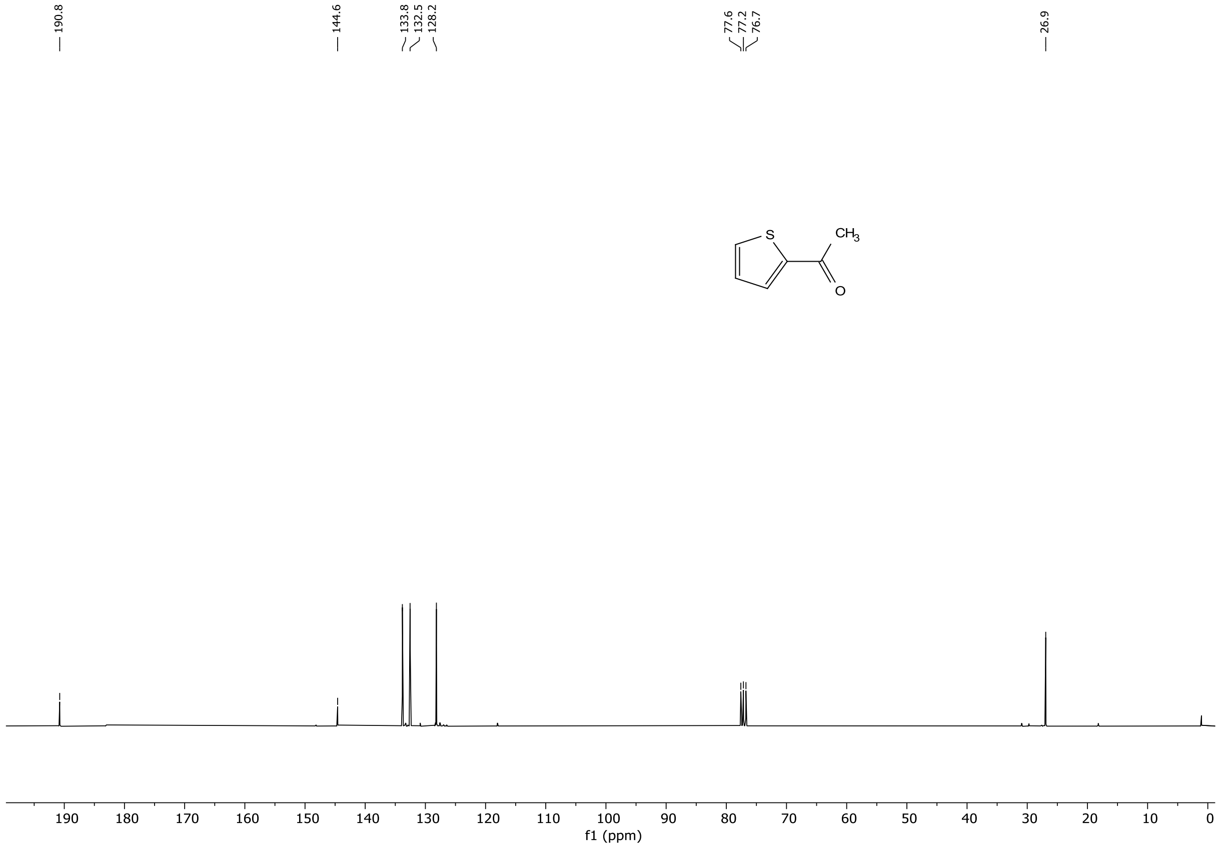
190.8
144.6
133.8
132.5
128.2
77.6
77.2
76.7
26.9
S
CH3
O
190 180 170 160 150 140 130 120 110 100 90 80 70 60 50 40 30 20 10 0
f1 (ppm)

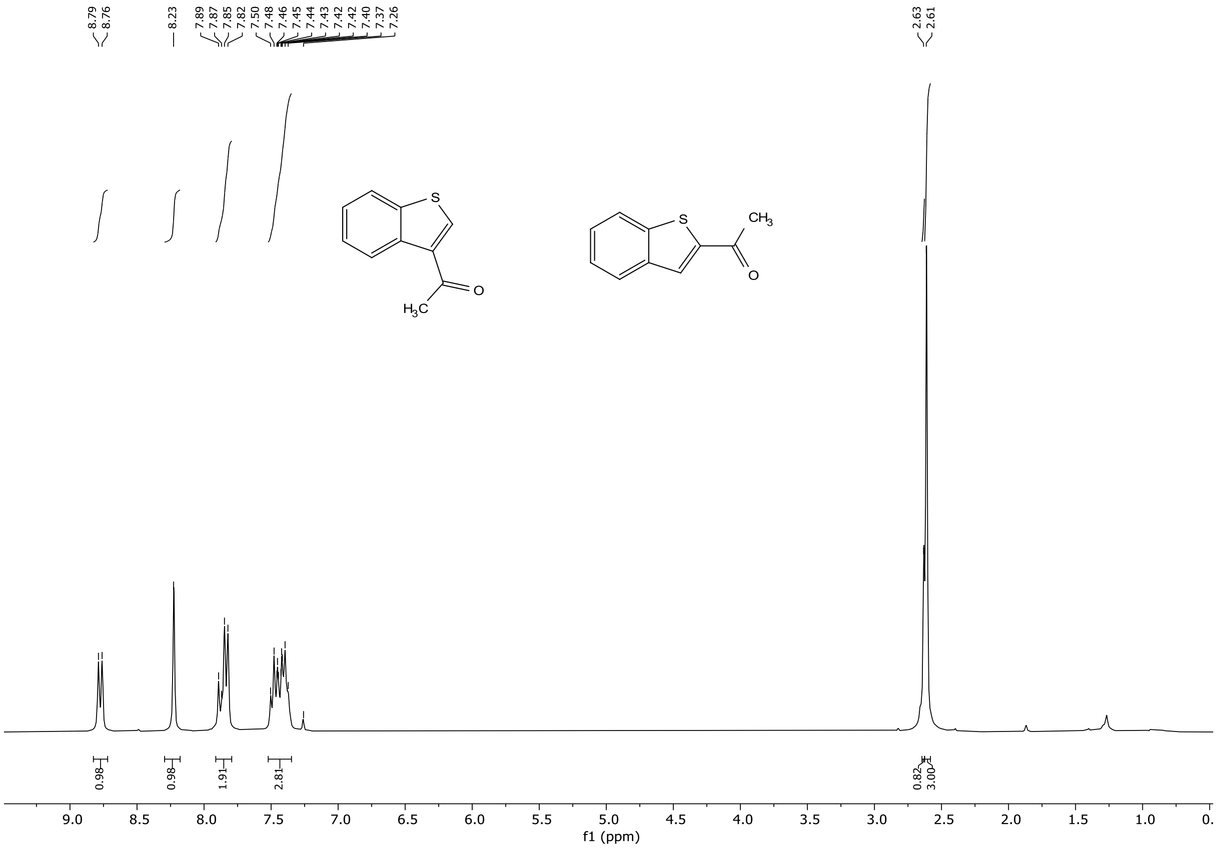
8.79
8.76
8.23
7.89
7.87
7.85
7.82
7.50
7.48
7.46
7.45
7.44
7.43
7.42
7.42
7.40
7.37
7.26
2.63
2.61
S
H3C
O
S
CH3
O
0.98
0.98
1.91
2.81
0.82
3.00
f1 (ppm)

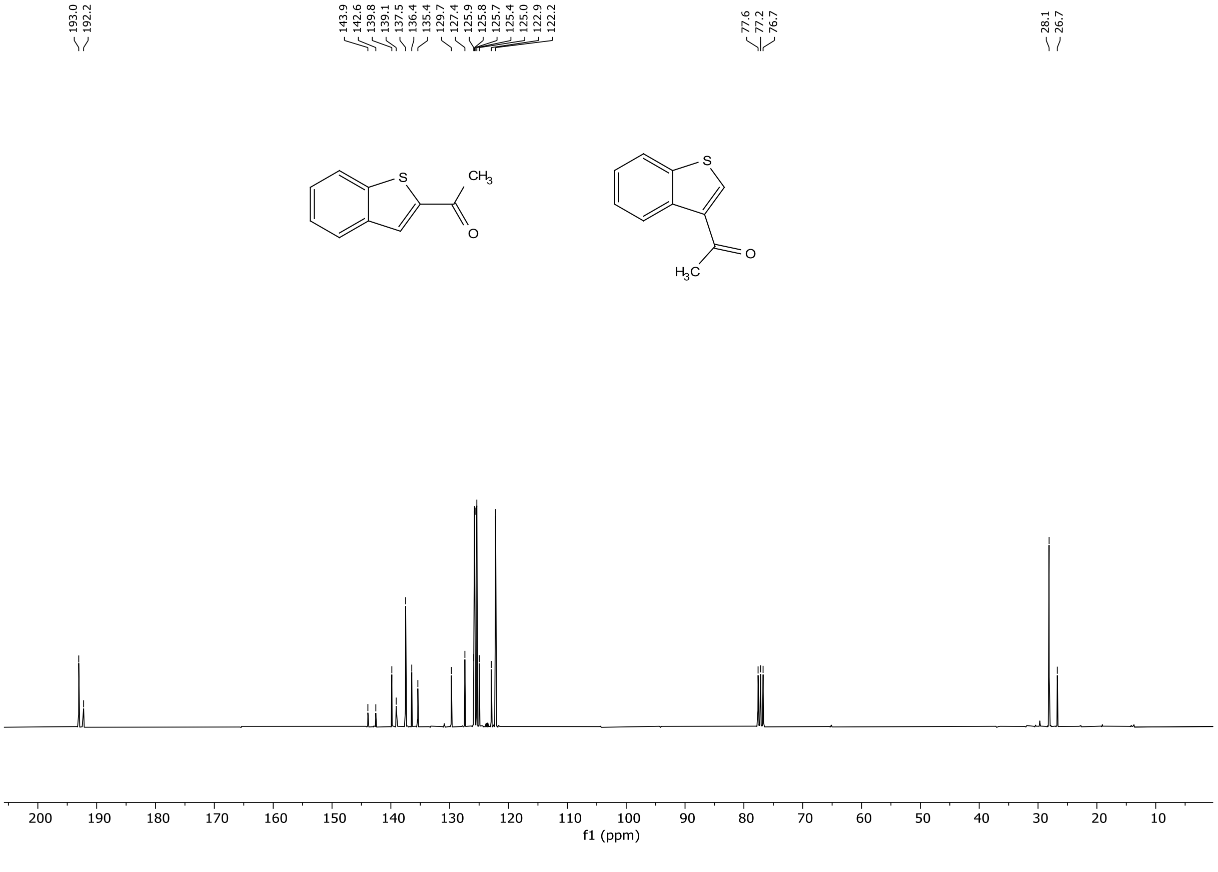
193.0
192.2
143.9
142.6
139.8
139.1
137.5
136.4
135.4
129.7
127.4
125.9
125.8
125.7
125.4
125.0
122.9
122.2
77.6
77.2
76.7
28.1
26.7
S
CH3
O
S
H3C
O
f1 (ppm)

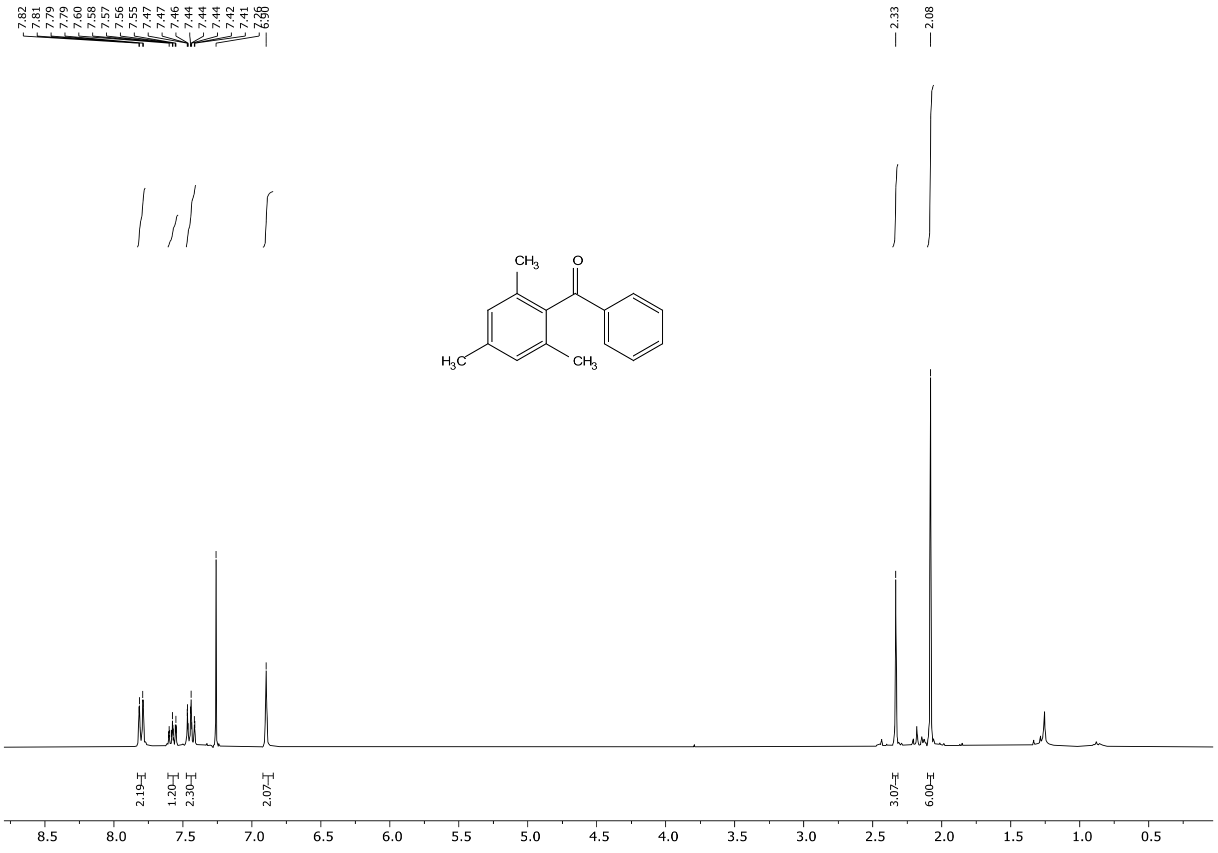
7.82 7.81 7.79 7.79 7.60 7.58 7.57 7.56 7.55 7.47 7.47 7.46 7.44 7.44 7.44 7.42 7.41 7.26 6.90
2.33
2.08
CH_3 O
H_3C CH_3
2.19 1.20 2.30 2.07 3.07 6.00
8.5 8.0 7.5 7.0 6.5 6.0 5.5 5.0 4.5 4.0 3.5 3.0 2.5 2.0 1.5 1.0 0.5

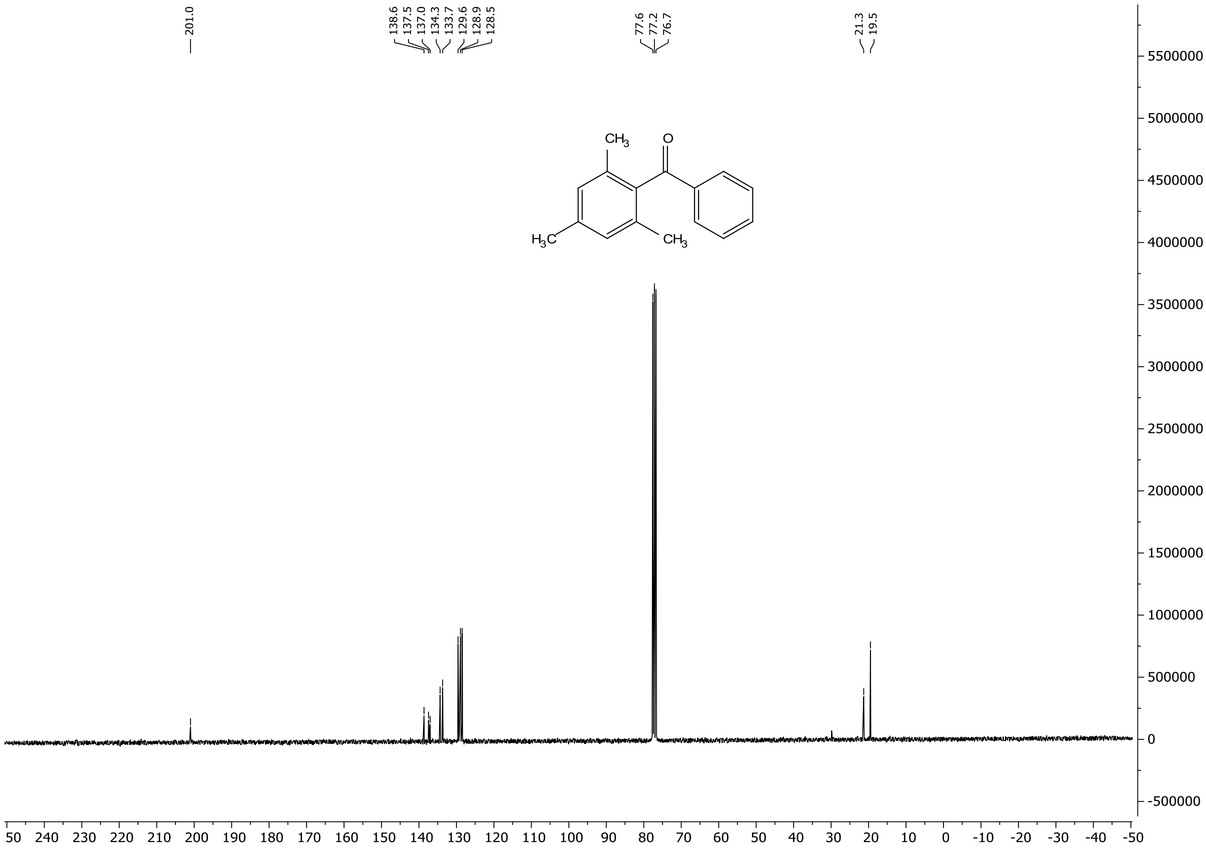
201.0
138.6 137.5 137.0 134.3 133.7 129.6 128.9 128.5
77.6 77.2 76.7
21.3 19.5
CH_3 O
H_3C CH_3
5500000 5000000 4500000 4000000 3500000 3000000 2500000 2000000 1500000 1000000 500000 0 -500000
50 240 230 220 210 200 190 180 170 160 150 140 130 120 110 100 90 80 70 60 50 40 30 20 10 0 -10 -20 -30 -40 -50

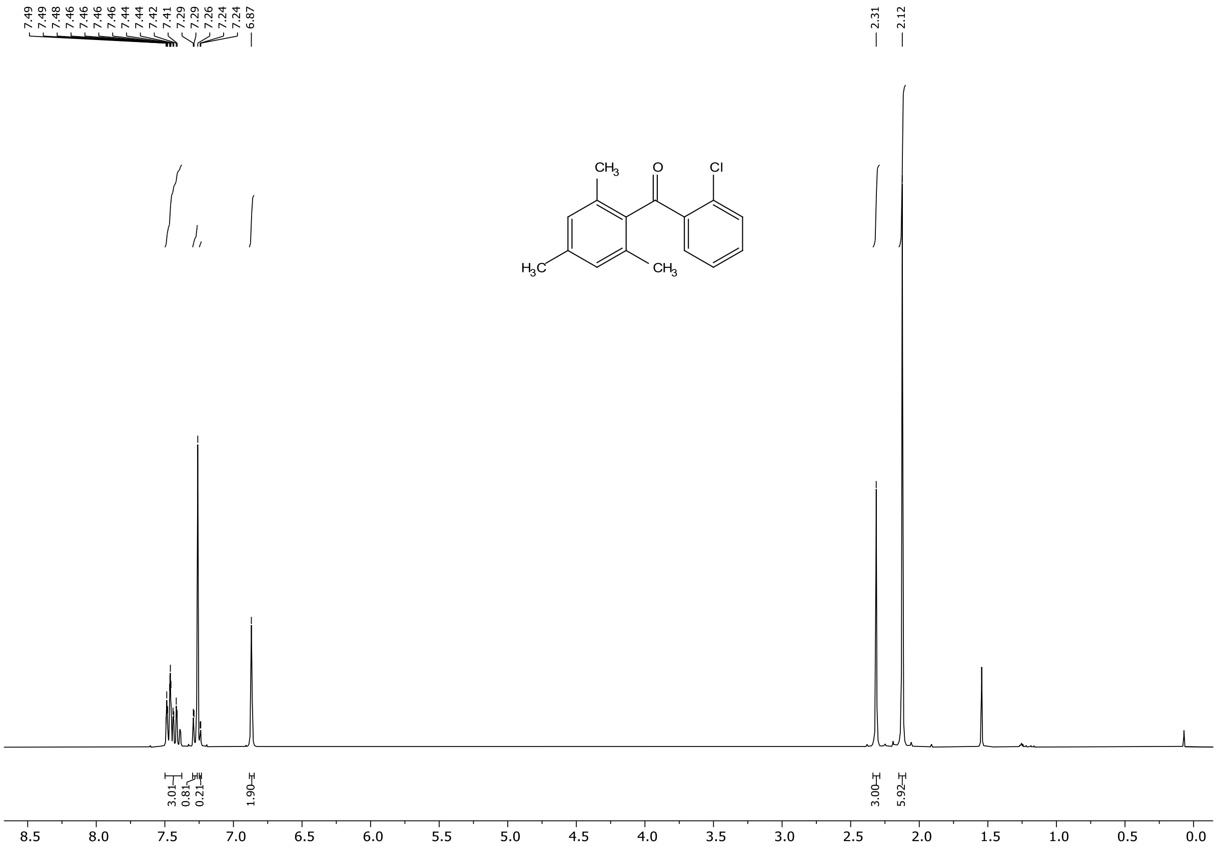
7.49
7.49
7.48
7.46
7.46
7.46
7.44
7.44
7.42
7.41
7.29
7.29
7.26
7.24
7.24
6.87
2.31
2.12
CH3
O
Cl
H3C
CH3
3.01
0.81
0.21
1.90
3.00
5.92
8.5 8.0 7.5 7.0 6.5 6.0 5.5 5.0 4.5 4.0 3.5 3.0 2.5 2.0 1.5 1.0 0.5 0.0

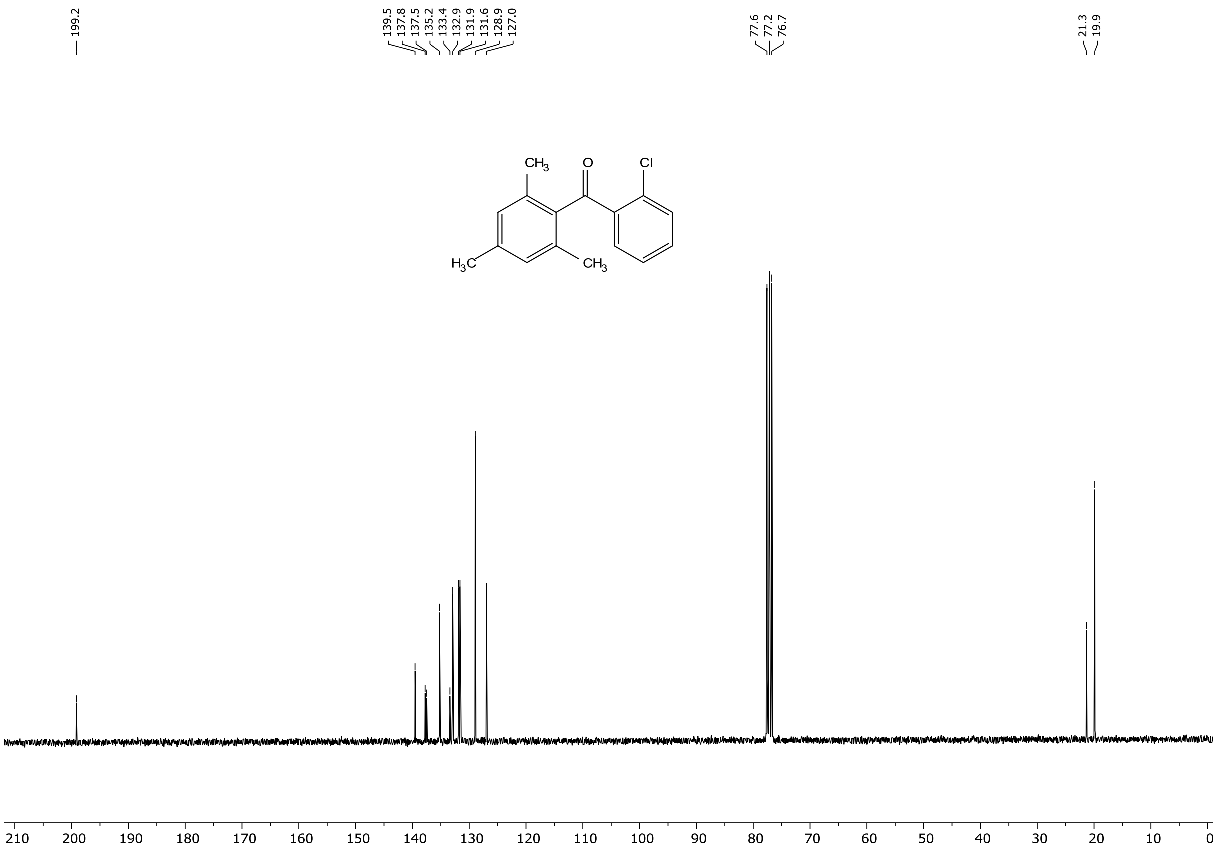
199.2
139.5
137.8
137.5
135.2
133.4
132.9
131.9
131.6
128.9
127.0
77.6
77.2
76.7
21.3
19.9
CH3
O
Cl
H3C
CH3
210 200 190 180 170 160 150 140 130 120 110 100 90 80 70 60 50 40 30 20 10 0

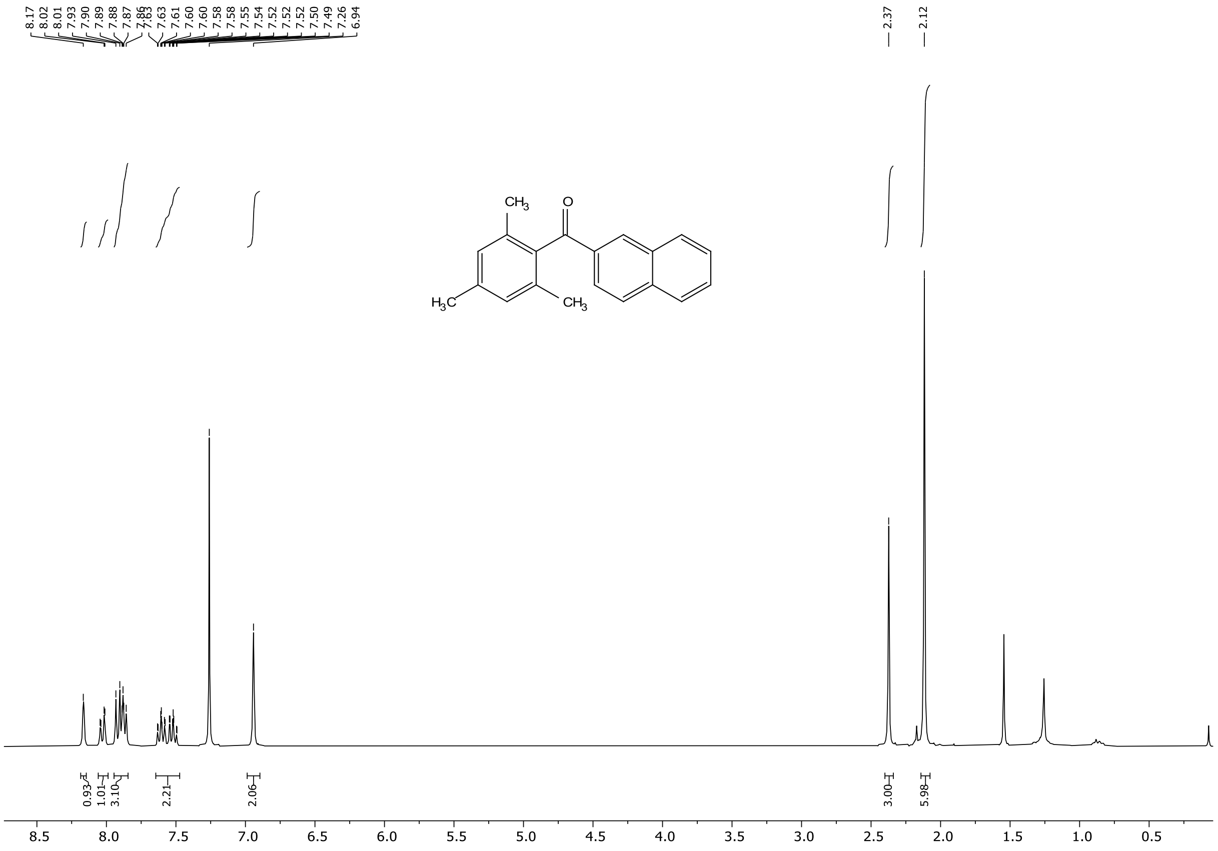
8.17
8.02
8.01
7.93
7.90
7.89
7.88
7.87
7.86
7.63
7.63
7.61
7.60
7.60
7.58
7.58
7.55
7.54
7.52
7.52
7.52
7.52
7.50
7.49
7.26
6.94
2.37
2.12
CH_3
O
H_3C
CH_3
0.93
1.01
3.10
2.21
2.06
3.00
5.98
8.5
8.0
7.5
7.0
6.5
6.0
5.5
5.0
4.5
4.0
3.5
3.0
2.5
2.0
1.5
1.0
0.5

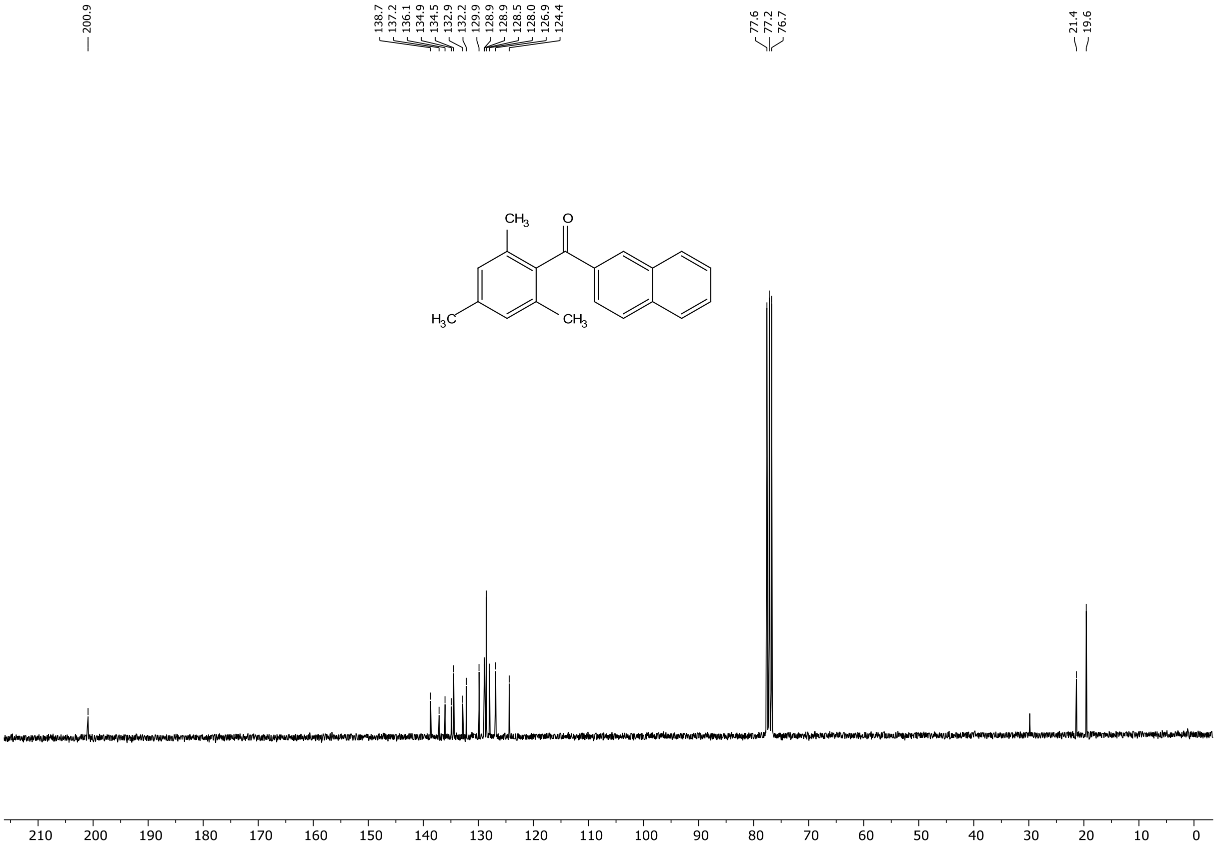
200.9
138.7
137.2
136.1
134.9
134.5
132.9
132.2
129.9
128.9
128.9
128.5
128.0
126.9
124.4
77.6
77.2
76.7
21.4
19.6
CH_3
O
H_3C
CH_3
210
200
190
180
170
160
150
140
130
120
110
100
90
80
70
60
50
40
30
20
10
0

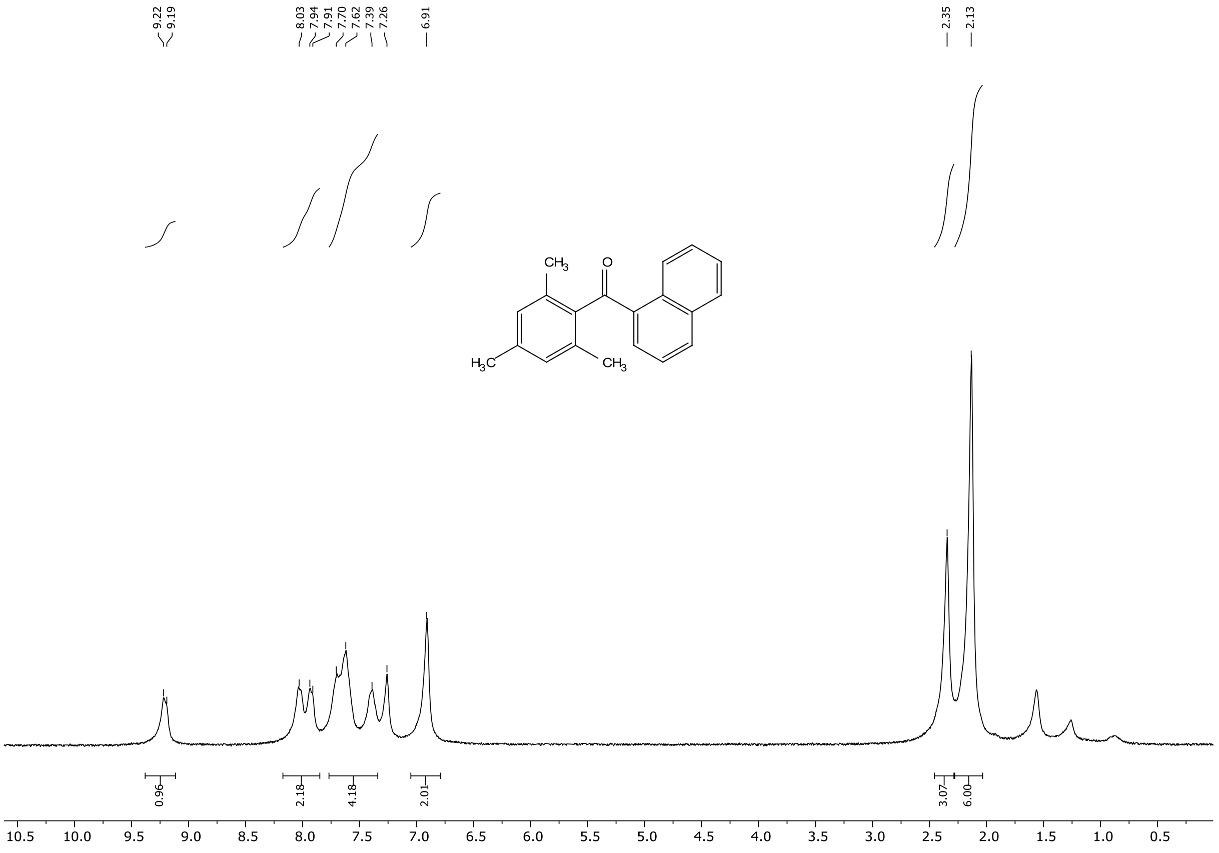
9.22
9.19
8.03
7.94
7.91
7.70
7.62
7.39
7.26
6.91
2.35
2.13
CH3
O
H3C
CH3
0.96
2.18
4.18
2.01
3.07
6.00
10.5 10.0 9.5 9.0 8.5 8.0 7.5 7.0 6.5 6.0 5.5 5.0 4.5 4.0 3.5 3.0 2.5 2.0 1.5 1.0 0.5

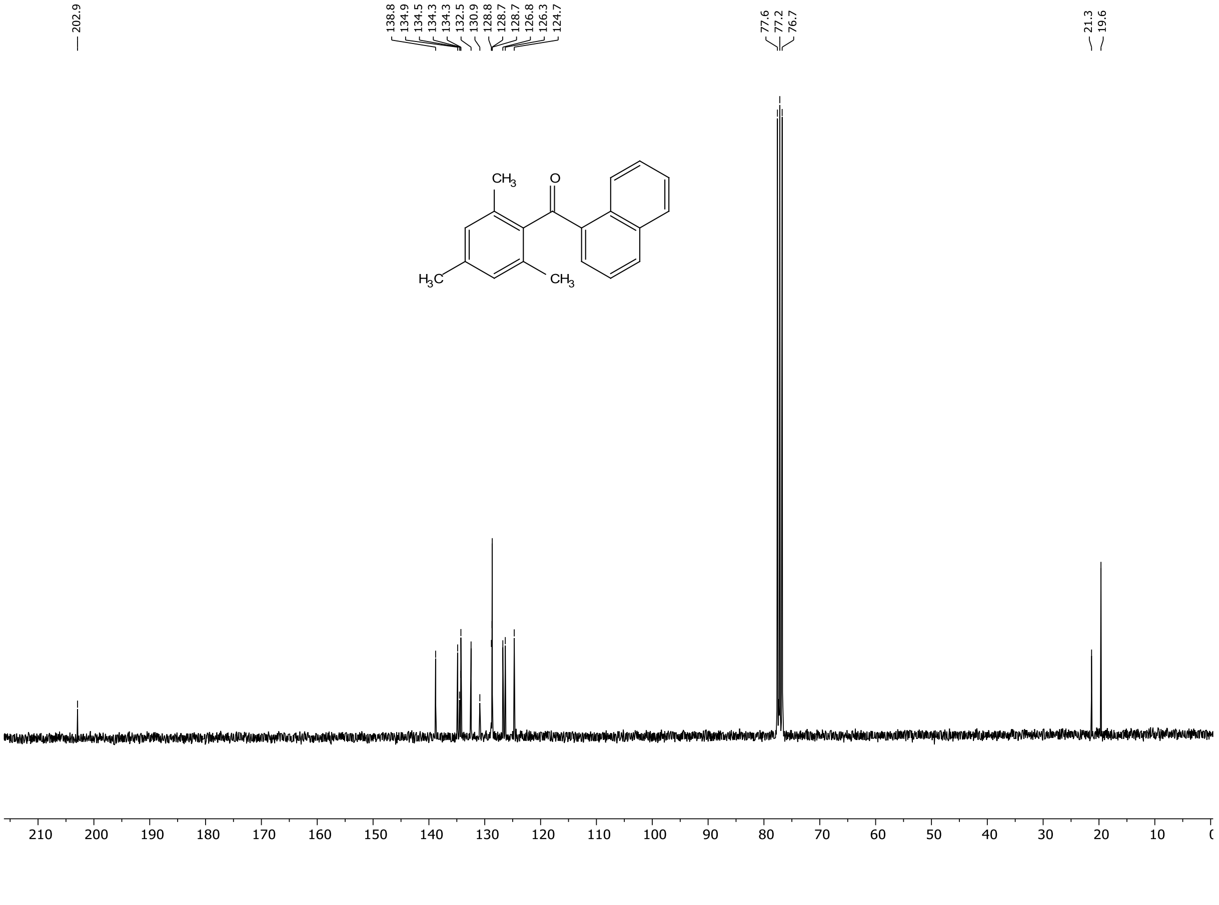
202.9
138.8
134.9
134.5
134.3
134.3
132.5
130.9
128.8
128.7
128.7
126.8
126.3
124.7
77.6
77.2
76.7
21.3
19.6
CH3
O
H3C
CH3
210 200 190 180 170 160 150 140 130 120 110 100 90 80 70 60 50 40 30 20 10

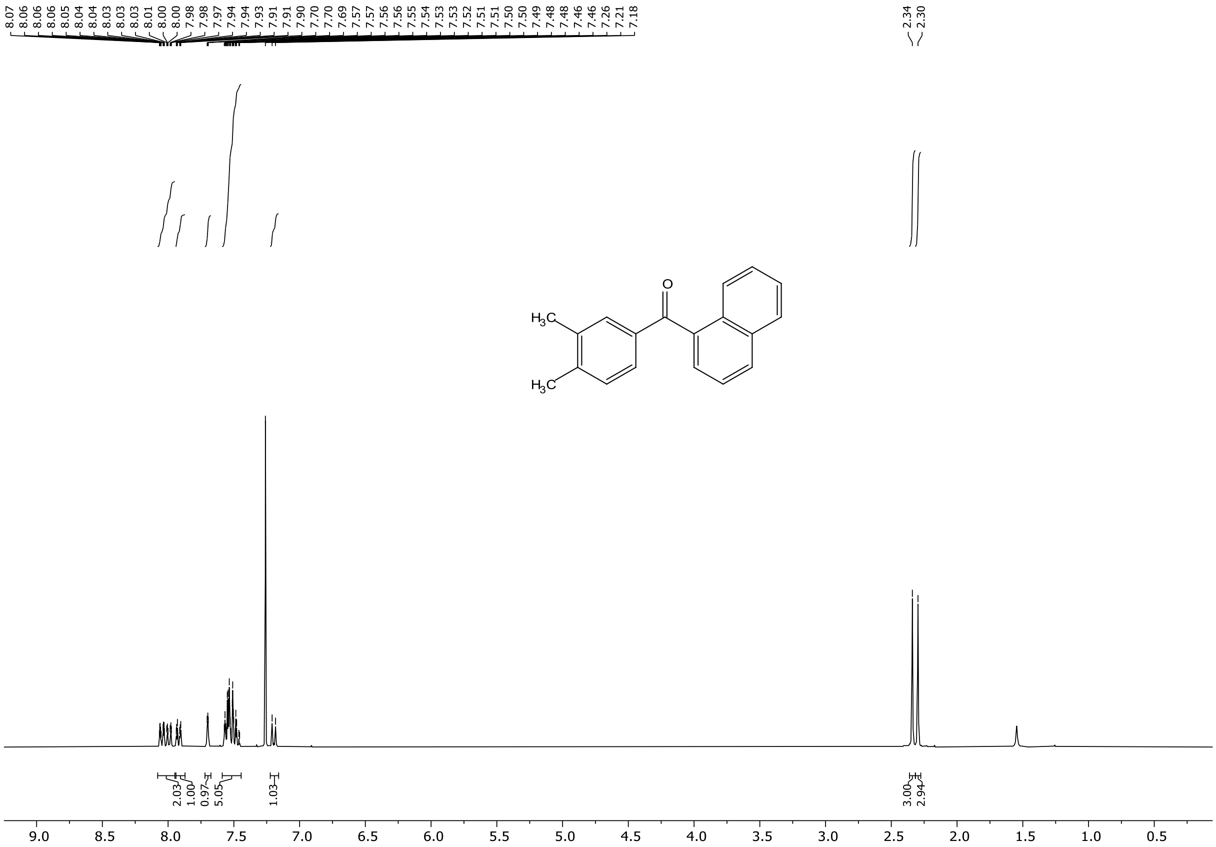
8.07 8.06 8.06 8.05 8.04 8.04 8.03 8.03 8.03 8.01 8.00 8.00 7.98 7.98 7.97 7.94 7.94 7.93 7.91 7.91 7.90 7.70 7.70 7.69 7.57 7.57 7.56 7.56 7.55 7.54 7.53 7.53 7.52 7.51 7.51 7.50 7.49 7.48 7.48 7.46 7.46 7.26 7.21 7.18
2.34 2.30
O
H_3C
H_3C
2.03 1.00 0.97 5.05 1.03
3.00 2.94
9.0 8.5 8.0 7.5 7.0 6.5 6.0 5.5 5.0 4.5 4.0 3.5 3.0 2.5 2.0 1.5 1.0 0.5

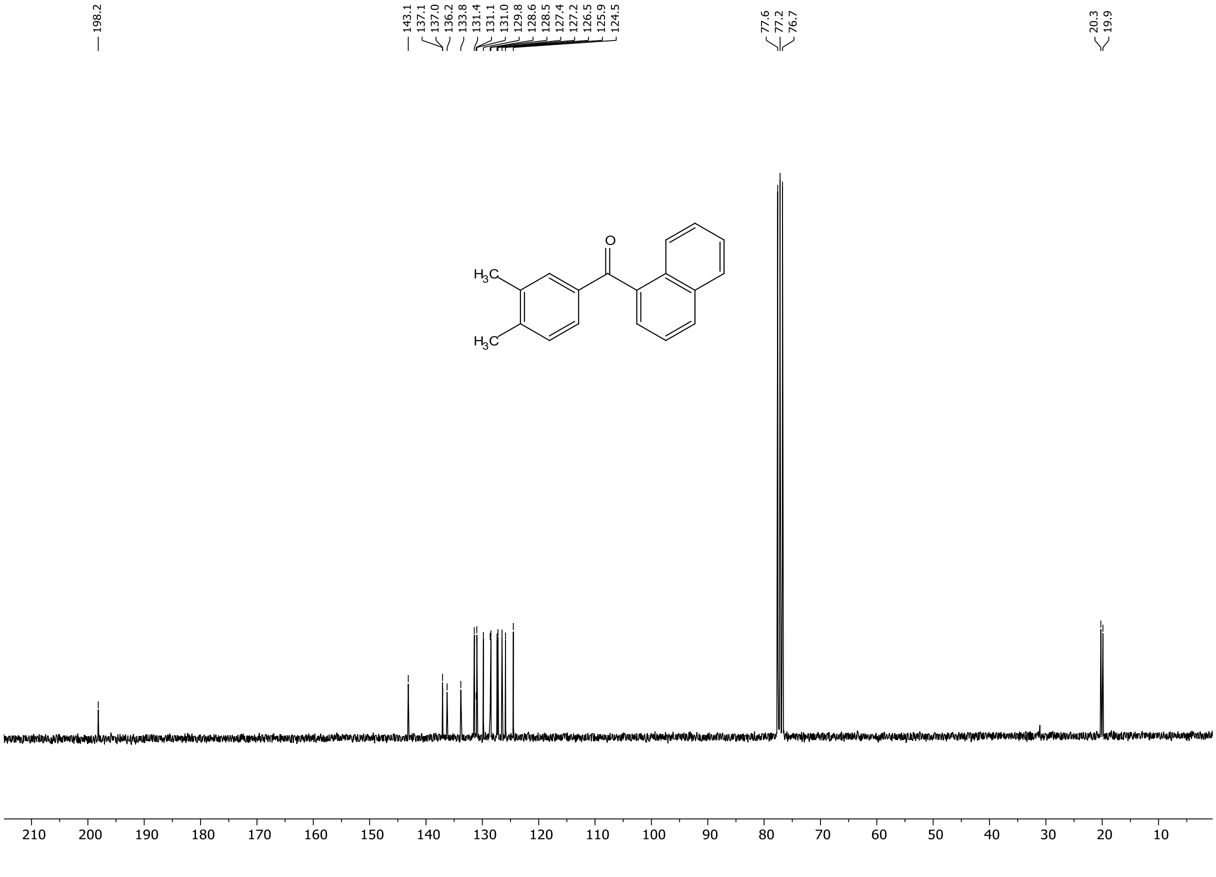
198.2
143.1 137.1 137.0 136.2 133.8 131.4 131.1 131.0 129.8 128.6 128.5 127.4 127.2 126.5 125.9 124.5
77.6 77.2 76.7
20.3 19.9
O
H_3C
H_3C
210 200 190 180 170 160 150 140 130 120 110 100 90 80 70 60 50 40 30 20 10

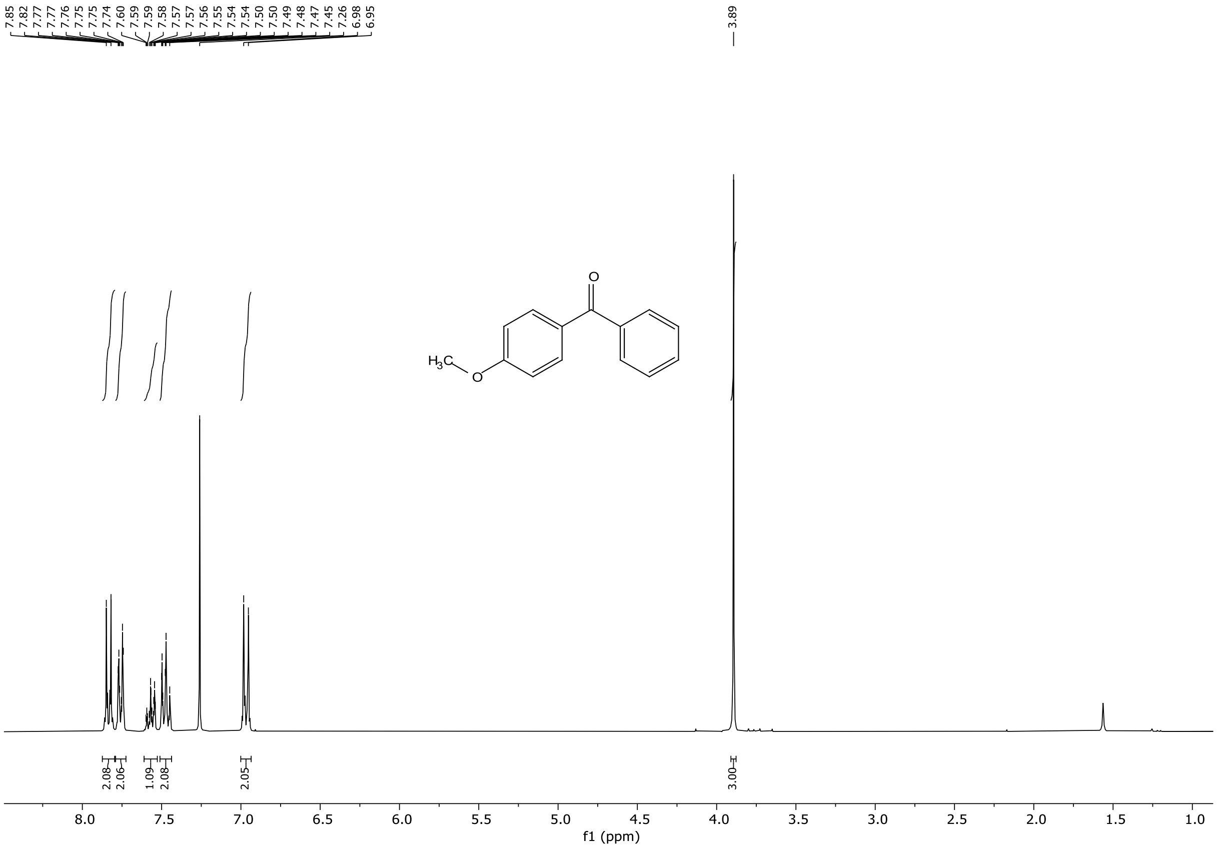
7.85
7.82
7.77
7.77
7.76
7.75
7.75
7.74
7.60
7.59
7.59
7.58
7.57
7.57
7.56
7.55
7.54
7.54
7.50
7.50
7.49
7.48
7.47
7.45
7.26
6.98
6.95
3.89
H3C
O
O
2.08
2.06
1.09
2.08
2.05
3.00
8.0
7.5
7.0
6.5
6.0
5.5
5.0
4.5
4.0
3.5
3.0
2.5
2.0
1.5
1.0
f1 (ppm)

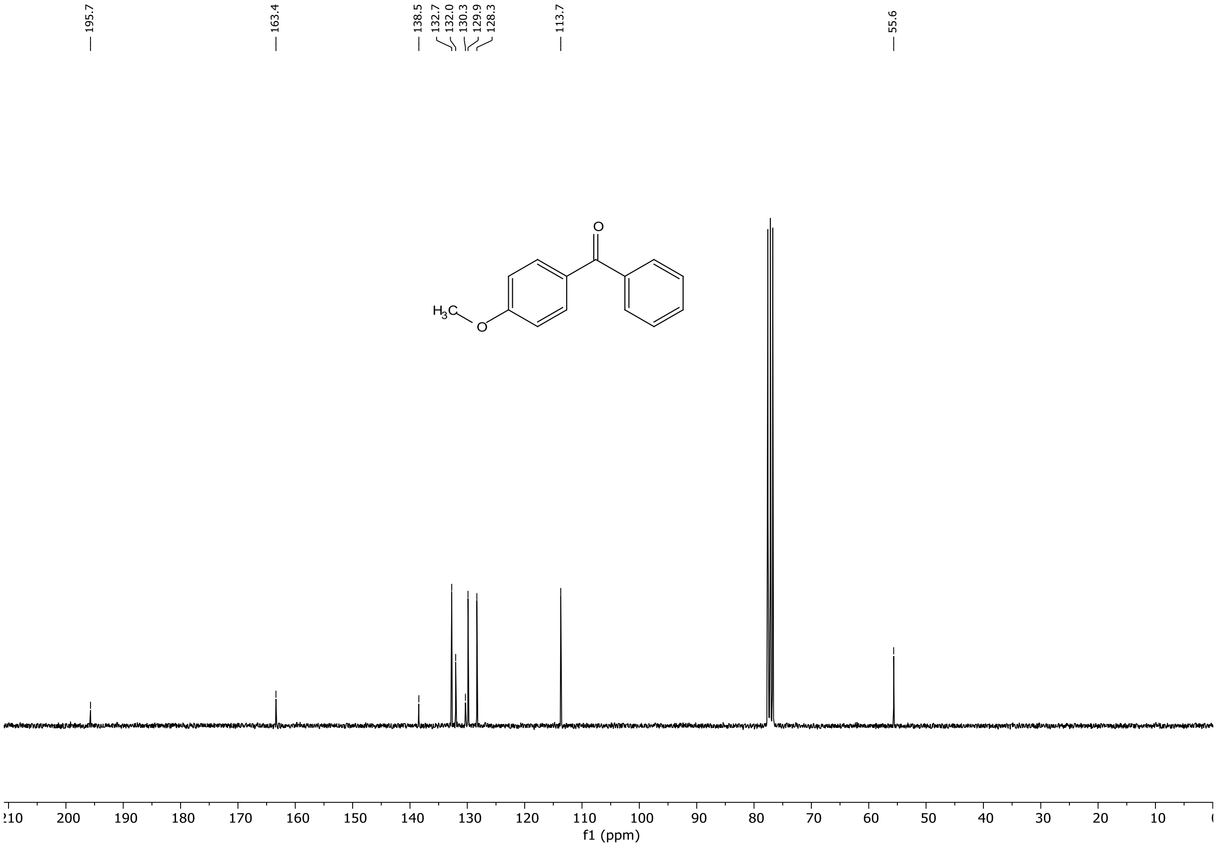
195.7
163.4
138.5
132.7
132.0
130.3
129.9
128.3
113.7
55.6
H3C
O
O
210
200
190
180
170
160
150
140
130
120
110
100
90
80
70
60
50
40
30
20
10
f1 (ppm)

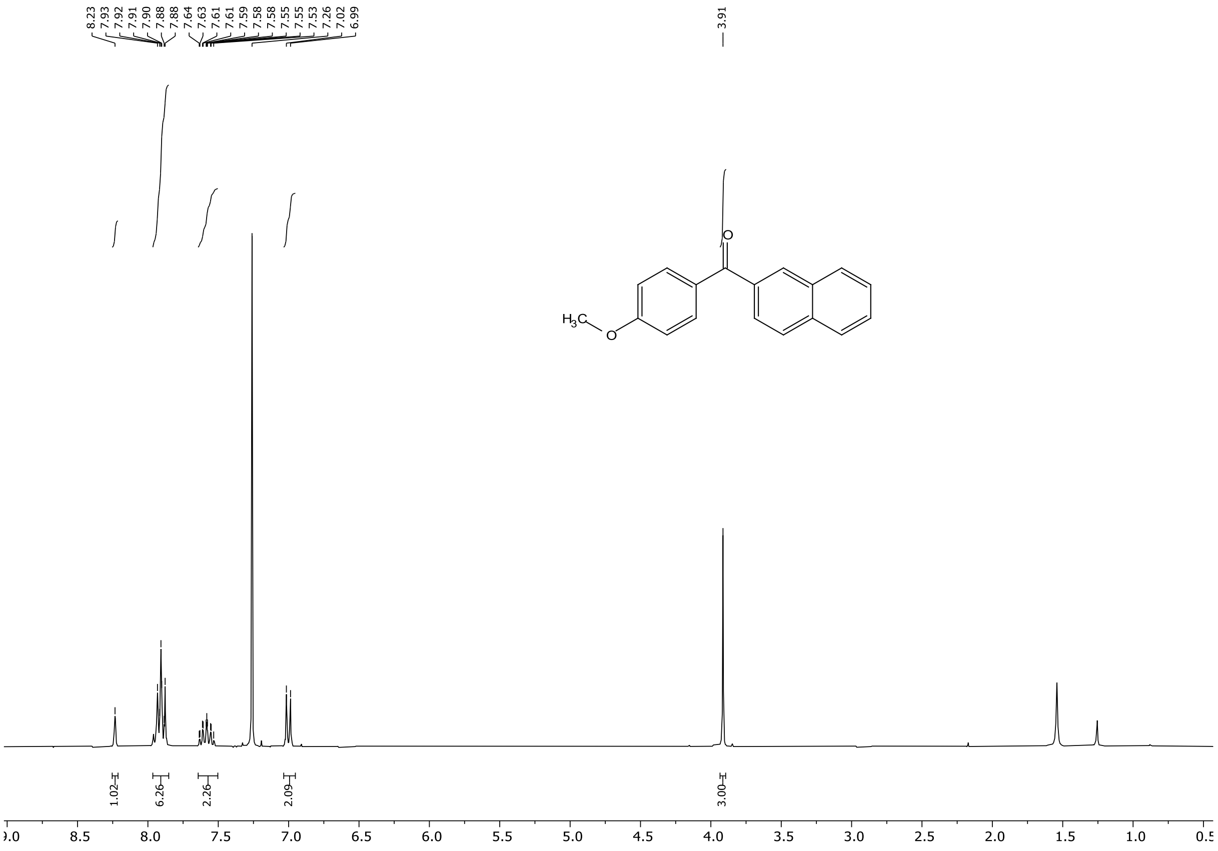
8.23
7.93
7.92
7.91
7.90
7.88
7.88
7.64
7.63
7.61
7.61
7.59
7.58
7.58
7.55
7.55
7.55
7.53
7.26
7.02
6.99
3.91
O
H3C
O
1.02
6.26
2.26
2.09
3.00
8.5
8.0
7.5
7.0
6.5
6.0
5.5
5.0
4.5
4.0
3.5
3.0
2.5
2.0
1.5
1.0

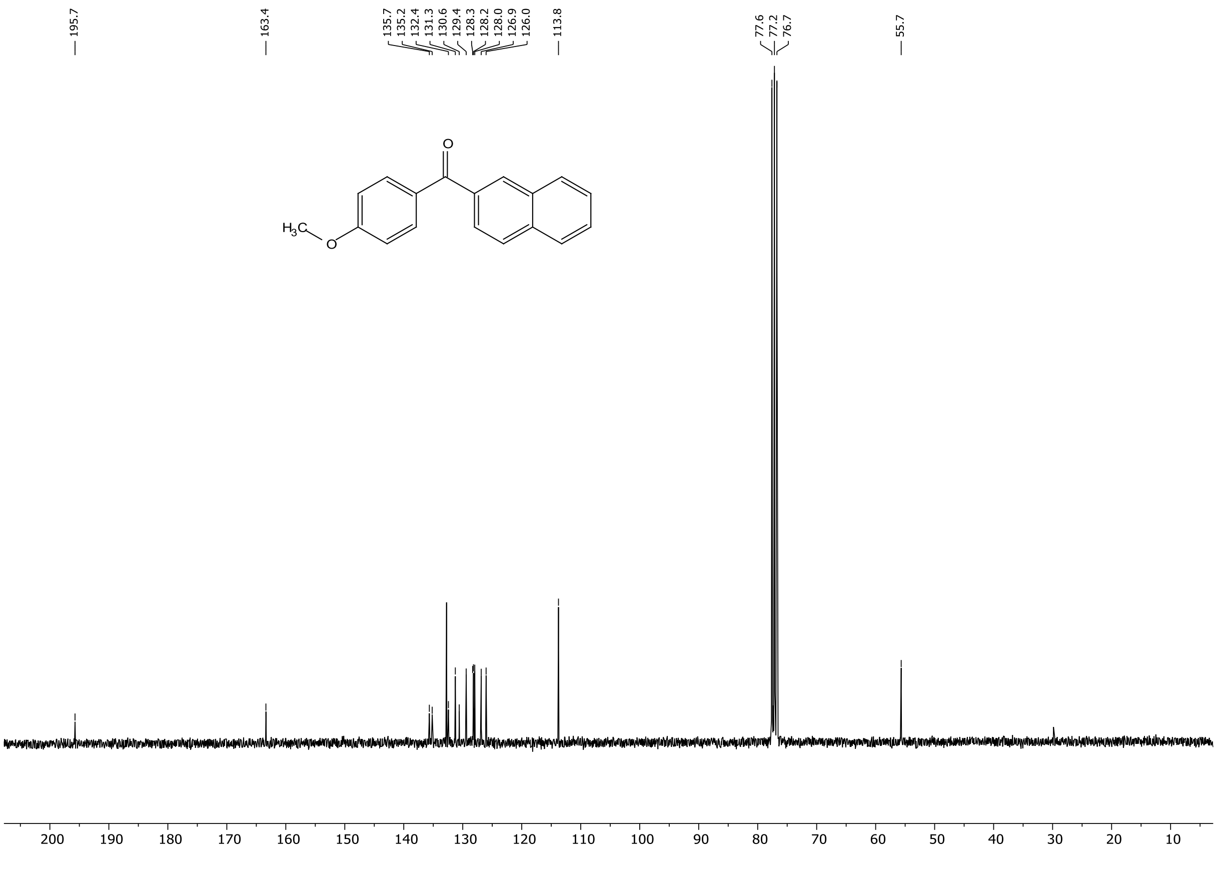
195.7
163.4
135.7
135.2
132.4
131.3
130.6
129.4
128.3
128.2
128.0
126.9
126.0
113.8
77.6
77.2
76.7
55.7
O
H3C
O
200
190
180
170
160
150
140
130
120
110
100
90
80
70
60
50
40
30
20
10

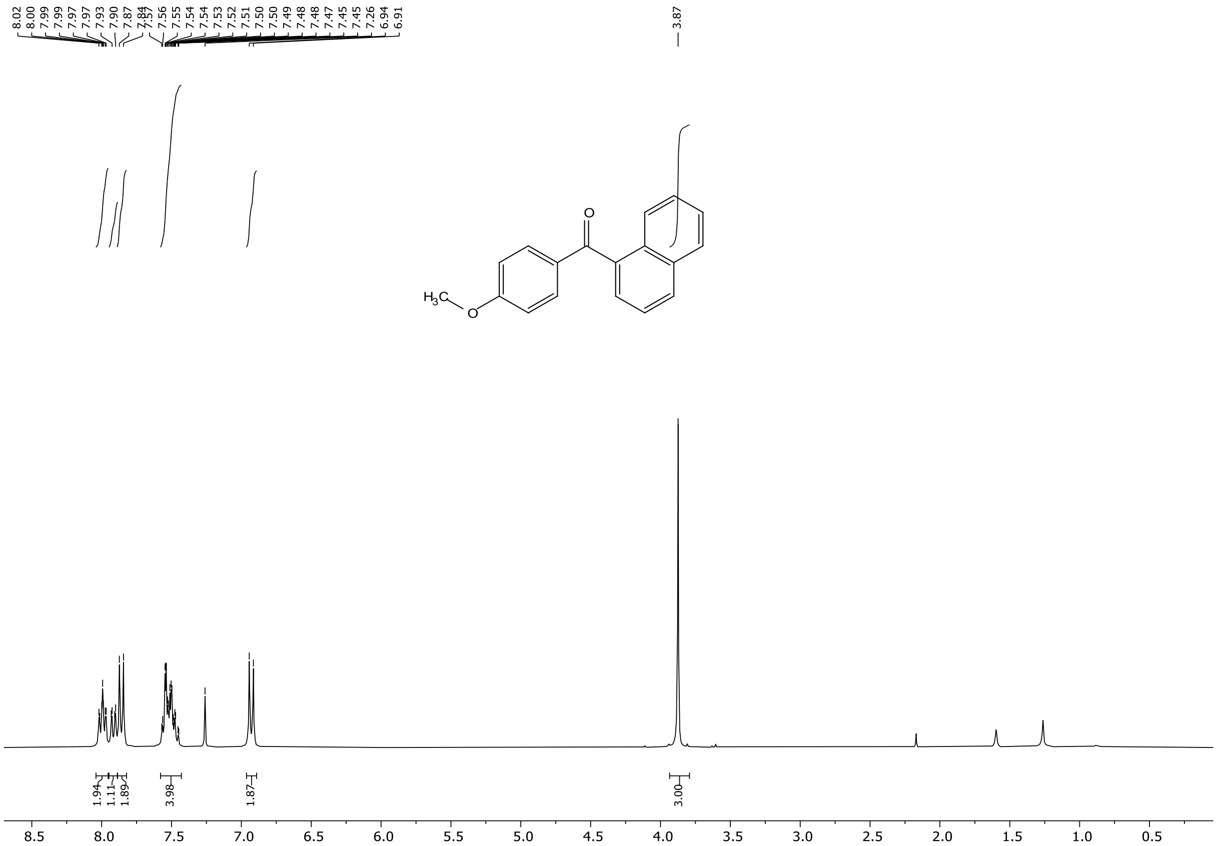
8.02
8.00
7.99
7.99
7.97
7.97
7.93
7.90
7.87
7.84
7.57
7.56
7.55
7.54
7.54
7.53
7.52
7.51
7.50
7.50
7.49
7.48
7.48
7.47
7.45
7.45
7.26
6.94
6.91
3.87
O
H3C
O
1.94
1.11
1.89
3.98
1.87
3.00
8.5
8.0
7.5
7.0
6.5
6.0
5.5
5.0
4.5
4.0
3.5
3.0
2.5
2.0
1.5
1.0
0.5

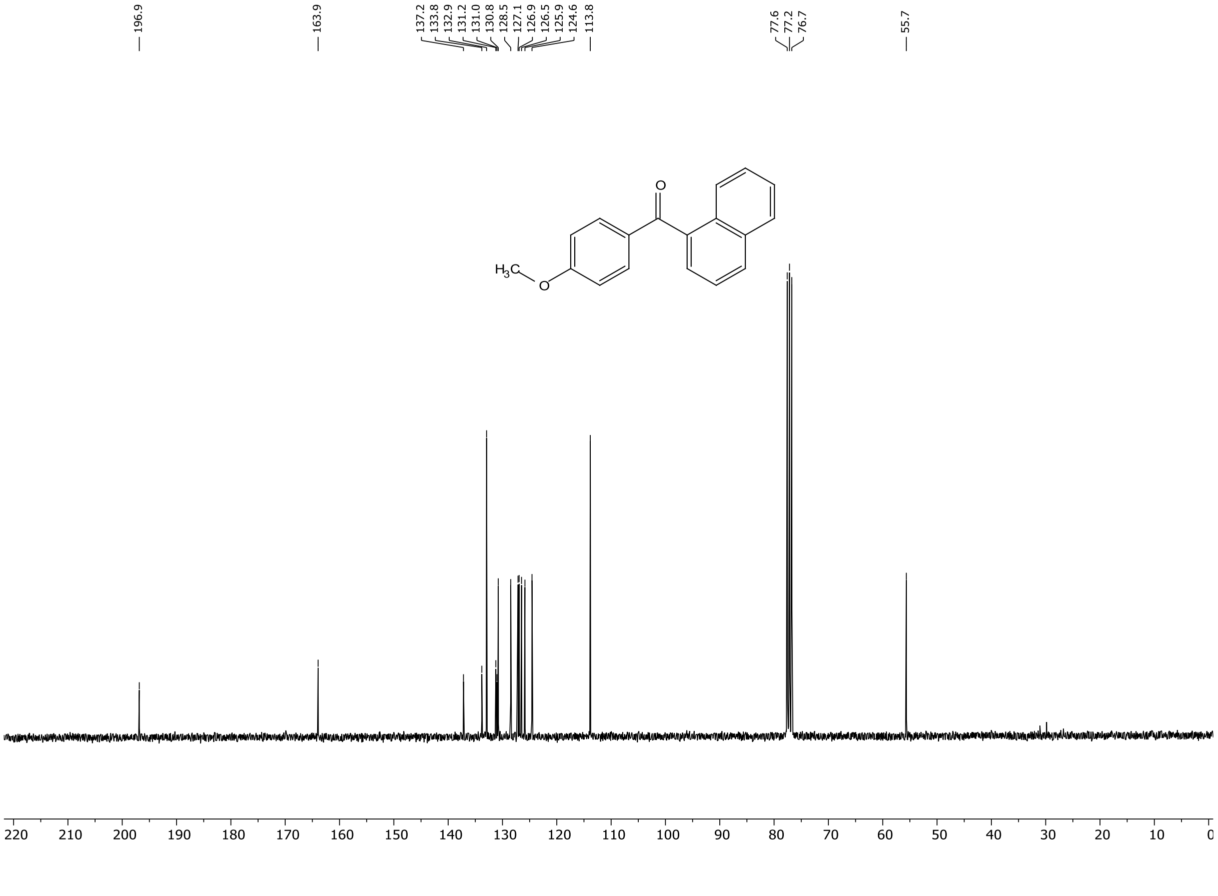
196.9
163.9
137.2
133.8
132.9
131.2
131.0
130.8
128.5
127.1
126.9
126.5
125.9
124.6
113.8
77.6
77.2
76.7
55.7
O
H3C
O
220
210
200
190
180
170
160
150
140
130
120
110
100
90
80
70
60
50
40
30
20
10
0

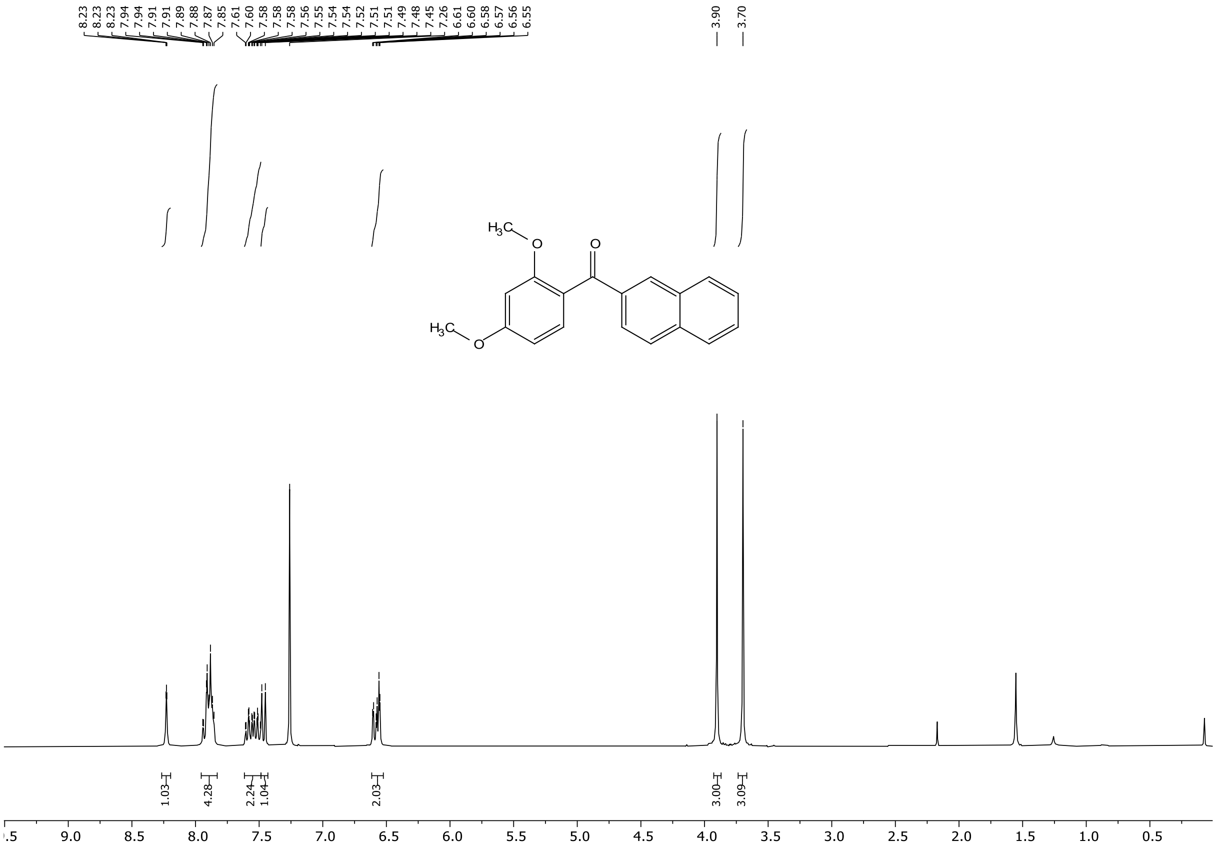

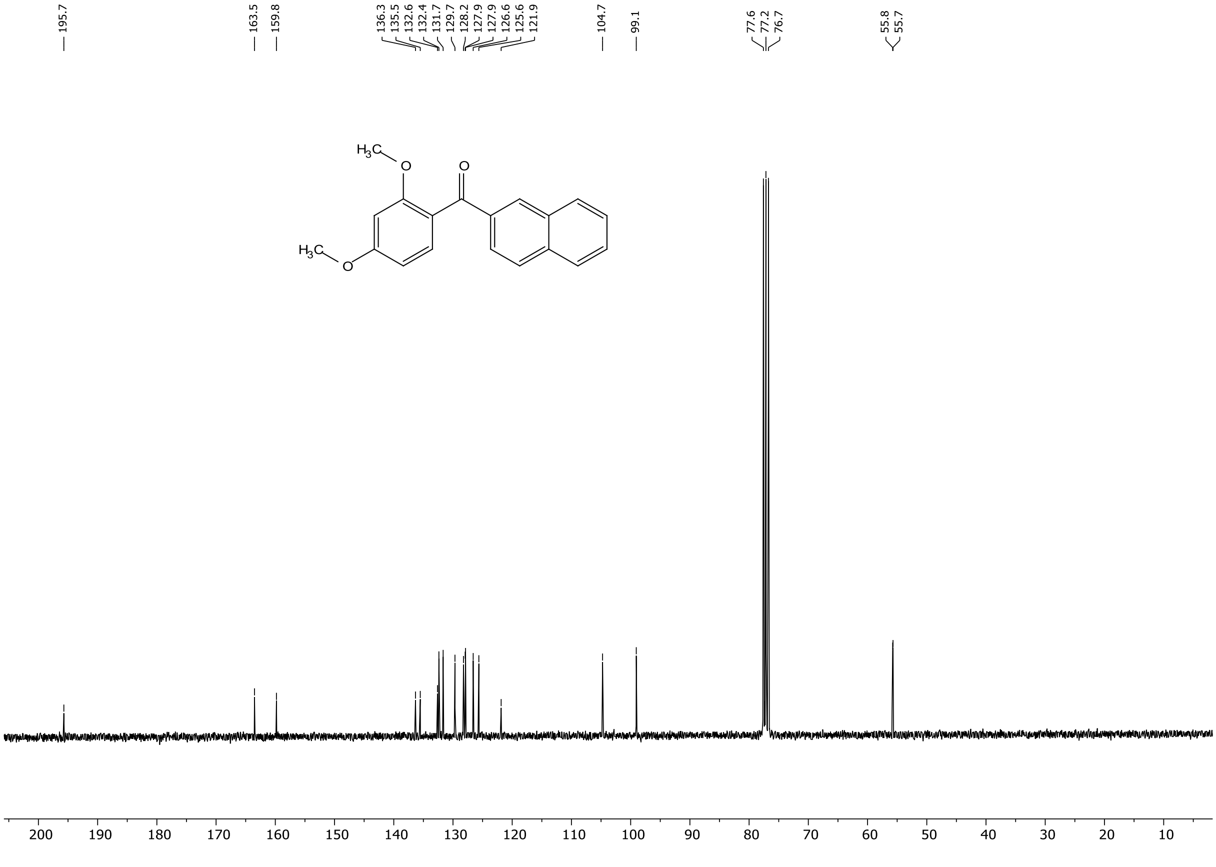

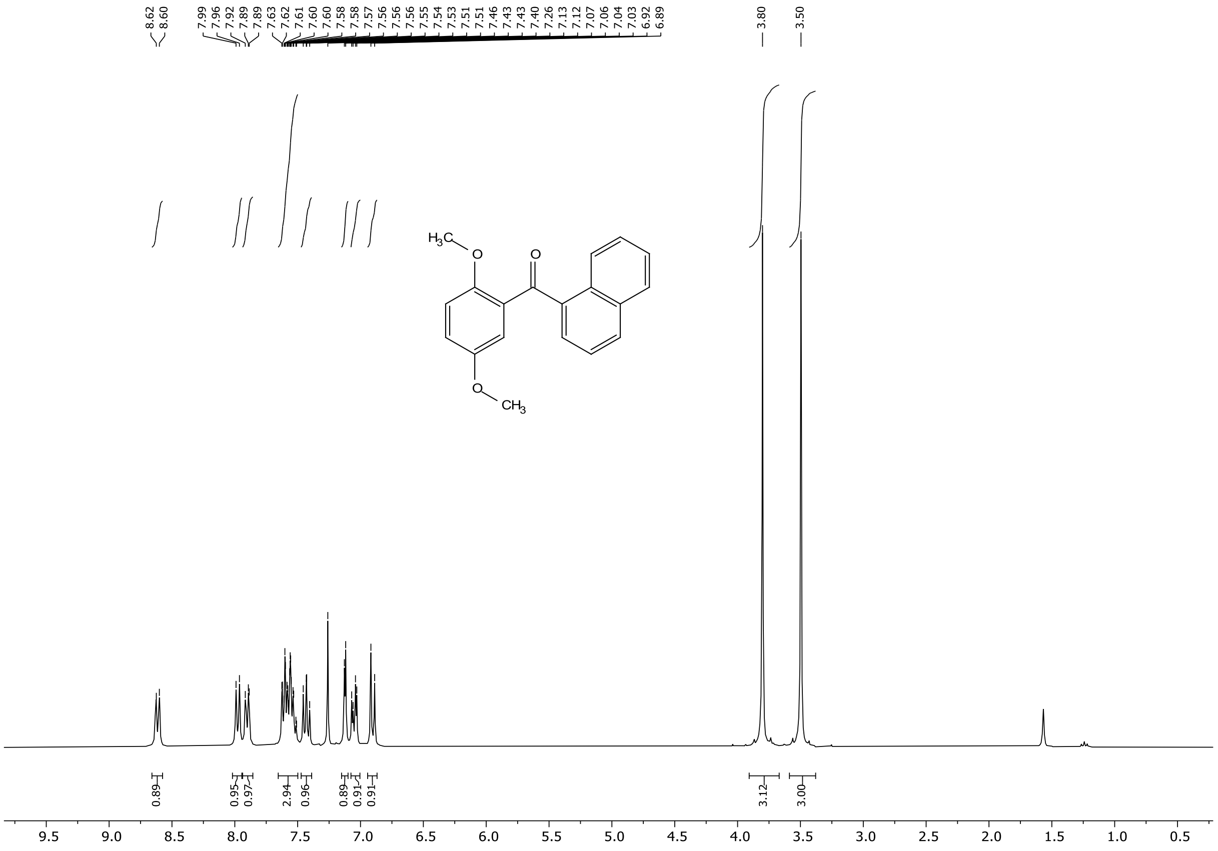

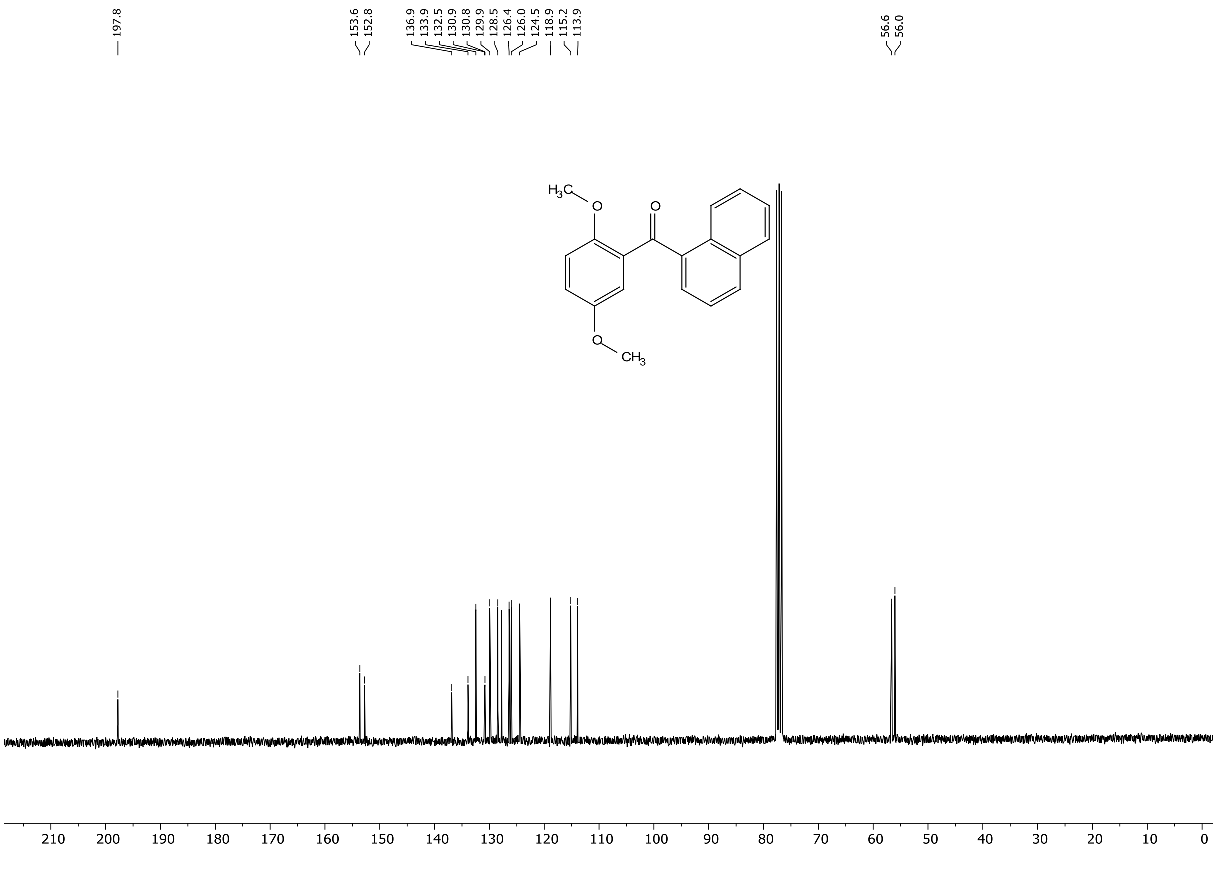

Supplement: RA-015-D5RA03638K-s001 [file RA-015-D5RA03638K-s001.pdf]
